# Comprehensive Molecular Analysis of Disease-Related Genes as First-Tier Test for Early Diagnosis, Classification, and Management of Patients Affected by Nonsyndromic Ichthyosis

**DOI:** 10.3390/biomedicines12051112

**Published:** 2024-05-17

**Authors:** Tiziana Fioretti, Fabrizio Martora, Ilaria De Maggio, Adelaide Ambrosio, Carmelo Piscopo, Sabrina Vallone, Felice Amato, Diego Passaro, Fabio Acquaviva, Francesca Gaudiello, Daniela Di Girolamo, Valeria Maiolo, Federica Zarrilli, Speranza Esposito, Giuseppina Vitiello, Luigi Auricchio, Elena Sammarco, Daniele De Brasi, Roberta Petillo, Antonella Gambale, Fabio Cattaneo, Rosario Ammendola, Paola Nappa, Gabriella Esposito

**Affiliations:** 1 CEINGE Advanced Biotechnologies Franco Salvatore, 80145 Naples, Italy; fioretti@ceinge.unina.it (T.F.); ambrosioa@ceinge.unina.it (A.A.); sabrina.vallone99@gmail.com (S.V.); felice.amato@unina.it (F.A.); federica.zarrilli@unina.it (F.Z.); speranza.esposito@unina.it (S.E.); 2 Section of Dermatology, Department of Clinical Medicine and Surgery, School of Medicine, University of Naples “Federico II”, 80131 Naples, Italy; fabriziomartora92@libero.it (F.M.); fgaudiello@yahoo.it (F.G.); luauricc@unina.it (L.A.); paonappa@unina.it (P.N.); 3 Medical and Laboratory Genetics Unit, AORN A. Cardarelli, 80131 Naples, Italy; ilaria.demaggio@aocardarelli.it (I.D.M.); carmelo.piscopo@aocardarelli.it (C.P.); roberta.petillo@aocardarelli.it (R.P.); 4 Department of Molecular Medicine and Medical Biotechnologies, School of Medicine, University of Naples “Federico II”, 80131 Naples, Italy; passaro.diego@libero.it (D.P.); valeriamaiolo1999@gmail.com (V.M.); fabio.cattaneo@unina.it (F.C.); rosario.ammendola@unina.it (R.A.); 5 Medical Genetics Unit, Department of General and Emergency Paediatrics, AORN Santobono-Pausilipon, 80122 Naples, Italy; fabio.acquaviv@gmail.com (F.A.); dandebrasi@hotmail.com (D.D.B.); 6 Department of Biology, University of Naples “Federico II”, 80126 Naples, Italy; daniela.digirolamo@unina.it; 7 Medical Genetics Unit, Integrated Care Department of Laboratory and Transfusion Medicine, Federico II Hospital, 80131 Naples, Italy; dr.giuseppina.vitiello@gmail.com (G.V.); antonellagambale@gmail.com (A.G.); 8 Pediatric Dermatology Unit, Department of Dermo-Immuno-Rheumatology Paediatrics, AORN Santobono-Pausilipon, 80122 Naples, Italy; e.sammarco@santobonopausilipon.it

**Keywords:** congenital nonsyndromic ichthyosis, molecular diagnosis, splicing variant, X-linked ichthyosis

## Abstract

Inherited ichthyoses are a group of clinically and genetically heterogeneous rare disorders of skin keratinization with overlapping phenotypes. The clinical picture and family history are crucial to formulating the diagnostic hypothesis, but only the identification of the genetic defect allows the correct classification. In the attempt to molecularly classify 17 unrelated Italian patients referred with congenital nonsyndromic ichthyosis, we performed massively parallel sequencing of over 50 ichthyosis-related genes. Genetic data of 300 Italian unaffected subjects were also analyzed to evaluate frequencies of putative disease-causing alleles in our population. For all patients, we identified the molecular cause of the disease. Eight patients were affected by autosomal recessive congenital ichthyosis associated with *ALOX12B*, *NIPAL4*, and *TGM1* mutations. Three patients had biallelic loss-of-function variants in *FLG*, whereas 6/11 males were affected by X-linked ichthyosis. Among the 24 different disease-causing alleles we identified, 8 carried novel variants, including a synonymous *TGM1* variant that resulted in a splicing defect. Moreover, we generated a priority list of the ichthyosis-related genes that showed a significant number of rare and novel variants in our population. In conclusion, our comprehensive molecular analysis resulted in an effective first-tier test for the early classification of ichthyosis patients. It also expands the genetic, mutational, and phenotypic spectra of inherited ichthyosis and provides new insight into the current understanding of etiologies and epidemiology of this group of rare disorders.

## 1. Introduction

Inherited ichthyoses are rare heterogeneous disorders of skin keratinization characterized by generalized and persistent skin dryness and scaling of variable degrees. They can involve the skin (isolate or nonsyndromic) exclusively or include extracutaneous signs (syndromic), can range from mild to sometimes life-threatening phenotypes, and can be transmitted with various Mendelian inheritance modes. Skin lesions may be generalized or occur locally, with a type of scales that widely varies in shape (lamellar, fine) and color (brown, white). A variety of other symptoms, such as xerosis, palmoplantar keratoderma, erythema, itch, and pain, all with a variable degree of severity, may also occur [1].

Over 50 genes, which also show a high degree of allelic heterogeneity, have been related to inherited ichthyosis to date. Among the isolate forms, autosomal recessive congenital ichthyosis (ARCI) is very rare, with a worldwide estimated prevalence of 1:100,000–300,000, whereas X-linked ichthyosis (XLI) is relatively common, with an estimated incidence between 1:2000 and 1:6000 male newborns [2]. As a distinguishing feature, ARCI patients are usually born encased in the collodion membrane, which may cause serious perinatal complications, leading, in some cases, to infant mortality [2]. Once the collodion membrane sheds, the skin phenotype can range from the very severe, often lethal harlequin ichthyosis (HI) to the disabling lamellar ichthyosis (LI), characterized by dark plate-like scales and congenital ichthyosiform erythroderma (CIE), with fine whitish scaling and erythroderma of variable degree of severity [2]; in some cases, collodion shedding leaves minor or no skin impairment, as in the self-improving collodion ichthyosis (SICI) phenotype [3,4].

Although HI and LI represent the more disabling phenotypes, even the relatively less severe forms of ichthyosis, such as CIE, XLI, and the common ichthyosis vulgaris (IV), may negatively influence the quality of life of patients who are especially vulnerable to social and psychological side effects [5,6].

Due to their rarity and diversity, with overlapping or heterogeneous phenotypes that evolve over the years, either spontaneously or as the result of treatment or environmental factors [1], an accurate and rapid diagnosis of ichthyosis is challenging. However, clinical and genetic classification of ichthyosis is important to identify possible associated diseases in syndromic forms, for genetic counseling and prenatal diagnosis, to follow the clinical course of the disease, and to start potential therapies. Indeed, similarly to other human diseases, including cancer, neuromuscular and cardiac disorders [7,8,9], oxidative stress and inflammation are major factors associated with the development of some inherited ichthyosis forms [10,11,12,13]; therefore, molecules targeting oxidative stress can be useful therapeutic options for the management of cutaneous symptoms and complications.

Clinical evaluation, histological and electron microscopy analysis, as well as family history are important clues for the classification of an ichthyosis form [14,15], but the correct and definitive diagnosis can be obtained when DNA analysis identifies the molecular cause of the disease. Due to the wide genetic heterogeneity, molecular analysis of multiple genes by next-generation sequencing (NGS) technology represents the most sensitive methodology to obtain the diagnosis in the greatest number of affected patients [16,17,18].

In the attempt to identify the genetic cause of the disease in 17 unrelated Italian patients referred with nonsyndromic congenital ichthyosis, without having any further details regarding family history or phenotypic features, we performed an NGS analysis focused on a panel of over 50 genes that covers most forms of isolate and syndromic ichthyosis, including XLI and IV.

## 2. Materials and Methods

### 2.1. Patients

From September 2021 to December 2023, 17 unrelated Italian patients—11 males and 6 females—aged between 0 months and 66 years, came to our attention with a clinical diagnosis of congenital ichthyosis, as assessed by dermatologists or geneticists from different hospitals in Naples, Italy. Mostly, the patient’s phenotype resulted in diffuse desquamative scaling of variable appearance (large dark brownish scales or thin whitish scales), with or without erythema of variable degree. Six patients were born encased in a collodion membrane; in 3 cases, the phenotype significantly improved after collodion shedding. In the remaining 11 patients, scaling was congenital or appeared in the first month of life. Family histories were negative for inherited diseases or any skin disorder in 13 cases. Four patients reported other affected relatives; among these, 2 pedigrees were presumed consistent with X-linked recessive inheritance, with 2 having autosomal dominant transmission. No patient declared consanguinity of parents. Parents or other relatives were recruited when available. All participants gave written informed consent before undergoing the molecular analysis, which was performed for medical purposes according to the guidelines for genetic tests approved by the Italian Ministry of Health and by a local Institutional Review Board. Patients also gave written consent to the use of their anonymized data for scientific purposes and publication. Not all patients agreed to publish photos of their phenotype.

### 2.2. Molecular Analyses

Genomic DNA was extracted from peripheral blood according to standard procedures. Mutation analysis was performed by NGS, as previously described [17]. Capture-based library preparation was carried out according to the SureSelect FocusedXT Exome CCP17 protocol (Agilent Technologies, Inc.; San Diego, CA, USA), based on a highly targeted design enabling analysis of 4800 disease-associated genes. This target-enrichment strategy followed by deep sequencing has the advantage of avoiding allele dropout due to SNPs in PCR primers or secondary DNA structures [19]. Target sequencing was performed with the NextSeq500 sequencing system (Illumina, San Diego, CA, USA). Variant calling, annotation, filtering, and prioritization were performed using the “Alissa Align and Call” and “Alissa Interpret” analysis software (Agilent). The first platform elaborates the FASTQ sequencing files, aligns sequencing reads to the reference genome (GRCh37/hg19), calculates quality control (QC) metrics, releases text files with coverage depth information of the whole targets, identifies variants, views the results in a genomic browser through the analysis of BAM files (binary alignment map), and then exports VCF (variant call format) and QC files to Alissa Interpret, which annotates and analyzes variants and compiles clinical analysis reports, sets up variant annotation and triage pipelines. The pipeline for filtering NGS data was limited to 54 genes associated with ichthyosis and erythrokeratoderma (https://panelapp.genomicsengland.co.uk/, accessed on 19 April 2024). A coverage depth of at least 20X was obtained for about 99% of the whole target. We focused on variants with a minor allele frequency (MAF) of ≤0.01, as reported in the consulted databases (gnomAD, dbSNP, ExAC, 1000 genomes v.3, accessed on 27 January 2024). To classify the identified variants, we interrogated the multiparametric bioinformatic tool Franklin (https://franklin.genoox.com, accessed on 27 January 2024) and Varsome (https://varsome.com, accessed on 27 January 2024). All known and new variants were annotated according to the HGVS recommendations and interpreted according to the American College of Medical Genetics and Genomics (ACMG) guidelines [20]. Combined Annotation Dependent Depletion (CADD) scores of putative disease-causing SNVs were calculated by the freely available tool CADD v.1.7 (https://cadd.gs.washington.edu/snv, accessed on 29 April 2024).

Putative disease-causing small nucleotide variants (SNVs) were confirmed by Sanger sequencing. The Integrative Genomics Viewer (IGV) software v.2.8.0 (https://igv.org, accessed on 29 April 2024) was used to download BAM files, visualize NGS read depth and coverage of the target regions, and identify hemizygous *STS* deletions. To detect heterozygous copy number variations (CNVs) in *ALOX12B* (NM_001139.3), relative quantification of genomic DNA sequences was carried out through an in-house procedure previously reported [17]. In brief, the read depth of each of the 15 *ALOX12B* exons was normalized to the average read depth of all the exons of a reference autosomal gene (*AGRN*) in the patient, relative to the same data obtained from three normal controls. Numerical data were obtained from the coverage text files released for each sequenced sample by Alissa Interpret, and relative gene dosage was expressed as fold change (two copies = 0.85–1.20; one copy = 0.35–0.65).

Heterozygous deletions were confirmed by quantitative PCR (qPCR) of *ALOX12B* exon 1, 3 and 15 performed in triplicate using the iCycler™ version 3.021 (Bio-Rad Laboratories S.R.L., Segrate, Italy) with primer pairs previously reported and the iQTMSYBR^®^Green Supermix (Bio-Rad Laboratories S.R.L.) [17]. Exon 2 of the human *HBB* gene was used as a reference for the copy number. Data from the proband were expressed as fold change relative to three normal subjects by the comparative 2^−ΔΔCT^ method.

Independent segregation of the identified variants was demonstrated in all cases but three.

### 2.3. Molecular Cloning in the Minigene Vector

To assess the impact of the c.2088G>A synonymous sequence variant on the correct splicing of the *TGM1* exon 13, we performed a functional assay based on a minigene construct [21,22,23]. To this aim, a 1303 nt long genomic sequence starting from intron 11 to intron 14 of the *TGM1* gene was amplified from the genomic DNA of the patient heterozygous for the variant using a forward primer (5′-ATCATTATACATATTTATGGGTACCCCAACCCAGCCAGCTC-3′) and a reverse primer (5′-GTACACATATTAAAACATTACACTGACATACTTGTCCAGAC-3′) containing the Kpn I and XhoI restriction sites, respectively. The amplified fragment was purified from agarose gel using the NucleoSpin™ Gel and PCR Clean-up Kit (#740609, Macherey-Nagel GmbH & Co. KG, Dueren, Germany), according to the manufacturer’s instructions, and then cloned by homologous recombination by the NEBuilder HiFi DNA Assembly Cloning kit (#E5520, New England Biolabs, Ipswich, MA, USA) into a pMGene vector that contained the entire β-globin (*HBB*) gene downstream to the cytomegalovirus promoter; a KpnI restriction site, useful for cloning the opportune exogenous insert, was present in the middle of *HBB* intron 2 [22,23]. Sanger sequencing was performed to verify the correct insertion of the *TGM1* sequences and to identify wild-type and mutated recombinant plasmids.

### 2.4. Cell Culture, Minigene Expression and Transcript Analysis

To evaluate the possible splicing alteration associated with c.2088G>A synonymous variant, wild-type and mutated recombinant *HBB-TGM1* chimeric gene previously cloned in the pMGene vector were expressed in a eukaryotic cell line. To this aim, HeLa cells were grown in a 5% CO_2_ incubator at 37 °C in Dulbecco’s modified Eagle’s medium (Invitrogen, Life Sciences), supplemented with 10% fetal bovine serum (Hyclone, Thermo Scientific, Logan, UT, USA). Cells were seeded at 6 × 10^5^ per well in 60-mm dishes for 16 h and then transfected with 5 µg of each pMGene construct by calcium phosphate method. Twenty-four hours after transfection, cells were collected, and total RNA was extracted using the MasterPure Complete DNA and RNA Purification Kit (Epicenter Biotechnologies, Madison). Then, 1 μg of total RNA was reverse transcribed using Invitrogen™ M-MLV Reverse Transcriptase (Fisher Scientific Italia, Segrate, Italy), according to the manufacturer’s instructions, and 100 ng of cDNA from normal and mutant clones were amplified using the following primers: RT-TGM12-FOR, 5′-CACATGGTCCTGCTGGAGTTC-3′; RT-MGene-REV, 5′-CCTGCACTGGTGGGGTGAATTC-3′. This primer pair flanked the hybrid cDNA region, including *TGM1* exon 12 and *HBB* exon 3. The resulting PCR products were analyzed on a 2% agarose gel and then sequenced using the RT-MGene-REV primer.

### 2.5. Statistical Analysis

Statistical analysis was performed with GraphPad Prism software version 7.0 (https://www.graphpad.com/, accessed on 29 April 2024). Data are presented as means ± SEM. The one-way analysis of variance (ANOVA) was used to determine significant differences between groups. Data were considered statistically significant with *p* < 0.05.

## 3. Results

To demonstrate that multigene NGS can be a crucial aid for rapid classification of different forms of inherited ichthyosis, we analyzed 17 unrelated Italian probands with a generic diagnosis of congenital nonsyndromic ichthyosis, as assessed by a dermatologist and/or a geneticist. Details of the phenotypes were analyzed retrospectively.

For all patients (11 males and 6 females), NGS identified the molecular cause of the disease. Overall, we identified 24 different disease-causing alleles, including 8 with novel sequence variants and 2 variants that, although reported in public databases, had not been previously associated with the disease (Table 1).

Notably, male patients were about twice as common as females and, in agreement with the relatively higher frequency of XLI compared to the very rare autosomal recessive forms, 6 males had large deletions that removed in part or all of the *STS* gene. The remaining 11 patients had mutations of *ALOX12B*, *NIPAL4*, *TGM1*, and *FLG*. In these genes, we identified 12 different alleles, 11 of which were classified as pathogenic (Class 5) or likely pathogenic (Class 4), according to the ACMG standards (Supplementary Table S1).

Eight patients had a mutation of ARCI-related genes, and 6 were born encased in the collodion baby (CB) membrane. Seven were compound heterozygotes; the only homozygote did not declare consanguinity of parents.

**Figure 1:**
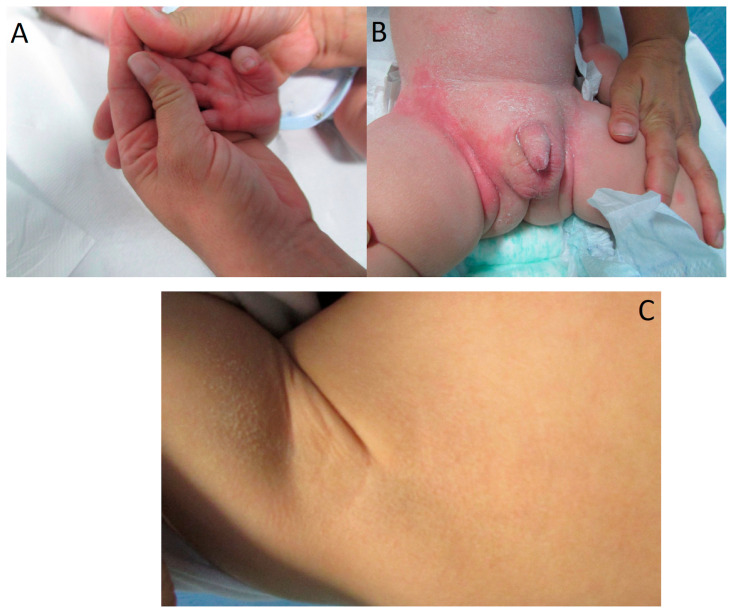
*ALOX12B*-related phenotypic features in two affected patients. (**A**) Palmar hyperkeratosis, and (**B**) patchy erythema associated with white scales appeared after collodion shedding in patient ID.02; (**C**) dyskeratosis, with pigmented areas of cutaneous xerosis, on the axillary region of patient ID.04.

**Figure 2:**
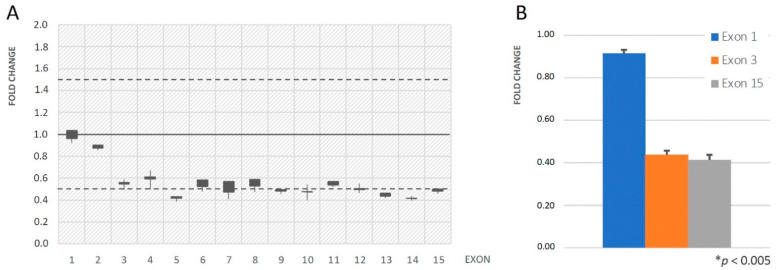
Gene dosage analysis of *ALOX12B* coding exons in patient ID.04. (**A**) Read depth evaluation for each of the 15 *ALOX12B* exons, normalized to the median read depth of all the exons of an autosomal gene (*AGRN*) (see Materials and Methods), and represented as fold change with respect to three normal controls. (**B**) Real-time qPCR analysis of exon 1, 3, 15 in patient ID.02; signal quantification is normalized to the *HBB* gene exon 2 and represented as fold change with respect to the mean values from two normal controls (2^−ΔΔCt^ method). * *p* < 0.005 compared to the normal controls.

Pathogenic variants in the *ALOX12B* gene (NM_001139.3) were detected in 50% of ARCI patients. *ALOX12B*-related phenotypes in our patients were heterogeneous and characterized by congenital mild/moderate erythroderma on the face and trunk, hyperlinearity of the hands, thin white or brownish scales on trunk and legs (Figure 1A–C); in two cases (ID.01 and ID.02), the phenotype evolved as SICI, one of these with fully normal skin. In this gene, we identified 8 different alleles, including 7 with missense changes and 1 large deletion removing exon 3-15 [17]. Three variants, namely c.1259G>A (p.Cys420Tyr), c.1907G>T (p.Ser636Ile), and c.1909C>G (p.Arg637Gly), were novel. The variant c.1259G>A (p.Cys420Tyr) was classified as likely pathogenic (Class 4) since it met four ACMG pathogenicity criteria (PM1, PP2, PM2, PM5) [19], including the report in the ClinVar database of the likely pathogenic variant c.1258T>G that involved the same amino acid residue (p.Cys420Gly) (ClinVar#VCV000808221.20); CADD score of this variant was 27.1 (Supplementary Table S1). Also, the c.1907G>T (p.Ser636Ile) variant was classified as likely pathogenic (Class 4) as it met four ACMG pathogenicity criteria (PP2, PP3, PM2, PM3). Indeed, it was not found in the consulted databases and was detected in trans with a known pathogenic variant; moreover, Ser636 to Ile amino acid substitution was evaluated as a non-conservative change, and in silico analysis predicted this variant was probably damaging to the protein structure/function, with a CADD score of 26.8 (Supplementary Table S1). In contrast, the adjacent c.1909C>G (p. Arg637Gly) change was classified as a variant of uncertain significance (Class 3) because it met only three ACMG pathogenicity criteria (PM2, PM3, PP2) and had a CADD score of 15.3 (Supplementary Table S1). It was found in trans with a known pathogenic large deletion removing exons 3–15 of *ALOX12B* (Figure 2A,B), in a child (case ID.04) with congenital dyskeratosis, pigmented areas of cutaneous xerosis located in the axillary region (Figure 1C), on the sides and the legs bilaterally, and very mild face erythroderma.

This patient was heterozygous for an additional very rare Class 3 missense variant, i.e., c.2920G>A (p.Gly974Ser), in the *RTEL1* gene, which is associated with autosomal dominant dyskeratosis congenita. Notably, the mother affected by cutaneous xerosis carried the same rare *RTEL1* variant.

**Figure 3:**
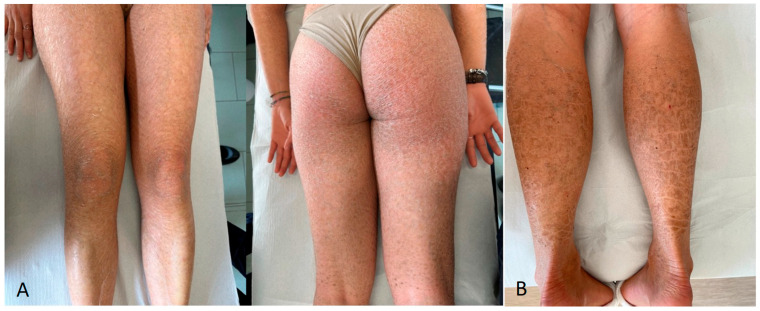
*NIPAL4*-related phenotypic features in the two affected patients. Both patients showed congenital ichthyosis characterized by widespread, dark brown or brownish, polygonal, and large scales, erythema, and generalized dryness. (**A**) Patient ID.09 was not born with collodion membrane, despite being homozygous for a nonsense variant; (**B**) detail of the legs of patient ID.10, with the dark brown, polygonal, large scales that appeared after collodion shedding.

Two ARCI patients (ID.08 and ID.09) had pathogenic variants in *NIPAL4* (NM_001099287.1). One of them was homozygous for the novel nonsense variant c.137G>A (p.Trp46*); the second patient was heterozygous for the known pathogenic variant c.398C>A (p.Pro133His) [16] and the novel frameshift pathogenic variant c.514_515dup (Met172Ilefs*). Only one *NIPAL4*-positive patient was a CB; however, both showed a severe CIE phenotype with large brownish scales and erythroderma on the whole body (Figure 3A,B).

Two further patients were compound heterozygous for *TGM1* (NM_000359.3) pathogenic variants. One child was born with CB and was compound heterozygous for the known pathogenic variant c.377G>A (p.Arg126His) and c.968G>A (p.Arg323Gln), previously associated with a SICI or BSI; he was also heterozygous for the known loss-of-function (LOF) variant c.1501C>T p.(Arg501*) in *FLG*, the gene associated with the mild autosomal dominant IV. Notably, in the patient’s family anamnesis, the mother reported the presence of cutaneous xerosis for herself and her father, whereas the maternal grandfather had psoriasis. Despite the mutation of 2 different ichthyosis-related genes, the patient’s phenotype after collodion shedding was consistent with SICI.

**Figure 4:**
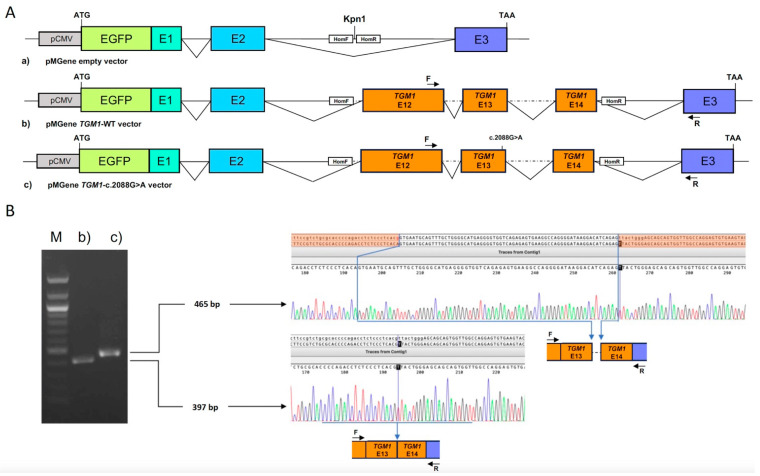
Minigene splicing assay of the *TGM1* synonymous variant c.2088G>A p.(Thr696=). (**A**) Schematic representation of empty (a), recombinant wild-type (b), and mutant (c) pMGene minigene vectors. Different boxes represent the cytomegalovirus promoter (pCMV), the enhanced green fluorescent protein (EGFP) coding sequence, *HBB* exons (E1, E2, and E3), and *TGM1* exons. Solid lines between exons represent *HBB* introns, dotted lines, and *TGM1* introns. Arrows represent the forward (F) and reverse (R) primer used for the splicing pattern analysis. HomF and HomR are the homology regions for recombination-based cloning. (**B**) Agarose gel electrophoresis of RT-PCR products relative to the mRNA expressed by *TGM1* wild-type (b) and mutant (c) recombinant minigenes in transfected HeLa cells. Sanger sequencing of the RT-PCR products shows that the mutant (465 bp long) cDNA fragment retains the first 68 nucleotides of *TGM1* intron 13 (dotted line), compared to the sequence of the wild-type (397 bp) cDNA.

The second *TGM1*-positive patient was an adult male affected by severe LI, who was heterozygous for the known missense variant c.1166G>A (p.Arg389His) [3] and for the synonymous variant c.2088G>A (p.Thr696=). The latter variant was not reported in any public databases, and bioinformatic analysis classified it as a Class 3 variant because it met three ACMG criteria (PM2, PM3, PP3); however, it involved the last nucleotide of the *TGM1* exon 13, and therefore it could be considered a putative splicing variant (Supplementary Figure S1A). To assess the deleterious role of the c.2088G>A change on the primary transcript maturation, we performed a minigene assay by cloning the *TGM1* genomic sequences from exon 12 to exon 14 in an opportune plasmid vector (Figure 4A).

We found that HeLa cells transfected with the mutant clone expressed an aberrant transcript that included the first 68 nucleotides of intron 13 (Figure 4B). This aberration resulted in a shift of the normal *TGM1* reading frame that gave rise to a premature termination codon. Therefore, the c.2088G>A variant likely represented a null allele, which was in agreement with the patient’s severe LI phenotype.

Three patients with congenital ichthyosis without CB had biallelic LOF variants in *FLG*. In these patients, we found 4 different alleles, including 2 novel variants. They had congenital ichthyosis phenotypes of variable severity that overlapped those observed in our ARCI patients.

In the 6 male patients diagnosed last with XLI, NGS identified various rare heterozygous SNVs in autosomal ichthyosis-related genes (Table 1). However, none of these variants, which were mostly classified as Class 3 according to the ACMG standards, justified the patients’ phenotype. Therefore, in the suspect of XLI, we performed a visual inspection of NGS data by the IGV software, which revealed no reads for all or part of the *STS* gene (Supplementary Figure S1B). In four patients, the deletion involved the whole *STS* gene and part of the adjacent *PDUP* gene; the remaining two patients had deletions removing exons 1–3 and 10–11 of *STS* (NM_000351.7), respectively.

In addition to the putative disease-causing genomic variants, multigene analysis revealed heterozygous rare sequence variants in at least another ichthyosis-related gene in 15/17 patients. Sixteen variants were identified in *ABCA12*, *ALOX3*, *GJB2*, *LSS*, *PNPLA1*, *RIN2*, *SERPINB8*, *ST14*, *SULT2B1*, *SUMF1*, *TCHH*, *TGM1*, and *TGM5* genes, associated with autosomal recessive transmission (Table 1). According to the ACMG standards, 75% of these variants were variants of uncertain significance (Class 3); two pathogenic (Class 5) variants were identified in *TCHH* and *LSS*, genes associated with uncombable hair syndrome, and macrocephaly, alopecia, cutis laxa, and scoliosis, respectively. Five variants, including one Class 5 and two Class 3, were found in 4 genes associated with autosomal dominant transmission, namely *FLG*, *KRT1*, *GJB4*, and *RTEL1*.

To generate a priority list of the ichthyosis-related genes that, in our population, could carry a significant number of putative disease alleles and novel variants, we looked for rare variants in the ichthyosis-related genes of 300 Italian subjects without any sign of the disease. We selected only variants that were located within the gene coding or in the splicing consensus sequences and that, to minimize any presumed influence that population stratification could have on the genetic analysis, had a gnomAD minor allele frequency (MAF) < 0.01 in individuals of European descent. In 205 of these unaffected subjects, we identified 357 rare alleles that carried 243 different rare sequence variants, all at the heterozygous state (Supplementary Tables S2–S4). Mostly, the identified sequence variants led to missense changes, but 8 alleles that carried nonsense/frameshift variants were also identified (Supplementary Tables S1–S3). Notably, the relatively common nonsense variant c.1464T>A p.Tyr488* in *PNPLA1* (gnomAD MAF = 1.1%) was identified in about 5.7% of the analyzed subjects. Among the ARCI-related genes, *ABCA12* was the most variable, with 21 different heterozygous variants identified in 31 subjects (10.3%), followed by *TGM1* and *ALOXE3*, each with 12 different rare alleles identified in 14 (4.7%) and 13 (4.3%) subjects, respectively. Eleven rare variants in *NIPAL4* were identified in 16 subjects, including 2 heterozygotes for the pathogenic c.86C>A (p.Ser29*), which therefore had a relative MAF = 0.003 in the analyzed subgroup of subjects. Similarly, 11 rare variants, including the novel nonsense c.145A>T (p.Lys49*), were found in the *ST14* gene of 14 subjects. Furthermore, 8, 7, and 6 different rare variants were identified in *ALOX12B*, *CYP4F22*, and *LIPN*, respectively, including the pathogenic p.Asp477Glufs* in *ALOX12B* and p.Gly101Glufs* in *LIPN*. Lastly, 3 variants, including the nonsense p.Glu295*, were found in *ABHD5*, associated with Chanarin–Dorfman syndrome (aka neutral lipid storage disease with ichthyosis); and 5 variants, including the known pathogenic p.Asp221Asn allele, in *ALDH3A2*, associated with Sjogren–Larsson syndrome.

## 4. Discussion

Although the potential of massive sequencing in the molecular diagnosis of inherited ichthyosis is well known, the diagnostic gold standard is still mainly focused on a careful clinical evaluation/classification to guide the identification of the genetic mutation [16]. However, anamnesis, physical examination, microscopy, and laboratory examination can be variable due to the genetic and phenotypic heterogeneity of different ichthyosis forms, thus making the molecular diagnosis mandatory for ichthyosis classification [24] and for differential diagnosis [25].

Previous studies indicate, however, that the detection rate of multigene sequencing for ichthyosis-related disease variants is approximately 80–90% [1]. This limitation is partly attributed to the low sensitivity of NGS in detecting large deletions/duplications, despite the recent technical advances combining assessment of CNVs and SNVs in the same analysis [26].

In our cohort of 17 unrelated probands with congenital ichthyosis, multigene NGS analysis allowed the molecular classification of all the referred cases. The high success rate of our methodology was mostly due to a data analysis strategy that, in addition to known and new disease-causing SNVs, was able to reveal heterozygous and hemizygous large deletions, therefore confirming elevated sensitivity also for the detection of these types of CNV [17]. Indeed, as very recently reported [27], accessibility to raw sequence data and manual review of suspicious sequence regions allows for reducing false-negative results in the clinical application of NGS. Considering, however, the limited sample size and issues such as insufficient coverage depth of a few target regions, our results do not provide sufficient basis for a broad conclusion regarding the real efficacy of this genetic testing approach.

Nevertheless, we were able to molecularly classify 8 ARCI patients, 6 males affected by XLI, and 3 patients with biallelic LOF variants in *FLG*. Patient phenotypes were retrospectively analyzed and related to the identified genotype (Table 1).

**Figure 5:**
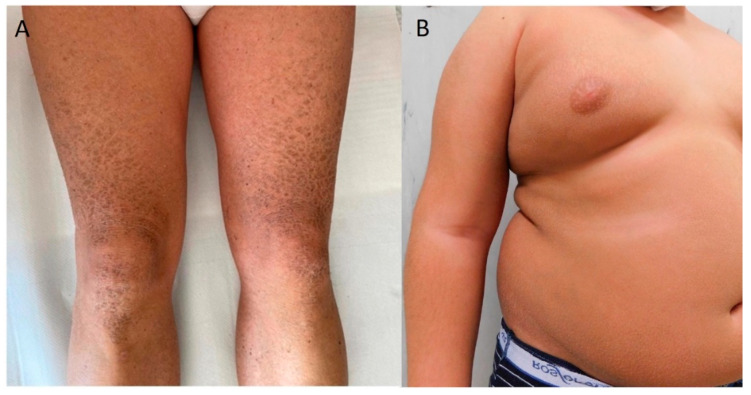
*STS*-related phenotypic features in two affected males. (**A**) Details of the legs, with large brownish, polygonal scales, in patient ID.13, with deletion of *STS* exons 10 and 11; (**B**) mild desquamation and mild erythema of the trunk in patient ID.16, with deletion of the whole *STS* gene.

Male patients were about twice as many as females and, in agreement with the known relatively higher frequency of XLI compared to the autosomal recessive forms, more than 54% of them had large deletions that removed in part or all the *STS* gene encoding the steroid sulfatase enzyme. Only one pedigree was consistent with X-linked inheritance, whereas the other patients had an unremarkable family history. Clinical phenotypes of XLI patients were also unremarkable, with congenital onset characterized by large, widespread brownish scales resembling LI or fine brownish scaling with or without erythroderma (Figure 5). The relatively high number of XLI in our patients’ cohort has undoubtedly contributed to increasing the diagnostic yield of our procedure. Similar to other X-linked disorders, XLI is mostly caused by deletions (80–90%), but about 10–20% of patients have pathogenic SNVs [28,29,30]. Therefore, multiple ligation-dependent probe amplification (MLPA) and array comparative genomic hybridization (aCGH) are the methods of choice for XLI diagnosis, even though they cannot detect pathogenic SNVs [30,31]. In contrast, an NGS-based analytical strategy can detect all the possible alterations affecting the *STS* gene, as well as possible modifier variants in other related genes. Moreover, the multigene panel we used, by covering all the known disease genes located on the X chromosome, can identify the rarest male patients affected by syndromic XLI due to large genomic deletions, including *STS* and its contiguous genes [30,31]. Notably, males with XLI are at increased risk of developing attention deficit hyperactivity disorder, mood disorders, and motor and cardiac problems [30,32,33]. As the *STS* gene escapes X-inactivation, female carriers of XLI have reduced *STS* expression/activity and could manifest similar behavioral phenotypes to males with XLI. Additionally, as *STS* activity normally increases in female tissues towards late pregnancy and into the puerperium, female carriers are at increased risk of psychopathology, especially in the postpartum period [32,33]. Therefore, XLI molecular diagnosis implies the need to provide multidisciplinary genetic counseling and specialist clinical care to patients and their families.

Of the remaining 10 patients, 8 were affected by ARCI. Differently from previous reports indicating that *TGM1* was a major causative ARCI gene [34,35], we found pathogenic variants in *ALOX12B* in 50% of our patients, whereas *TGM1* and *NIPAL4* were involved in 25% of cases each. This is, however, in line with our and other studies performed in families of Italian descent with CIE phenotypes [17,30], whereas *TGM1* pathogenic variants are primarily associated with LI in Italian patients and patients of various ethnic descent [4,35,36,37]. Consistently, our *ALOX12B*-positive patients showed CIE phenotypes of mild/moderate severity. One child patient (ID.01 in Table 1), who evolved as SICI, was compound heterozygous for two known variants previously associated with classic CIE or SICI [17,38,39]. Another child was heterozygous for the novel c.1907G>T (p.Ser636Ile) change and the known c.1192C>T (p.His398Tyr) variant in *ALOX12B* and also carried a novel heterozygous rare variant of uncertain significance (Class 3) c.1033G>A p.(Asp345Asn) in *TGM1* (ID.02 in Table 1). Despite the presence of the *TGM1* variant, the CIE phenotype, which was initially characterized by palmoplantar keratoderma, ectropion, and large scaling on the trunk and the face (Figure 1A,B), evolved in SICI with normal skin in this patient. It is worth noting that the patient’s unaffected mother had a first cousin with a clinical diagnosis of LI, suggesting a family history consistent with X-linked inheritance rather than the autosomal recessive form finally diagnosed.

The phenotype was very mild for the patient (Figure 1C) who was compound heterozygous for a large intragenic deletion removing exon 3–15, encoding the catalytic domain of the *ALOX12B* gene product, i.e., the 12R-LOX enzyme, which oxidates linoleic acid to ceramide and participates in the epidermal lipid synthetic pathway, leading to the formation of corneocyte lipid envelope and extracellular lamellar membranes [40]. We previously found this large deletion at the homozygous state in another ARCI patient that evolved as SICI [17]. The patient herein analyzed carried on the other allele, the missense variant c.1909C>G (p.Arg637Gly), the only novel variant classified as Class 3 by the bioinformatic tools. In this patient, the very mild form of ARCI associated with the deleted allele supports the previous hypothesis [17,41] that 12R-LOX deficiency alone is necessary but not sufficient to hold severe the ichthyosis phenotype and suggests that the c.1909C>G variant might be a hypomorphic allele.

The two ARCI patients with *NIPAL4* mutation had very severe CIE phenotypes, characterized by large brownish widespread scaling (Figure 3A,B), which were related to the presence of 2 novel null alleles, i.e., the nonsense p.Trp108* at the homozygous state (Figure 3A), and the frameshift p.Ser215Argfs* in trans with the frequent pathogenic missense variant c.527C>A (p.Ala176Asp) (Figure 3B), which has been already reported in Italian patients [16]. Unlike what was recently reported, our *NIPAL4*-positive patients did not show any psoriasis-like features [42]. Ichthyin, the *NIPAL4* gene product, is a putative Mg^2+^ transporter predominantly expressed in the upper epidermis; nonsense and frameshift mutations often result in absent gene product, whereas the p.Ala176Asp variant mislocalizes in the periphery and cytoplasm of the cell [43]. The aberrant localization of the p.Ala176Asp mutant, which is consistent with a loss of function, may be a feature of this mutant that worsens the phenotype of our compound heterozygous patient who, unlike the patient homozygous for the p.Trp108*, was born as CB.

As previously reported, missense variants in *TGM1* can evolve to SICI, which is the phenotype of our patient who was compound heterozygous for the known c.968G>A p.(Arg323Gln) and c.377G>A p.(Arg126His) variants [3,33]. Indeed, he had minimal evidence of ichthyosis, with residual very slight fissures in the skin folds, absence of hyperlinearity in the palms and soles, and slight skin flaking persisting only on the back. The p.Arg323Gln variant has been reported in multiple individuals affected by SICI [4,44]; in contrast, the c.377G>A substitution has been mostly observed in individuals with LI, in agreement with functional studies demonstrating that p.Arg126His change results in absent TGase1 enzyme activity [45,46,47]. In this patient, not even the additional presence of the *FLG* pathogenic variant c.1501C>T (p.Arg501*), of maternal origin, has contributed to worsening his benign SICI phenotype. A recently reported child with CB, who was compound heterozygous for the c.944 G > A (p.Arg315His) and c.1025 G > T (p.Trp342Leu) pathogenic variants and carried the LOF variant *FLG*:c.4768C>T (p.Gln1590*) and also evolved in a mild SICI phenotype, however, showed persistence of eczematous patches and scaly plaques [48], which were not present in our patient.

A classic LI phenotype appeared after collodion sheading in the patient that carried the c.1166G>A (p.Arg389His), previously found in patients affected by a mild form of LI [49], and the novel synonymous variant c.2088G>A (p.Thr696=) in *TGM1*. The filtering process of NGS data often overlooks synonymous variants. However, synonymous variants affecting exon-intron borders have been previously found in *TGM1* by RNA-seq analysis [3,50]. Notably, in the ichthyosis-related genes of normal as well as affected subjects, NGS identifies, on average, 20 rare variants, including the synonymous and intronic ones. This relatively small number of nucleotide changes allows an easy evaluation of each variant, also through visualizing their position within the gene structure. We thus uncovered that the synonymous c.2088G>A (p.Thr696=) was located at the border of exon-intron 13 and could, therefore, affect RNA splicing. This hypothesis was confirmed by in vitro minigene assay revealing that c.2088G>A caused an aberrant splicing, which resulted in the retention of 68 nucleotides of intron 13 in the mutant mRNA (Figure 4B), leading to a premature termination codon. In our patient, the c.2088G>A allele also carried in cis the c.1559A>G (p.Glu520Gly) variant that, although previously considered a pathogenic variant [35], is currently classified as benign polymorphism.

Heterozygous LOF variants in *FLG* cause IV, the most common (1/250) noncongenital form of ichthyosis, which has also been associated with the prevalence and persistence of atopic dermatitis [51]. Consequently, the *FLG* gene is not always included in the panel of genes to be analyzed for the molecular diagnosis of the rarest forms of ichthyosis. Interestingly, we found *FLG* biallelic LOF variants, including 4 nonsense and 2 frameshift variants, in three patients with LI and CIE phenotypes of variable severity that overlapped those observed in other ARCI patients (not shown). In two cases, congenital appearance and unremarkable family history were consistent with autosomal recessive transmission. The remaining patient, who also suffered from impetigo, reported mildly affected mother and daughter. Overall, these data confirm that *FLG* LOF variants are associated with a semidominant mode of inheritance with incomplete penetrance and that the very rare homozygotes and compound heterozygotes are more severely affected than symptomatic heterozygotes [52]. Therefore, *FLG* analysis should be included in the differential diagnosis of inherited ichthyosis. Interestingly, since nonsense mutations are the most frequent pathogenic variants of *FLG* [53] and, although more rarely, of other ichthyosis-related genes, nonsense suppression therapy could be a promising approach also for the treatment of patients affected by hereditary skin diseases [54].

In addition to the primary cause of the disease, multigene NGS analysis revealed sequence variants in other genes related to isolated/syndromic ichthyosis. In our patients, we identified 23 additional variants in 12 and 5 different genes associated with autosomal recessive and autosomal dominant transmission, respectively (Table 1). Although these variants could act as genetic modifiers of the phenotype, none of them seems to exacerbate/improve the ichthyosis phenotype in our patients.

Based on the evidence that our NGS analysis detected an average of 1.46 rare variants in the ichthyosis-related genes of 300 unaffected Italian individuals, we generated a priority list of the ichthyosis-related genes that, in our population, showed a significant number of putative disease alleles and novel variants (Supplementary Tables S1–S3). In this group of subjects, we identified 357 rare alleles (gnomAD MAF<0.01, in the European descent) that carried 246 different SNVs, including 11 pathogenic/likely pathogenic variants in *ABHD5*, *ALOX12B*, *ALOXE3*, *LIPN*, *NIPAL4*, *PHYH*, *SLC27A4*, *ST14*, and numerous variants of uncertain significance (Class 3). Despite the fact that population stratification could act as a confounder in our genetic analysis [55], the relatively high frequency of Class 3 variants in our homogeneous group of Italian subjects strongly suggests that inherited ichthyoses are more frequent than that reported; however, due to the wide clinical heterogeneity that can vary even over the years, also as a consequence of environmental factors or mosaicism [1], patients with ichthyosis could often escape clinical attention or be misclassified. This latter consideration is also supported by the high prevalence (90%) of compound heterozygotes identified in our cohort of ichthyosis patients compared to homozygotes [4,16].

## 5. Conclusions

Due to their rarity and diversity, with overlapping or heterogeneous phenotypes that evolve over the years, an accurate and rapid classification of congenital ichthyosis is challenging. Multigene NGS analysis is the method of choice for the comprehensive molecular testing and early classification of patients affected by any type of ichthyosis, including XLI and IV. Indeed, we herein demonstrate that targeted quantitative analysis of NGS data of a specific gene, by identifying heterozygous and homo/hemizygous CNVs, partly resolves missing heritability problem, therefore improving diagnostic sensitivity of the whole procedure, with useful implications for clinical practice. Effective cooperation between dermatologists, molecular biologists, and geneticists has been crucial in improving the diagnostic flow and care of our patients, providing them with definitive diagnosis, offering genetic-awareness counseling, and potential enrolment in disease-specific therapeutic trials as they become available.

Moreover, our comprehensive molecular analysis expands the genetic, mutational, and phenotypic spectra of inherited ichthyosis in the Italian population and provides new insight into the current understanding of etiologies and epidemiology of this group of rare and often underdiagnosed diseases, therefore providing new avenues for future research, especially those based on the discovery of genome-guided and/or targeted therapies.

## Figures and Tables

**Table 1:** Skin-related phenotypes and genetic data in patients affected by different forms of isolate ichthyosis.

Patient ID	Age (Years)	Sex	Phenotype	Gene	Variant Allele	ACMG
01	3	F	CB–SICI with normal skin	*ALOX12B*	c.47C>T p.(Ser16Leu)	Class 5
					c.1798C>T p.(Arg600Trp)	Class 4
02	3	M	CB–SICI—Familial	*ALOX12B*	c.1907G>T p.(Ser636Ile) †	Class 4
			Palmar-plantar keratoderma		c.1192C>T p.(His398Tyr)	Class 5
			ectropion, trunk/face scaling	*TGM1*	c.1033G>A p.(Asp345Asn)	Class 3
03	50	F	CB–CIE—generalized dryness	*ALOX12B*	c.1259G>A p.(Cys420Tyr) †	Class 4
			face erythroderma, widespread whitish		c.1642C>T p.(Arg548Trp)	Class 5
			fine scales	*RIN2*	c.364C>T p.(His122Trp)	Class 3
				*SULT2B1*	c.912G>A p.(Met304Ile)	Class 2
04	3	M	CIE—congenital dyskeratosis	*ALOX12B*	c.1909C>G p.(Arg637Gly) †	Class 3
			cutaneous xerosis, mild face erythroderma		EX3_15del	Class 5
				*TGM5*	c.850G>A p.(Val284Ile)	Class 3
				*RTEL1* ^‡^	c.2920G>A p.(Gly974Ser)	Class 3
05	33	F	CIE—Dark brownish, polygonal scales	*FLG* ^‡^	c.6034C>T p.(Gln2012*)	Class 4
			generalized dryness		c.2282_2285del p.(Ser761Cysfs*)	Class 4
				*TGM1*	c.2405A>T p.(Asp802Val)	Class 1
06	71	M	LI—Widespread dark brown,	*FLG* ^‡^	c.1501C>T p.(Arg501*)	Class 5
			polygonal scales impetigo/familial,		c.5186C>G p.(Ser1729*)	Class 5
			frequent skin infections, nail dystrophy	*ALOXE3*	c.122C>A p.(Ala41Asp)	Class 3
07	5	F	CI—ichthyosiform xerosis, spread to the trunk and limbs,	*FLG* ^‡^	c.5980delC p.(His1994Metfs*) †	Class 5
			palmar-plantar hyperkeratosis		c.1951G>T p.(Glu651*)	Class 5
08	50	M	CB–CIE	*NIPAL4*	c.398C>A p.(Pro133His)	Class 5
					c.514_515dup p.(Met172Ilefs*) †	Class 5
				*TCHH*	c.4005_408del p.(Asp1335Glufs*)	Class 5
09	29	F	No CB—congenital widespread,	*NIPAL4*	c.137G>A p.(Trp46*) †	Class 5
			dark brown, polygonal scales;		c.137G>A p.(Trp46*) †	Class 5
			generalized dryness, nail dystrophy,	*ABCA12*	c.6593C>T p.(Thr2198Ile)	Class 3
			frequent skin infections	*SERPINB8*	c.988G>A p.(Ala330Thr)	Class 3
10	2	M	CB—SICI	*TGM1*	c.377G>A p.(Arg126His)	Class 5
					c.968G>A p.(Arg323Gln)	Class 5
				*FLG* ^‡^	c.1501C>T p.(Arg501*)	Class 5
				*LSS*	c.1307A>G p.(Asn436Ser)	Class 3
11	22	F	CB–LI—Large scales and plates, adherent	*TGM1*	c.1166G>A p.(Arg389His)	Class 5
			to most of the body surface, ectropion,		c.2088G>A p.(Thr696=) †	Class 5
			ear anomalies, nail dystrophy	*SUMF1*	c.376A>C p.(Met126Leu)	Class 3
			palmar-plantar hyperkeratosis, erythroderma	*GJB2* ^‡^	c.663G>C p.Lys221Asn	Class 3
12	14	M	CI—xerosis of the face	*STS*	EX1-3del	Class 5
			elbows/knees hyperkeratosis	*KRT1* ^‡^	c.1724A>G p.(His575Arg)	Class 3
13	32	M	CI—generalized dryness; widespread,	*STS*	EX10-11del	Class 5
			dark brown, polygonal scales; mother affected	*GJB4* ^‡^	c.500A>G p.(Glu167Gly)	Class 2
14	6	M	CI	*STS*	EX1-12del	Class 5
				*ALOX3*	c.122C>A p.(Ala41Asp)	Class 3
15		M	CI	*STS*	EX1-12del	Class 5
				*ST14*	c.2146G>C p.(Glu716Gln)	Class 3
16	9	M	CI—face and trunk desquamation	*STS*	EX1-12del	Class 5
			elbows/knees hyperkeratosis	*ABCA12*	c.6704A>C p.(Glu2235Ala)	Class 1
			mild erythema improved with growth			
17	22	M	CI—Familial	*STS*	EX1-12del	Class 5
				*PNPLA1*	c.1464T>A p.(Tyr488*)	Class 1
				*KRT1* ^‡^	c.1669A>G p.(Ser557Gly)	Class 1

† novel variants identified in this study; ^‡^ gene associated with autosomal dominant transmission. ACMG: American College of Medical Genetics and Genomics classification; CB: collodion baby; CI: congenital ichthyosis; CIE: congenital ichthyosiform erythroderma; LI: lamellar ichthyosis; SICI: self-improving congenital ichthyosis.

## Data Availability

The data that support the findings of this study are available within the article.

## Supplementary Materials

The following supporting information can be downloaded at: https://www.mdpi.com/article/10.3390/biomedicines12051112/s1, Figure S1: Integrative Genomics Viewer visualization of NGS data; Table S1: ACGM pathogenic criteria for small variant (SV) classification and their relative Combined Annotation Dependent Depletion (CADD) scores. Table S2: Overall number of rare variants identified in the ichthyosis-related genes of 300 unaffected individuals; Table S3: Number of different rare variants identified in ichthyosis-related genes of 300 unaffected individuals; Table S4: Subjects with at least one rare variant in ichthyosis-related genes among 300 unaffected individuals.
