# Supplementary material for: Comprehensive Molecular Analysis of Disease-Related Genes as First-Tier Test for Early Diagnosis, Classification, and Management of Patients Affected by Nonsyndromic Ichthyosis
Source: Biomedicines. 2024 May 17;12(5):1112. doi: 10.3390/biomedicines12051112 (PMC11117922; doi:10.3390/biomedicines12051112)
Supplement: Supplementary file 1 [file biomedicines-12-01112-s001.zip › biomedicines-2972903-SM final/Supplementary files/Supplementary Figure S1.pdf]

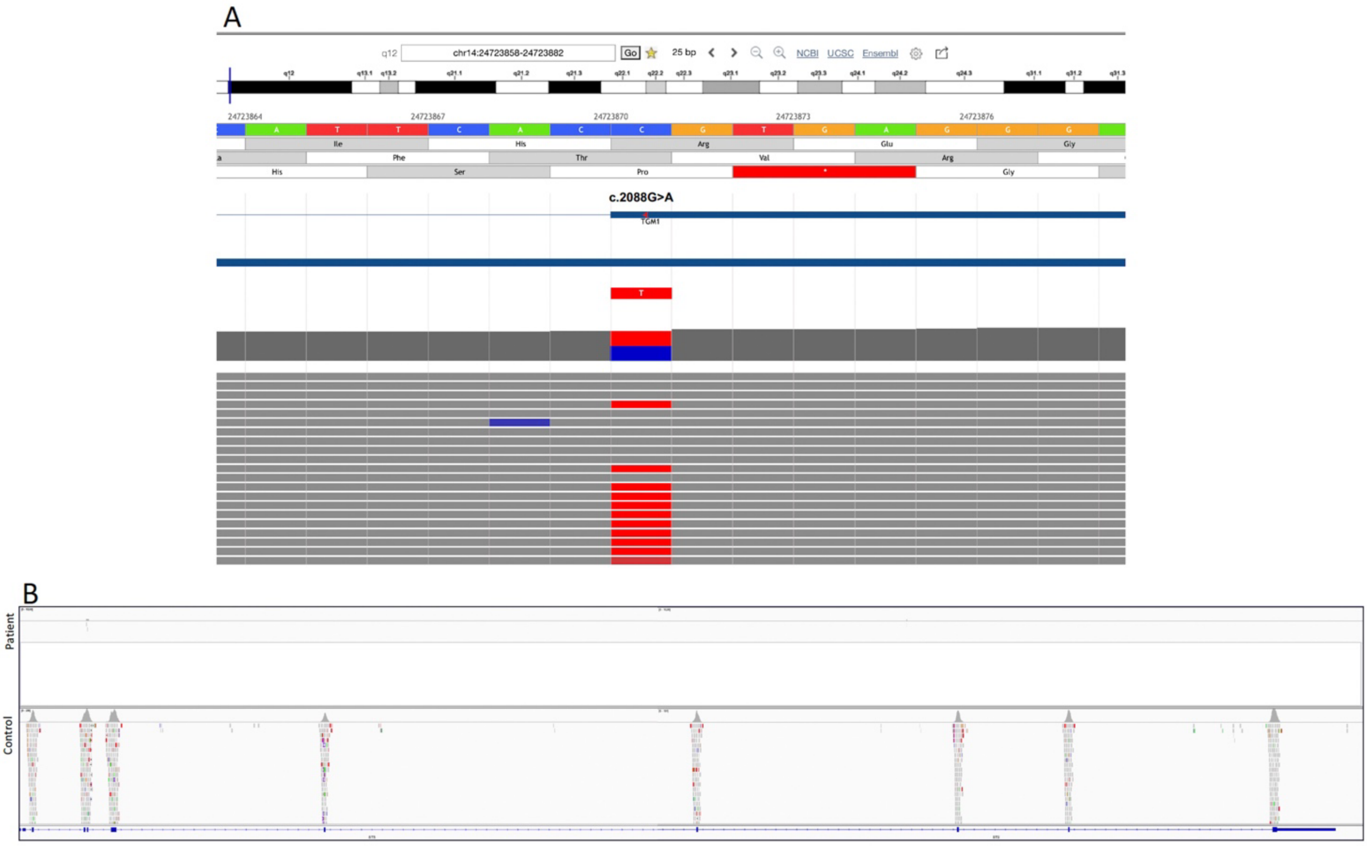

Figure S1

Integrative Genomics Viewer visualization of NGS data. (A) Position of the synonymous variant c.2088G>A p.(Thr696=) in the TGM1 exon 13 sequence; (B) Coverage of the 9 exons of STS gene (NM_ 000351.7) in a patient hemizygous for a whole gene deletion, compared with a normal male. In the patient, there are no visible reads for any exons of the STS gene.
