# Supplementary material for: Comprehensive Molecular Analysis of Disease-Related Genes as First-Tier Test for Early Diagnosis, Classification, and Management of Patients Affected by Nonsyndromic Ichthyosis
Source: Biomedicines. 2024 May 17;12(5):1112. doi: 10.3390/biomedicines12051112 (PMC11117922; doi:10.3390/biomedicines12051112)
Supplement: Supplementary file 1 [file biomedicines-12-01112-s001.zip › biomedicines-2972903-SM final/Supplementary files/SUPPLEMENTARY Table S1.pdf]

Supplementary Table S1 - ACGM pathogenic criteria for small variant (SV) classification and their relative Combined Annotation Dependent Depletion (CADD) scores

			ACGM CLASSIFICATION		ACGM PATHOGENICITY CRITERIA										CADD Score
ALOX12B	c.47C>T	p.(Ser16Leu)	pathogenic	Class 5	PP1	PP2		PP5		PM2	PM3	PM5			23.8
	c.1798C>T	p.(Arg600Trp)	likely pathogenic	Class 4		PP2			PM1	PM2	PM3	PM5			20.0
	c.1907G>T	p.(Ser636Ile)§	likely pathogenic	Class 4		PP2	PP3			PM2	PM3				26.8
	c.1192C>T	p.(His398Tyr)	pathogenic	Class 5		PP2	PP3	PP5	PM1	PM2	PM3				25.5
	c.1259G>A	p.(Cys420Tyr)§	likely pathogenic	Class 4		PP2			PM1	PM2	PM3	PM5			27.1
	c.1642C>T	p.(Arg548Trp)	pathogenic	Class 5		PP2		PP5	PM1	PM2	PM3				26.6
	c.1909C>G	p.(Arg637Gly)§	VUS	Class 3		PP2				PM2	PM3				**15.28**
	EX3_15del	?	pathogenic	Class 5				PP5		PM2	PM3		PVS1		30.0
NIPAL4	c.398C>A	p.(Pro133His)	likely pathogenic	Class 4			PP3			PM2	PM3				32.0
	c.514_515dup	p.(Met172Ilefs*)§	pathogenic	Class 5						PM2	PM3		PVS1		
	c.137G>A	p.(Trp46*)§	pathogenic	Class 5						PM2	PM3		PVS1		
TGM1	c.1166G>A	p.(Arg389His)	pathogenic	Class 5			PP3	PP5	PM1	PM2	PM3	PM5			33.0
	c.2088G>A	p.(Thr696=)§	likely pathogenic	Class 4						PM2	PM3			PS3	**17.8**

The CADD-SV scores range from 0 (potentially benign) to 48 (potentially pathogenic), indicating the position of the novel variant within the gnomAD-SV score distribution.
CADD score ondicates the position of the novel variant within the gnomAD-SV score distribution.
A CADD score above 20 corresponds to the top top 1%, and 30 to the top 0.1% of scores observed from gnomAD-SV.
§ Novel causative variants identified in this study

Table S1. Overall number of rare variants identified in the ichthyosis-related genes of 300 unaffected individuals

Subject ID	GENE	GenBank #	Nucleotide variant	gene position	Exon	Presumed effect	Presumed protein variant	gnomAD frequency	ACMG prediction	OMIM identification
106	ABCA12	NM_173076.3	c.300T>G	exonic	3	nonsynonymous	p.Asp100Glu	6/276566=0	VOUS	
74	ABCA12	NM_173076.3	c.346G>T	exonic	4	nonsynonymous	p.Asp116Tyr	7/245398=0	VOUS	
108	ABCA12	NM_173076.3	c.485C>T	exonic	5	nonsynonymous	p.Ala162Val	384/276512=0.001	VOUS	
146	ABCA12	NM_173076.3	c.485C>T	exonic	5	nonsynonymous	p.Ala162Val	384/276512=0.001	VOUS	
188	ABCA12	NM_173076.3	c.485C>T	exonic	5	nonsynonymous	p.Ala162Val	384/276512=0.001	VOUS	
191	ABCA12	NM_173076.3	c.501G>C	exonic	5	nonsynonymous	p.Leu167Phe		VOUS	
195	ABCA12	NM_173076.3	c.485C>T	exonic	5	nonsynonymous	p.Ala162Val	384/276512=0.001	VOUS	
184	ABCA12	NM_173076.3	c.539T>C	exonic	6	nonsynonymous	p.Ile180Thr	3/245112=0	VOUS	
200	ABCA12	NM_173076.3	c.1141G>C	exonic	10	nonsynonymous	p.Val381Leup.V381L	239/277242=0.001	LB	
89	ABCA12	NM_173076.3	c.1222T>C	exonic	11	nonsynonymous	p.Ser408Pro	322/276876=0.001	B	
77	ABCA12	NM_173076.3	c.1222T>C	exonic	11	nonsynonymous	p.Ser408Pro	322/276876=0.001	B	
89	ABCA12	NM_173076.3	c.1475A>G	exonic	12	nonsynonymous	p.Asn492Ser	5/276916=0	VOUS	
141	ABCA12	NM_173076.3	c.1446A>C	exonic	12	nonsynonymous	p.Glu482Asp	29/276918=0	VOUS	
114	ABCA12	NM_173076.3	c.1743C>G	exonic	14	nonsynonymous	p.Asp581Glu	246/277138=0.001	LB	
15	ABCA12	NM_173076.3	c.1816G>A	exonic	15	nonsynonymous	p.Asp606Asn	2/245978=0	VOUS	# 242500 ICHTHYOSIS, CONGENITAL, AUTOSOMAL RECESSIVE 4B; ARCI4B
7	ABCA12	NM_173076.3	c.2129A>G	exonic	17	nonsynonymous	p.Tyr710Cys	1/246028=0	VOUS	
14	ABCA12	NM_173076.3	c.2243G>A	exonic	17	nonsynonymous	p.Arg748Lys	6/245968=0	VOUS	
5	ABCA12	NM_173076.3	c.3098T>C	exonic	22	nonsynonymous	p.Ile1033Thr	2/245894=0	VOUS	
55	ABCA12	NM_173076.3	c.3481A>T	exonic	24	nonsynonymous	p.Met1161Leu	363/277160=0.001	LB	
62	ABCA12	NM_173076.3	c.3481A>T	exonic	24	nonsynonymous	p.Met1161Leu	363/277160=0.001	LB	
54	ABCA12	NM_173076.3	c.3481A>T	exonic	24	nonsynonymous	p.Met1161Leu	363/277160=0.001	LB	
49	ABCA12	NM_173076.3	c.4618G>T	exonic	31	nonsynonymous	p.Ala1540Ser	9/245910=0	VOUS	
41	ABCA12	NM_173076.3	c.5051T>C	exonic	33	nonsynonymous	p.Ile1684Thr	4/245586=0	VOUS	
36	ABCA12	NM_173076.3	c.5617G>A	exonic	37	nonsynonymous	p.Val1873Ile	670/276578=0.002	LB	
29	ABCA12	NM_173076.3	c.6208G>A	exonic	42	nonsynonymous	p.Val2070Ile	514/276952=0.002	B	
172	ABCA12	NM_173076.3	c.6704A>C	exonic	45	nonsynonymous	p.Glu2235Ala	184/277156=0.001	B	
158	ABCA12	NM_173076.3	c.6919A>G	exonic	46	nonsynonymous	p.Ile2307Val	486/277078=0.002	LB	
168	ABCA12	NM_173076.3	c.6919A>G	exonic	46	nonsynonymous	p.Ile2307Val	486/277078=0.002	LB	
160	ABCA12	NM_173076.3	c.6919A>G	exonic	46	nonsynonymous	p.Ile2307Val	486/277078=0.002	LB	
161	ABCA12	NM_173076.3	c.6919A>G	exonic	46	nonsynonymous	p.Ile2307Val	486/277078=0.002	LB	
65	ABCA12	NM_173076.3	c.7631C>T	exonic	52	nonsynonymous	p.Thr2544Ile	379/276596=0.001	B	
74	ABHD5	NM_001365649.1	c.22A>G	exonic	3	nonsynonymous	p.Thr8Ala	12/237020=0	B	
66	ABHD5	NM_001365649.1	c.22A>G	exonic	3	nonsynonymous	p.Thr8Ala	12/237020=0	B	# 275630 CHANARIN-DORFMAN SYNDROME; CDS AR
135	ABHD5	NM_001365649.1	c.505C>G	exonic	4	nonsynonymous	p.Pro169Ala	21/277218=0	VOUS	
96	ABHD5	NM_001365649.1	c.883G>T	exonic	7	stopgain	p.Glu295*	1/121404=0	P	
47	ALDH3A2	NM_001031806.2	c.17G>C	exonic	1	nonsynonymous	p.Arg6Pro	3/223156=0	VOUS	
89	ALDH3A2	NM_001031806.2	c.119A>G	exonic	1	nonsynonymous	p.Asp40Gly	155/218008=0.001	LB	
116	ALDH3A2	NM_001031806.2	c.17G>C	exonic	1	nonsynonymous	p.Arg6Pro	3/223156=0	VOUS	
148	ALDH3A2	NM_001031806.2	c.28C>G	exonic	1	nonsynonymous	p.Gln10Glu	806/218946=0.004	B	# 270200 SJOGREN-LARSSON SYNDROME; SLS<br>Alternative titles; symbols ICHTHYOSIS, SPASTIC NEUROLOGIC DISORDER, AND OLIGOPHRENIA AR
55	ALDH3A2	NM_001031806.2	c.17G>C	exonic	2	nonsynonymous	p.Arg6Pro	3/223156=0	VOUS	
76	ALDH3A2	NM_001031806.2	c.661G>A	exonic	4	nonsynonymous	p.Asp221Asn	1/244348=0	VOUS	
204	ALDH3A2	NM_001031806.1	c.1270C>T	exonic	8	nonsynonymous	p.Pro424Ser	1205/277210=0.004	VOUS	
40	ALDH3A2	NM_001031806.2	c.1270C>T	exonic	9	nonsynonymous	p.Pro424Ser	1205/277210=0.004	VOUS	
118	ALDH3A2	NM_001031806.2	c.1270C>T	exonic	9	nonsynonymous	p.Pro424Ser	1205/277210=0.004	VOUS	
143	ALDH3A2	NM_001031806.2	c.1270C>T	exonic	9	nonsynonymous	p.Pro424Ser	1205/277210=0.004	VOUS	
124	ALOX12B	NM_001139.3	c.280G>A	exonic	2	nonsynonymous	p.Gly94Ser	2588/264690	LB	
26	ALOX12B	NM_001139.3	c.380C>T	exonic	3	nonsynonymous	p.Pro127Leu	66/276792=0	LB	
180	ALOX12B	NM_001139.3	c.526G>A	exonic	4	nonsynonymous	p.Glu176Lys	92/276916=0	VOUS	
181	ALOX12B	NM_001139.3	c.526G>A	exonic	4	nonsynonymous	p.Glu176Lys	92/276916=0	VOUS	
68	ALOX12B	NM_001139.3	c.556A>T	exonic	5	nonsynonymous	p.Ile186Phe	0	VOUS	#242100 ICHTHYOSIS, CONGENITAL, AUTOSOMAL RECESSIVE
172	ALOX12B	NM_001139.3	c.715A>G	exonic	6	nonsynonymous	p.Ile239Val	24/277224=0	VOUS	
80	ALOX12B	NM_001139.3	c.1156C>T	exonic	9	nonsynonymous	p.Arg386Cys	9/276770=0	P	
158	ALOX12B	NM_001139.3	c.1431delC	exonic	11	frameshift	p.Asp477Glufs*37	1/245856=0	P	
93	ALOX12B	NM_001139.3	c.1565C>T	exonic	12	nonsynonymous	p.Pro522Leu	277/277210=0.001	VOUS	
4	ALOXE3	NM_001165960.1	c.62C>T	exonic	1	nonsynonymous	p.Pro21Leu	16/168304=0	VOUS	

13	ALOXE3	NM_001165960.1	c.30G>T	exonic	1	nonsynonymous	p.Leu10Phe		VOUS	# 606545 ICHTHYOSIS, CONGENITAL, AUTOSOMAL RECESSIVE 3; ARCI3
45	ALOXE3	NM_001165960.1	c.280C>G	exonic	2	nonsynonymous	p.Pro94Ala	143/182810=0.001	VOUS	
95	ALOXE3	NM_001165960.1	c.809G>A	exonic	4	nonsynonymous	p.Arg270Gln	7/246260=0	VOUS	
138	ALOXE3	NM_001165960.1	c.989T>C	exonic	6	nonsynonymous	p.Ile330Thr	4/246268=0	VOUS	
137	ALOXE3	NM_001165960.1	c.1076C>T	exonic	6	nonsynonymous	p.Ala359Val	51/246268=0	VOUS	
163	ALOXE3	NM_001165960.1	c.1483C>T	exonic	9	nonsynonymous	p.Pro495Ser		VOUS	
175	ALOXE3	NM_001165960.1	c.1454T>A	exonic	9	nonsynonymous	p.Leu485Gln		LP	
180	ALOXE3	NM_001165960.1	c.1843C>T	exonic	12	nonsynonymous	p.His615Tyr		LP	
190	ALOXE3	NM_001165960.1	c.2102C>A	exonic	14	nonsynonymous	p.Thr701Asn	102/276992=0	VOUS	
190	ALOXE3	NM_001165960.1	c.2404C>T	exonic	16	nonsynonymous	p.Arg802Trp	239/277182=0.001	VOUS	
193	ALOXE3	NM_001165960.1	c.2510T>C	exonic	16	nonsynonymous	p.Ile837Thr	333/277202=0.001	LB	
194	ALOXE3	NM_001165960.1	c.2510T>C	exonic	16	nonsynonymous	p.Ile837Thr	333/277202=0.001	VOUS	
199	ALOXE3	NM_001165960.1	c.2510T>C	exonic	16	nonsynonymous	p.Ile837Thr	333/277202=0.001	VOUS	
201	ALOXE3	NM_001165960.1	c.2510T>C	exonic	16	nonsynonymous	p.Ile837Thr	333/277202=0.001	VOUS	
3	CAST	NM_001750.7	c.200C>T	exonic	3	nonsynonymous	p.Ser67Leu	12/276074=0	VOUS	# 616295 PEELING SKIN WITH LEUKONYCHIA, ACRAL PUNCTATE KERATOSES, CHEILITIS, AND KNUCKLE PADS; PLACK AD
149	CAST	NM_001750.7	c.620C>T	exonic	9	nonsynonymous	p.Pro207Leu	5/246206=0	VOUS	
98	CAST	NM_001750.7	c.775A>G	exonic	11	nonsynonymous	p.Thr259Ala	943/276512=0.003	B	
72	CAST	NM_001750.7	c.986C>G	exonic	14	nonsynonymous	p.Ala329Gly	74/276982=0	VOUS	
98	CAST	NM_001750.7	c.925A>C	exonic	14	nonsynonymous	p.Ile309Leu	2994/276670=0.011	B	
150	CAST	NM_001750.7	c.1177C>T	exonic	16	nonsynonymous	p.Arg393Cys	34/276754=0	LB	
9	CAST	NM_001750.7	c.1207G>C	exonic	17	nonsynonymous	p.Ala403Pro	1334/276854=0.005	B	
194	CAST	NM_001750.7	c.1283C>T	exonic	17	nonsynonymous	p.Thr428Met	909/277064=0.003	B	
12	CAST	NM_001750.7	c.1835A>G	exonic	25	nonsynonymous	p.Lys612Arg	585/265502=0.002	B	
45	CAST	NM_001750.7	c.1835A>G	exonic	25	nonsynonymous	p.Lys612Arg	585/265502=0.002	B	
16	CDSN	NM_001264.4	c.32G>A	exonic	1	nonsynonymous	p.Arg11His	790/239488=0.003	B	# 146520 HYPOTRICHOSIS 2; HYPT2 AD # 270300 PEELING SKIN SYNDROME 1; PSS1 AR
98	CDSN	NM_001264.4	c.32G>A	exonic	1	nonsynonymous	p.Arg11His	790/239488=0.003	B	
37	CDSN	NM_001264.4	c.475A>G	exonic	2	nonsynonymous	p.Ser159Gly	47/276174=0	VOUS	
60	CDSN	NM_001264.4	c.475A>G	exonic	2	nonsynonymous	p.Ser159Gly	47/276174=0	VOUS	
77	CDSN	NM_001264.4	c.1302C>A	exonic	2	nonsynonymous	p.Ser434Arg	916/276996=0.003	B	
127	CDSN	NM_001264.4	c.782G>T	exonic	2	nonsynonymous	p.Gly261Val		VOUS	
4	CERS3	NM_001290341.2	c.233C>T	exonic	6	nonsynonymous	p.Ser78Leu		VOUS	# 615023 ICHTHYOSIS, CONGENITAL, AUTOSOMAL RECESSIVE 9; ARCI9
72	CERS3	NM_001290341.2	c.914A>G	exonic	13	nonsynonymous	p.His305Arg	3292/264968=0.012	VOUS	
86	CERS3	NM_001290341.2	c.914A>G	exonic	13	nonsynonymous	p.His305Arg	3292/264968=0.012	VOUS	
193	CERS3	NM_001290341.2	c.914A>G	exonic	13	nonsynonymous	p.His305Arg	3292/264968=0.012	B	
23	CERS3	NM_001290341.2	c.1151G>A	exonic	14	nonsynonymous	p.Arg384Lys	481/277162=0.002	B	
181	CLDN1	NM_021101.5	c.136A>T	exonic	1	nonsynonymous	p.Met46Leu	15/246242=0	VOUS	# 607626 ICHTHYOSIS, LEUKOCYTE VACUOLES, ALOPECIA, AND SCLEROSING CHOLANGITIS; ILVASC AR
182	CLDN1	NM_021101.5	c.136A>T	exonic	1	nonsynonymous	p.Met46Leu	15/246242=0	VOUS	
144	CLDN1	NM_021101.5	c.278T>C	exonic	2	nonsynonymous	p.Ile93Thr		VOUS	
31	CLDN1	NM_021101.5	c.631G>A	exonic	4	nonsynonymous	p.Val211Met	30/277152=0	VOUS	
81	CYP4F22	NM_173483.4	c.109C>T	exonic	3	nonsynonymous	p.Arg37Cys	32/121338=0	VOUS	# 604777 ICHTHYOSIS, CONGENITAL, AUTOSOMAL RECESSIVE 5; ARCI5 Alternative titles; symbols ICHTHYOSIS, NONLAMELLAR AND NONERYTHRODERMIC, CONGENITAL, AUTOSOMAL RECESSIVE; NNCI
94	CYP4F22	NM_173483.4	c.68C>T	exonic	3	nonsynonymous	p.Ala23Val	45/277126=0	VOUS	
52	CYP4F22	NM_173483.4	c.485C>G	exonic	6	nonsynonymous	p.Ala162Gly	47/277122=0	VOUS	
190	CYP4F22	NM_173483.4	c.463C>T	exonic	6	nonsynonymous	p.His155Tyr	121/277112=0	VOUS	
194	CYP4F22	NM_173483.4	c.463C>T	exonic	6	nonsynonymous	p.His155Tyr	121/277112=0	VOUS	
148	CYP4F22	NM_173483.4	c.665G>T	exonic	7	nonsynonymous	p.Cys222Phe		VOUS	
64	CYP4F22	NM_173483.4	c.712G>A	exonic	8	nonsynonymous	p.Ala238Thr		LB	
199	CYP4F22	NM_173483.4	c.851G>A	exonic	8	nonsynonymous	p.Arg284Gln	9/276864=0	VOUS	
59	CYP4F22	NM_173483.4	c.1148C>T	exonic	11	nonsynonymous	p.Thr383Ile		VOUS	
67	ELOVL4	NM_022726.4	c.243C>G	exonic	2	nonsynonymous	p.Ile81Met		VOUS	# 614457 ICHTHYOSIS, SPASTIC QUADRIPLEGIA, AND IMPAIRED INTELLECTUAL DEVELOPMENT; ISQMR
61	ELOVL4	NM_022726.4	c.800T>C	exonic	6	nonsynonymous	p.Ile267Thr	2004/276984=0.007	LB	
127	ELOVL4	NM_022726.4	c.800T>C	exonic	6	nonsynonymous	p.Ile267Thr	2004/276984=0.007	LB	
172	ELOVL4	NM_022726.4	c.800T>C	exonic	6	nonsynonymous	p.Ile267Thr	2004/276984=0.007	LB	
204	ELOVL4	NM_022726.4	c.814G>C	exonic	6	nonsynonymous	p.Glu272Gln	2735/277052=0.01	LB	
25	GJA1	NM_000165.5	c.1108C>T	exonic	2	nonsynonymous	p.Arg370Cys	3/245356=0	VOUS	
32	GJA1	NM_000165.5	c.1109G>A	exonic	2	nonsynonymous	p.Arg370His	1/245340=0	VOUS	
156	GJA1	NM_000165.5	c.157C>T	exonic	2	nonsynonymous	p.Arg53Cys	2/246262=0	LP	
63	GJA1	NM_000165.5	c.758C>T	exonic	2	nonsynonymous	p.Ala253Val	2227/277148=0.008	LB	
141	GJA1	NM_000165.5	c.758C>T	exonic	2	nonsynonymous	p.Ala253Val	2227/277148=0.008	LB	

156	GJA1	NM_000165.5	c.758C>T	exonic	2	nonsynonymous	p.Ala253Val	2227/277148=0.008	LB	
171	GJA1	NM_000165.5	c.758C>T	exonic	2	nonsynonymous	p.Ala253Val	2227/277148=0.008	LB	
154	GJB2	NM_004004.6	c.296G>A	exonic	2	nonsynonymous	p.Arg99Lys		VOUS	# 148210 KERATITIS-ICHTHYOSIS-DEAFNESS SYNDROME, AUTOSOMAL DOMINANT; KIDAD AD
145	GJB2	NM_004004.6	c.358_360delGAG	exonic	2	inframe	p.Glu120del	20/275990=0	P	
166	GJB2	NM_004004.6	c.358_360delGAG	exonic	2	inframe	p.Glu120del	20/275990=0	P	
6	GJB2	NM_004004.6	c.35delG	exonic	2	frameshift	p.Gly12Valfs*2	1721/275002=0.006	P	
35	GJB2	NM_004004.6	c.35delG	exonic	2	frameshift	p.Gly12Valfs*2	1721/275002=0.006	P	
71	GJB2	NM_004004.6	c.35delG	exonic	2	frameshift	p.Gly12Valfs*2	1721/275002=0.006	P	
84	GJB2	NM_004004.6	c.35delG	exonic	2	frameshift	p.Gly12Valfs*2	1721/275002=0.006	P	
103	GJB2	NM_004004.6	c.35delG	exonic	2	frameshift	p.Gly12Valfs*2	1721/275002=0.006	P	
117	GJB2	NM_004004.6	c.35delG	exonic	2	frameshift	p.Gly12Valfs*2	1721/275002=0.006	P	
151	GJB2	NM_004004.6	c.35delG	exonic	2	frameshift	p.Gly12Valfs*2	1721/275002=0.006	P	
185	GJB2	NM_004004.6	c.35delG	exonic	2	frameshift	p.Gly12Valfs*2	1721/275002=0.006	P	
198	GJB2	NM_004004.6	c.35delG	exonic	2	frameshift	p.Gly12Valfs*2	1721/275002=0.006	P	
202	GJB2	NM_004004.6	c.35delG	exonic	2	frameshift	p.Gly12Valfs*2	1721/275002=0.006	P	
204	GJB2	NM_004004.6	c.35delG	exonic	2	frameshift	p.Gly12Valfs*2	1721/275002=0.006	P	
205	GJB2	NM_004004.6	c.35delG	exonic	2	frameshift	p.Gly12Valfs*2	1721/275002=0.006	P	
8	GJB2	NM_004004.6	c.88A>G	exonic	2	nonsynonymous	p.Ile30Val		LP	
36	GJB2	NM_004004.6	c.269T>C	exonic	2	nonsynonymous	p.Leu90Pro	177/277032=0.001	P	
12	GJB2	NM_004004.6	c.101T>C	exonic	2	nonsynonymous	p.Met34Thr	2487/276420=0.009	P	
17	GJB2	NM_004004.6	c.101T>C	exonic	2	nonsynonymous	p.Met34Thr	2487/276420=0.009	P	
70	GJB2	NM_004004.6	c.101T>C	exonic	2	nonsynonymous	p.Met34Thr	2487/276420=0.009	P	
88	GJB2	NM_004004.6	c.101T>C	exonic	2	nonsynonymous	p.Met34Thr	2487/276420=0.009	P	
159	GJB2	NM_004004.6	c.101T>C	exonic	2	nonsynonymous	p.Met34Thr	2487/276420=0.009	P	
184	GJB2	NM_004004.6	c.101T>C	exonic	2	nonsynonymous	p.Met34Thr	2487/276420=0.009	P	
129	GJB2	NM_004004.6	c.23C>T	exonic	2	nonsynonymous	p.Thr8Met	21/275096=0	P	
20	GJB2	NM_004004.6	c.457G>A	exonic	2	nonsynonymous	p.Val153Ile	2433/276862=0.009	B	
42	GJB2	NM_004004.6	c.457G>A	exonic	2	nonsynonymous	p.Val153Ile	2433/276862=0.009	B	
77	GJB2	NM_004004.6	c.457G>A	exonic	2	nonsynonymous	p.Val153Ile	2433/276862=0.009	B	
79	GJB2	NM_004004.6	c.457G>A	exonic	2	nonsynonymous	p.Val153Ile	2433/276862=0.009	B	
140	GJB2	NM_004004.6	c.457G>A	exonic	2	nonsynonymous	p.Val153Ile	2433/276862=0.009	B	
153	GJB2	NM_004004.6	c.467T>A	exonic	2	nonsynonymous	p.Val156Asp		VOUS	
22	GJB2	NM_004004.6	c.109G>A	exonic	2	nonsynonymous	p.Val37Ile	2011/276450=0.007	P	
54	GJB3	NM_024009.3	c.196_198delGAC	exonic	2	inframe	p.Asp66del	38/277154=0	VOUS	# 133200 ERYTHROKERATODERMIA VARIABILIS ET PROGRESSIVA 1; EKVP1 AD
119	GJB3	NM_024009.3	c.196_198delGAC	exonic	2	inframe	p.Asp66del	38/277154=0	VOUS	
150	GJB3	NM_024009.3	c.293G>A	exonic	2	nonsynonymous	p.Arg98His	23/276870=0	VOUS	
166	GJB3	NM_024009.3	c.293G>A	exonic	2	nonsynonymous	p.Arg98His	23/276870=0	VOUS	
24	GJB3	NM_024009.3	c.316C>T	exonic	2	nonsynonymous	p.Arg106Cys	40/276618=0	VOUS	
7	GJB3	NM_024009.3	c.422T>C	exonic	2	nonsynonymous	p.Ile141Thr		VOUS	
9	GJB3	NM_024009.3	c.529T>G	exonic	2	nonsynonymous	p.Tyr177Asp	525/276788=0.002	LB	
196	GJB3	NM_024009.3	c.659A>T	exonic	2	nonsynonymous	p.Lys220Met	1/246042=0	VOUS	
168	GJB3	NM_024009.3	c.670C>T	exonic	2	stopgain	p.Arg224*	11/276538=0	P	
3	GJB4	NM_153212.3	c.119C>T	exonic	2	nonsynonymous	p.Ala40Val	13/277082=0	VOUS	# 617524 ERYTHROKERATODERMIA VARIABILIS ET PROGRESSIVA 2; AD
46	GJB4	NM_153212.3	c.478C>T	exonic	2	nonsynonymous	p.Arg160Cys	20/276732=0	VOUS	
180	GJB4	NM_153212.3	c.314A>G	exonic	2	nonsynonymous	p.His105Arg	10/277130=0	VOUS	
101	GJB4	NM_153212.3	c.153delT	exonic	2	frameshift	p.Phe51Leufs*57	1464/276392=0.005	P	
137	GJB4	NM_153212.3	c.153delT	exonic	2	frameshift	p.Phe51Leufs*57	1464/276392=0.005	P	
160	GJB4	NM_153212.3	c.153delT	exonic	2	frameshift	p.Phe51Leufs*57	1464/276392=0.005	P	
34	GJB4	NM_153212.3	c.770C>T	exonic	2	nonsynonymous	p.Ser257Leu	20/275366=0	VOUS	
201	GJB4	NM_153212.3	c.389C>T	exonic	2	nonsynonymous	p.Thr130Met	30/277058=0	VOUS	
19	GJB4	NM_153212.3	c.254C>T	exonic	2	nonsynonymous	p.Thr85Met	8/245960=0	LP	
86	GJB4	NM_153212.3	c.254C>T	exonic	2	nonsynonymous	p.Thr85Met	8/245960=0	LP	
103	GJB4	NM_153212.3	c.384G>A	exonic	2	stopgain	p.Trp128*	527/277132=0.002	P	
151	GJB4	NM_153212.3	c.384G>A	exonic	2	stopgain	p.Trp128*	527/277132=0.002	P	
136	GJB4	NM_153212.3	c.386G>A	exonic	2	stopgain	p.Trp129*	59/277112=0	P	
130	GJB6	NM_001370092.1	c.212T>C	exonic	5	nonsynonymous	p.Val71Ala	110/277050=0	VOUS	# 129500 ECTODERMAL DYSPLASIA 2, CLOUSTON TYPE; ECTD2 AD
167	GJB6	NM_001370092.1	c.607A>G	exonic	5	nonsynonymous	p.Met203Val	247/277132=0.001	LB	
25	GJB6	NM_001370092.1	c.688A>T	exonic	5	nonsynonymous	p.Asn230Tyr		VOUS	

11	KRT1	NM_006121.4	c.860T>C	exonic	3	nonsynonymous	p.Ile287Thr	1/246176=0	VOUS	# 113800 EPIDERMOLYTIC HYPERKERATOSIS 1; EHK1 # 600962 PALMOPLANTAR KERATODERMA, NONEPIDERMOLYTIC; NEPPK
34	KRT1	NM_006121.4	c.982A>T	exonic	5	nonsynonymous	p.Thr328Ser	332/277126=0.001	LB	
10	KRT1	NM_006121.4	c.1294C>T	exonic	7	nonsynonymous	p.Arg432Cys	143/277212=0.001	B	
157	KRT1	NM_006121.4	c.1390G>A	exonic	7	nonsynonymous	p.Asp464Asn	1/246258=0	VOUS	
178	KRT1	NM_006121.4	c.1693A>G	exonic	9	nonsynonymous	p.Ser565Gly		VOUS	
77	KRT1	NM_006121.4	c.1894G>A	exonic	9	nonsynonymous	p.Val632Met	2/245184=0	VOUS	
131	KRT1	NM_006121.4	c.1912A>G	exonic	9	nonsynonymous	p.Thr638Ala	35/276622=0	LB	
21	KRT10	NM_001379366.1	c.257G>A	exonic	1	nonsynonymous	p.Arg86His	397/264258=0.002	VOUS	# 609165 ICHTHYOSIS WITH CONFETTI; IWC AD # 113800 EPIDERMOLYTIC HYPERKERATOSIS; EHK AD-AR
53	KRT10	NM_001379366.1	c.98C>T	exonic	1	nonsynonymous	p.Ser33Phe	72/274952=0	VOUS	
110	KRT10	NM_001379366.1	c.71G>A	exonic	1	nonsynonymous	p.Gly24Glu	34/179406=0	VOUS	
115	KRT10	NM_001379366.1	c.158G>A	exonic	1	nonsynonymous	p.Ser53Asn	65/276636=0	VOUS	
137	KRT10	NM_001379366.1	c.710+6T>C	intronic	2			5/277170=0	VOUS	
12	KRT10	NM_001379366.1	c.1495T>C	exonic	7	nonsynonymous	p.Tyr499His	79/97856=0.001	VOUS	
23	KRT10	NM_001379366.1	c.1524C>G	exonic	7	nonsynonymous	p.Ser508Arg	153/101426=0.002	VOUS	
88	KRT10	NM_001379366.1	c.1471_1479delCACGGCGGC	exonic	7	inframe	p.His491_Gly493del		VOUS	
124	KRT10	NM_001379366.1	c.1443_1457delAAGCTCCGGCGGCGG	exonic	7	inframe	p.Ser482_Gly486del	1/99466=0	VOUS	
144	KRT10	NM_001379366.1	c.1650_1667delCAGCAGCTCCGGCGGCGG	exonic	7	inframe	p.Ser551_Gly556del	34/219570=0	VOUS	
22	KRT2	NM_000423.3	c.146G>A	exonic	1	nonsynonymous	p.Gly49Asp	3/242214=0	VOUS	# 146800 ICHTHYOSIS BULLOSA OF SIEMENS; IBS AD
139	KRT2	NM_000423.3	c.317G>A	exonic	1	nonsynonymous	p.Ser106Asn	641/254108=0.003	B	
160	KRT2	NM_000423.3	c.767A>G	exonic	2	nonsynonymous	p.Asn256Ser	110/277226=0	LB	
192	KRT2	NM_000423.3	c.767A>G	exonic	2	nonsynonymous	p.Asn256Ser	110/277226=0	LB	
27	KRT2	NM_000423.3	c.1550C>G	exonic	9	nonsynonymous	p.Ala517Gly	993/277108=0.004	LB	
33	KRT2	NM_000423.3	c.1550C>G	exonic	9	nonsynonymous	p.Ala517Gly	993/277108=0.004	LB	
152	KRT2	NM_000423.3	c.1750A>G	exonic	9	nonsynonymous	p.Ile584Val		VOUS	
38	KRT9	NM_000226.4	c.245G>A	exonic	1	nonsynonymous	p.Ser82Asn	628/274158=0.002	VOUS	# 144200 PALMOPLANTAR KERATODERMA, EPIDERMOLYTIC; EPPK AD
73	KRT9	NM_000226.4	c.245G>A	exonic	1	nonsynonymous	p.Ser82Asn	628/274158=0.002	VOUS	
122	KRT9	NM_000226.4	c.245G>A	exonic	1	nonsynonymous	p.Ser82Asn	628/274158=0.002	VOUS	
39	KRT9	NM_000226.4	c.245G>A	exonic	1	nonsynonymous	p.Ser82Asn	628/274158=0.002	VOUS	
168	KRT9	NM_000226.4	c.49G>A	exonic	1	nonsynonymous	p.Gly17Ser		VOUS	
64	KRT9	NM_000226.4	c.1049C>G	exonic	5	nonsynonymous	p.Thr350Ser		VOUS	
101	KRT9	NM_000226.4	c.1630G>A	exonic	7	nonsynonymous	p.Gly544Arg	10/179160=0	VOUS	
50	LIPN	NM_001102469.1	c.326A>C	exonic	3	nonsynonymous	p.Asp109Ala	2/245360=0	VOUS	# 613943 ICHTHYOSIS, CONGENITAL, AUTOSOMAL RECESSIVE 8; ARCI8
151	LIPN	NM_001102469.1	c.302delG	exonic	3	frameshift	p.Gly101Glufs*7	50/276298=0	P	
183	LIPN	NM_001102469.1	c.633T>G	exonic	5	nonsynonymous	p.Ile211Met	12/275930=0	VOUS	
9	LIPN	NM_001102469.1	c.754C>T	exonic	6	nonsynonymous	p.Leu252Phe	872/201394=0.004	B	
83	LIPN	NM_001102469.1	c.772G>A	exonic	6	nonsynonymous	p.Glu258Lys	316/215004=0.001	LB	
171	LIPN	NM_001102469.1	c.772G>A	exonic	6	nonsynonymous	p.Glu258Lys	316/215004=0.001	LB	
173	LIPN	NM_001102469.1	c.772G>A	exonic	6	nonsynonymous	p.Glu258Lys	316/215004=0.001	LB	
87	LIPN	NM_001102469.1	c.934G>T	exonic	8	nonsynonymous	p.Asp312Tyr	1/152140=0	VOUS	
2	NIPAL4	NM_001099287.1	c.86C>A	exonic	1	stopgain	p.Ser29*	3/121822=0	P	# 612281 ICHTHYOSIS, CONGENITAL, AUTOSOMAL RECESSIVE 6; ARCI6 AR
104	NIPAL4	NM_001099287.1	c.86C>A	exonic	1	stopgain	p.Ser29*	3/121822=0	P	
127	NIPAL4	NM_001099287.1	c.176C>A	exonic	1	nonsynonymous	p.Ala59Asp	0/242706=0	VOUS	
197	NIPAL4	NM_001099287.1	c.176C>A	exonic	1	nonsynonymous	p.Ala59Asp		VOUS	
58	NIPAL4	NM_001099287.1	c.238C>A	exonic	2	nonsynonymous	p.Leu80Ile		VOUS	
64	NIPAL4	NM_001099287.1	c.446C>T	exonic	2	nonsynonymous	p.Thr149Met	294/266230=0.001	VOUS	
120	NIPAL4	NM_001099287.1	c.446C>T	exonic	2	nonsynonymous	p.Thr149Met	294/266230=0.001	VOUS	
190	NIPAL4	NM_001099287.1	c.397G>A	exonic	2	nonsynonymous	p.Gly133Ser	9/277110=0	VOUS	
193	NIPAL4	NM_001099287.1	c.397G>A	exonic	2	nonsynonymous	p.Gly133Ser	9/277110=0	VOUS	
204	NIPAL4	NM_001099287.1	c.296T>C	exonic	2	nonsynonymous	p.Val99Ala	783/277214=0.003	B	
121	NIPAL4	NM_001099287.1	c.581C>T	exonic	4	nonsynonymous	p.Thr194Met	1/243716=0	VOUS	
179	NIPAL4	NM_001099287.1	c.730G>T	exonic	5	nonsynonymous	p.Val244Phe	170/254010=0.001	VOUS	
192	NIPAL4	NM_001099287.1	c.730G>T	exonic	5	nonsynonymous	p.Val244Phe	170/254010=0.001	VOUS	
1	NIPAL4	NM_001099287.1	c.1105G>A	exonic	6	nonsynonymous	p.Val369Ile	127/276938=0	VOUS	
56	NIPAL4	NM_001099287.1	c.965G>A	exonic	6	nonsynonymous	p.Arg322Gln	10/246210=0	LP	
141	NIPAL4	NM_001099287.1	c.839G>A	exonic	6	nonsynonymous	p.Arg280His	60/276956=0	VOUS	
51	PEX7	NM_000288.4	c.377A>C	exonic	4	nonsynonymous	p.Gln126Pr	1104/277164=0.004	VOUS	RHIZOMELIC CHONDRODYSPLASIA PUNCTATA, TYPE 1; RCDP1 PEROXISOME BIOGENESIS DISORDER 9; PBD9 AR
137	PEX7	NM_000288.4	c.961A>T	exonic	10	nonsynonymous	p.Ile321Phe		VOUS	
167	PHYH	NM_001323080.2	c.56C>T	exonic	4	nonsynonymous	p.Thr19Met	367/277256=0.001	VOUS	[illegible]

156	PHYH	NM_001323080.2	c.301C>G	exonic	6	nonsynonymous	p.Arg101Gly	351/277160=0.001	VOUS	# 266500 REFSUM DISEASE, CLASSIC
167	PHYH	NM_001323080.2	c.403G>A	exonic	7	nonsynonymous	p.Gly135Arg	2/246256=0	LP	
111	PNPLA1	NM_001374623.1	c.116C>G	exonic	1	nonsynonymous	p.Ala39Gly		VOUS	# 615024 ICHTHYOSIS, CONGENITAL, AUTOSOMAL RECESSIVE 10; ARCI10 AR
9	PNPLA1	NM_001374623.1	c.383C>T	exonic	2	nonsynonymous	p.Thr128Met	397/276794=0.001	VOUS	
125	PNPLA1	NM_001374623.1	c.472T>A	exonic	3	nonsynonymous	p.Cys158Ser		VOUS	
58	PNPLA1	NM_001374623.1	c.592G>T	exonic	4	nonsynonymous	p.Asp198Tyr		VOUS	
107	PNPLA1	NM_001374623.1	c.714+7G>A	intronic	4			2/244890=0	VOUS	
85	PNPLA1	NM_001374623.1	c.745G>A	exonic	5	nonsynonymous	p.Glu249Lys	803/138609=0.006	LB	
19	PNPLA1	NM_001374623.1	c.745G>A	exonic	5	nonsynonymous	p.Glu249Lys	813/277218=0.003	LB	
28	PNPLA1	NM_001374623.1	c.745G>A	exonic	5	nonsynonymous	p.Glu249Lys	813/277218=0.003	LB	
44	PNPLA1	NM_001374623.1	c.745G>A	exonic	5	nonsynonymous	p.Glu249Lys	813/277218=0.003	LB	
83	PNPLA1	NM_001374623.1	c.745G>A	exonic	5	nonsynonymous	p.Glu249Lys	813/277218=0.003	LB	
99	PNPLA1	NM_001374623.1	c.745G>A	exonic	5	nonsynonymous	p.Glu249Lys	813/277218=0.003	LB	
103	PNPLA1	NM_001374623.1	c.745G>A	exonic	5	nonsynonymous	p.Glu249Lys	813/277218=0.003	LB	
62	PNPLA1	NM_001374623.1	c.922A>G	exonic	6	nonsynonymous	p.Thr308Ala		VOUS	
160	PNPLA1	NM_001374623.1	c.985T>C	exonic	6	nonsynonymous	p.Ser329Pro	615/276868=0.002	VOUS	
30	PNPLA1	NM_001374623.1	c.1464T>A	exonic	7	stopgain	p.Tyr488*	3104/273832=0.011	LB	
46	PNPLA1	NM_001374623.1	c.1464T>A	exonic	7	stopgain	p.Tyr488*	3104/273832=0.011	LB	
6	PNPLA1	NM_001374623.1	c.1464T>A	exonic	7	stopgain	p.Tyr488*	3104/273832=0.011	LB	
18	PNPLA1	NM_001374623.1	c.1464T>A	exonic	7	stopgain	p.Tyr488*	3104/273832=0.011	LB	
44	PNPLA1	NM_001374623.1	c.1464T>A	exonic	7	stopgain	p.Tyr488*	3104/273832=0.011	LB	
57	PNPLA1	NM_001374623.1	c.1464T>A	exonic	7	stopgain	p.Tyr488*	3104/273832=0.011	LB	
78	PNPLA1	NM_001374623.1	c.1464T>A	exonic	7	stopgain	p.Tyr488*	3104/273832=0.011	LB	
84	PNPLA1	NM_001374623.1	c.1464T>A	exonic	7	stopgain	p.Tyr488*	3104/273832=0.011	LB	
95	PNPLA1	NM_001374623.1	c.1464T>A	exonic	7	stopgain	p.Tyr488*	3104/273832=0.011	LB	
105	PNPLA1	NM_001374623.1	c.1464T>A	exonic	7	stopgain	p.Tyr488*	3104/273832=0.011	LB	
112	PNPLA1	NM_001374623.1	c.1464T>A	exonic	7	stopgain	p.Tyr488*	3104/273832=0.011	LB	
123	PNPLA1	NM_001374623.1	c.1464T>A	exonic	7	stopgain	p.Tyr488*	3104/273832=0.011	LB	
142	PNPLA1	NM_001374623.1	c.1464T>A	exonic	7	stopgain	p.Tyr488*	3104/273832=0.011	LB	
145	PNPLA1	NM_001374623.1	c.1464T>A	exonic	7	stopgain	p.Tyr488*	3104/273832=0.011	LB	
155	PNPLA1	NM_001374623.1	c.1464T>A	exonic	7	stopgain	p.Tyr488*	3104/273832=0.011	LB	
161	PNPLA1	NM_001374623.1	c.1464T>A	exonic	7	stopgain	p.Tyr488*	3104/273832=0.011	LB	
163	PNPLA1	NM_001374623.1	c.1464T>A	exonic	7	stopgain	p.Tyr488*	3104/273832=0.011	LB	
121	SERPINB7	NM_003784.4	c.220T>C	exonic	4	nonsynonymous	p.Ser74Pro	9/247468=0	VOUS	# 615598 PALMOPLANTAR KERATODERMA, NAGASHIMA TYPE; PPKN
133	SERPINB7	NM_003784.4	c.220T>C	exonic	4	nonsynonymous	p.Ser74Pro	9/247468=0	VOUS	
176	SERPINB7	NM_003784.4	c.220T>C	exonic	4	nonsynonymous	p.Ser74Pro	9/247468=0	VOUS	
192	SERPINB7	NM_003784.4	c.715G>A	exonic	7	nonsynonymous	p.Val239Ile	20/276514=0	VOUS	
97	SERPINB7	NM_003784.4	c.833A>G	exonic	8	nonsynonymous	p.Gln278Arg	42/275758=0	VOUS	
108	SERPINB7	NM_003784.4	c.833A>G	exonic	8	nonsynonymous	p.Gln278Arg	42/275758=0	VOUS	
127	SERPINB7	NM_003784.4	c.992A>C	exonic	8	nonsynonymous	p.Glu331Ala	113/276360=0	VOUS	
10	SERPINB8	NM_001366198.1	c.304C>T	exonic	3	nonsynonymous	p.Pro102Ser	352/276834=0.001	LB	# 617115 PEELING SKIN SYNDROME 5; PSS5 AR
128	SERPINB8	NM_001366198.1	c.254T>G	exonic	3	nonsynonymous	p.Leu85Trp	568/277052=0.002	VOUS	
137	SERPINB8	NM_001366198.1	c.254T>G	exonic	3	nonsynonymous	p.Leu85Trp	568/277052=0.002	B	
50	SERPINB8	NM_001366198.1	c.872C>G	exonic	7	nonsynonymous	p.Ala291Gly	60/277244=0	VOUS	
69	SERPINB8	NM_001366198.1	c.866T>C	exonic	7	nonsynonymous	p.Ile289Thr	9/246270=0	VOUS	
109	SERPINB8	NM_001366198.1	c.988G>A	exonic	7	nonsynonymous	p.Ala330Thr	61/276742=0	VOUS	
113	SERPINB8	NM_001366198.1	c.1121C>T	exonic	7	nonsynonymous	p.Pro374Leu	9/273242=0	VOUS	
65	SLC27A4	NM_005094.4	c.250G>A	exonic	3	nonsynonymous	p.Val84Ile	2/245924=0	VOUS	# 608649 ICHTHYOSIS PREMATURITY SYNDROME; IPS<br>Alternative titles; symbols ICHTHYOSIS CONGENITA IV AR
198	SLC27A4	NM_005094.4	c.742G>A	exonic	5	nonsynonymous	p.Gly248Ser		LP	
172	SLC27A4	NM_005094.4	c.952C>T	exonic	7	nonsynonymous	p.Arg318Trp	28/277118=0	VOUS	
147	SLC27A4	NM_005094.4	c.1300G>A	exonic	9	nonsynonymous	p.Gly434Ser	9/245968=0	VOUS	
16	SLC27A4	NM_005094.4	c.1415_1417delAGA	exonic	10	inframe	p.Lys472del	8/245700=0	VOUS	
179	SLC27A4	NM_005094.4	c.1788C>G	exonic	13	nonsynonymous	p.Phe596Leu	3/246250=0	VOUS	
45	SNAP29	NM_004782.4	c.130T>C	exonic	1	nonsynonymous	p.Tyr44His	649/251920=0.003	LB	# [illegible] CEREBRAL DYSGENESIS, NEUROPATHY, ICHTHYOSIS, AND PALMOPLANTAR KERATODERMA SYNDROME; CEDNIK AR
137	SNAP29	NM_004782.4	c.113C>T	exonic	1	nonsynonymous	p.Pro38Leu	139/246926=0.001	B	
65	SPINK5	NM_001127698.2	c.677A>G	exonic	9	nonsynonymous	p.Lys226Arg	8/276568=0	LB	
164	SPINK5	NM_001127698.2	c.1362G>C	exonic	15	nonsynonymous	p.Glu454Asp	1/245736=0	LB	
165	SPINK5	NM_001127698.2	c.1362G>C	exonic	15	nonsynonymous	p.Glu454Asp	1/245736=0	LB	

82	SPINK5	NM_001127698.2	c.1451G>A	exonic	16	nonsynonymous	p.Arg484Lys	343/276350=0.001	LB	
126	SPINK5	NM_001127698.2	c.1451G>A	exonic	16	nonsynonymous	p.Arg484Lys	343/276350=0.001	LB	
192	SPINK5	NM_001127698.2	c.1451G>A	exonic	16	nonsynonymous	p.Arg484Lys	343/276350=0.001	LB	
9	SPINK5	NM_001127698.2	c.1552C>T	exonic	17	nonsynonymous	p.Arg518Cys	879/277134=0.003	B	
48	SPINK5	NM_001127698.2	c.1964G>A	exonic	21	nonsynonymous	p.Gly655Asp	760/276990=0.003	LB	
91	SPINK5	NM_001127698.2	c.1964G>A	exonic	21	nonsynonymous	p.Gly655Asp	760/276990=0.003	LB	# 256500<br>NETHERTON SYNDROME; NETH
90	SPINK5	NM_001127698.2	c.2243A>G	exonic	24	nonsynonymous	p.Glu748Gly	800/276940=0.003	B	
97	SPINK5	NM_001127698.2	c.2243A>G	exonic	24	nonsynonymous	p.Glu748Gly	800/276940=0.003	B	
45	SPINK5	NM_001127698.2	c.2852A>G	exonic	30	nonsynonymous	p.Asn951Ser	1264/277148=0.005	LB	
100	SPINK5	NM_001127698.2	c.2954T>C	exonic	30	nonsynonymous	p.Val985Ala	982/276828=0.004	LB	
124	SPINK5	NM_001127698.2	c.2954T>C	exonic	30	nonsynonymous	p.Val985Ala	982/276828=0.004	LB	
171	SPINK5	NM_001127698.2	c.2852A>G	exonic	30	nonsynonymous	p.Asn951Ser	1264/277148=0.005	LB	
52	SPINK5	NM_001127698.2	c.3167T>G	exonic	32	nonsynonymous	p.Met1056Arg	26/246178=0	VOUS	
108	SPINK5	NM_001127698.2	c.3167T>G	exonic	32	nonsynonymous	p.Met1056Arg	26/246178=0	VOUS	
11	SPINK5	NM_001127698.2	c.3256G>A	exonic	33	nonsynonymous	p.Ala1086Thr	9/277070=0	LB	
186	ST14	NM_021978.4	c.145A>T	exonic	2	stopgain	p.Lys49*		P	
193	ST14	NM_021978.4	c.508G>A	exonic	5	nonsynonymous	p.Glu170Lys	521/276770=0.002	LB	
4	ST14	NM_021978.4	c.508G>A	exonic	5	nonsynonymous	p.Glu170Lys	521/276770=0.002	LB	
43	ST14	NM_021978.4	c.454A>G	exonic	5	nonsynonymous	p.Ile152Val	876/276060=0.003	LB	
124	ST14	NM_021978.4	c.454A>G	exonic	5	nonsynonymous	p.Ile152Val	876/276060=0.003	B	
146	ST14	NM_021978.4	c.508G>A	exonic	5	nonsynonymous	p.Glu170Lys	521/276770=0.002	VOUS	
10	ST14	NM_021978.4	c.830C>T	exonic	7	nonsynonymous	p.Thr277Met	168/254380=0.001	LB	
100	ST14	NM_021978.4	c.800C>A	exonic	7	nonsynonymous	p.Ser267Tyr	2/240850=0	VOUS	# 602400<br>ICHTHYOSIS, CONGENITAL, AUTOSOMAL RECESSIVE 11; ARCI11
159	ST14	NM_021978.4	c.967C>T	exonic	8	nonsynonymous	p.Arg323Trp	2/246262=0	VOUS	
102	ST14	NM_021978.4	c.1034G>A	exonic	9	nonsynonymous	p.Arg345His	302/277184=0.001	B	
103	ST14	NM_021978.4	c.1975A>G	exonic	16	nonsynonymous	p.Ile659Val	33/245026=0	LB	
159	ST14	NM_021978.4	c.1975A>G	exonic	16	nonsynonymous	p.Ile659Val	33/245026=0	LB	
157	ST14	NM_021978.4	c.2146G>C	exonic	17	nonsynonymous	p.Glu716Gln		VOUS	
194	ST14	NM_021978.4	c.2406+4G>C	intronic	18			193/212654=0.001	B	
169	ST14	NM_021978.4	c.2553G>C	exonic	19	nonsynonymous	p.Glu851Asp	6/276830=0	VOUS	
140	STS	NM_001320751.2	c.478C>T	exonic	7	nonsynonymous	p.His160Tyr		VOUS	
125	STS	NM_001320751.2	c.1147A>G	exonic	10	nonsynonymous	p.Ile383Val	9/178456=0	VOUS	# 308100<br>ICHTHYOSIS, X-LINKED; XLI
162	STS	NM_001320751.2	c.1168C>T	exonic	10	nonsynonymous	p.Arg390Cys	4/178511=0	VOUS	
177	STS	NM_001320751.2	c.1274A>G	exonic	10	nonsynonymous	p.Asp425Gly	184/199731=0.001	B	
134	SULT2B1	NM_004605.2	c.107T>C	exonic	1	nonsynonymous	p.Leu36Ser	1287/274634=0.005	B	
195	SULT2B1	NM_004605.2	c.107T>C	exonic	1	nonsynonymous	p.Leu36Ser	1287/274634=0.005	B	
140	SULT2B1	NM_004605.2	c.232C>T	exonic	2	nonsynonymous	p.Arg78Cys	252/276636=0.001	B	
167	SULT2B1	NM_004605.2	c.232C>T	exonic	2	nonsynonymous	p.Arg78Cys	252/276636=0.001	B	
132	SULT2B1	NM_004605.2	c.600+3G>A	intronic	4				VOUS	
75	SULT2B1	NM_004605.2	c.673G>A	exonic	5	nonsynonymous	p.Val225Ile	220/272762=0.001	VOUS	# 617571<br>ICHTHYOSIS, CONGENITAL, AUTOSOMAL RECESSIVE 14; ARCI14
24	SULT2B1	NM_004605.2	c.668G>A	exonic	5	nonsynonymous	p.Gly223Asp	3/272700=0	VOUS	
25	SULT2B1	NM_004605.2	c.867G>A	exonic	6	nonsynonymous	p.Met289Ile	270/262312=0.001	B	
64	SULT2B1	NM_004605.2	c.1045C>T	exonic	6	nonsynonymous	p.Pro349Ser	100/144788=0.001	VOUS	
202	SULT2B1	NM_004605.2	c.867G>A	exonic	6	nonsynonymous	p.Met289Ile	270/262312=0.001	B	
204	SULT2B1	NM_004605.2	c.867G>A	exonic	6	nonsynonymous	p.Met289Ile	270/262312=0.001	B	
137	SUMF1	NM_182760.4	c.59T>G	exonic	1	nonsynonymous	p.Leu20Ar	988/267246=0.004	B	
184	SUMF1	NM_182760.4	c.131G>A	exonic	1	nonsynonymous	p.Gly44Glu		VOUS	# 272200<br>MULTIPLE SULFATASE DEFICIENCY; MSD AR
129	SUMF1	NM_182760.4	c.664G>C	exonic	5	nonsynonymous	p.Gly222Arg	450/277142=0.002	VOUS	
107	SUMF1	NM_182760.4	c.935T>C	exonic	7	nonsynonymous	p.Val312Ala		VOUS	
59	TGM1	NM_000359.3	c.61A>G	exonic	2	nonsynonymous	p.Thr21Ala	372/276110=0.001	LB	
139	TGM1	NM_000359.3	c.90_95dupGCCAGA	exonic	2	inframe	p.Glu30_Pro31dup	132/276714=0	LB	
152	TGM1	NM_000359.3	c.208G>T	exonic	2	nonsynonymous	p.Gly70Cys		VOUS	
189	TGM1	NM_000359.3	c.208G>T	exonic	2	nonsynonymous	p.Gly70Cys		VOUS	
5	TGM1	NM_000359.3	c.359C>T	exonic	3	nonsynonymous	p.Ser120Leu	7/245944=0	VOUS	
76	TGM1	NM_000359.3	c.746C>T	exonic	4	nonsynonymous	p.Pro249Leu		VOUS	
61	TGM1	NM_000359.3	c.680A>G	exonic	4	nonsynonymous	p.Gln227Arg		VOUS	# 242300<br>ICHTHYOSIS, CONGENITAL, AUTOSOMAL RECESSIVE 1; ARCI1
179	TGM1	NM_000359.3	c.550C>T	exonic	4	nonsynonymous	p.Pro184Ser	183/277094=0.001	VOUS	
187	TGM1	NM_000359.3	c.550C>T	exonic	4	nonsynonymous	p.Pro184Ser	183/277094=0.001	LB	

42	TGM1	NM_000359.3	c.920G>A	exonic	6	nonsynonymous	p.Arg307Gln	124/275156=0	B	
77	TGM1	NM_000359.3	c.1492-19T>A	intronic	11			7/245872=0	VOUS	
35	TGM1	NM_000359.3	c.1717C>T	exonic	12	nonsynonymous	p.Arg573Trp	7/277016=0	VOUS	
92	TGM1	NM_000359.3	c.2338G>A	exonic	15	nonsynonymous	p.Gly780SeR	1/246154=0	VOUS	
124	TGM1	NM_000359.3	c.2405A>T	exonic	15	nonsynonymous	p.Asp802Val	262/277042=0.001	B	
98	VPS33B	NM_018668.4	c.97-3C>T	intronic	2				VOUS	# 620009 KERATODERMA-ICHTHYOSIS-DEAFNESS SYNDROME, AUTOSOMAL RECESSIVE; KDIDAR
147	VPS33B	NM_018668.4	c.1166G>A	exonic	15	nonsynonymous	p.Arg389Gln	590/277118=0.002	B	
170	VPS33B	NM_018668.4	c.1307A>G	exonic	18	nonsynonymous	p.Asn436Ser	15/277084=0	VOUS	
174	VPS33B	NM_018668.4	c.1274G>A	exonic	18	nonsynonymous	p.Ser425Asn	350/277058=0.001	B	
203	VPS33B	NM_018668.4	c.1837A>T	exonic	23	nonsynonymous	p.Ser613Cys		VOUS	
111	ZMPSTE24	NM_005857.3	c.1106G>A	exonic	9	nonsynonymous	p.Arg369Gln	199/277132=0.001	B	# 275210 RESTRICTIVE DERMOPATHY 1; RSDM1 AR
118	ZMPSTE24	NM_005857.3	c.1235G>A	exonic	10	nonsynonymous	p.Arg412His	11/246164=0	VOUS	

c.730G>T

c.3167T>G

Table S2. Number of different rare variants identified in ichthyosis-related genes of 300 unaffected individuals

GENE	GenBank #	Nucleotide variant	gene position	Exon	Presumed effect	Presumed protein variant	gnomAD frequency
ABCA12	NM_173076.3	c.300T>G	exonic	3	nonsynonymous	p.Asp100Glu	6/276566=0
ABCA12	NM_173076.3	c.346G>T	exonic	4	nonsynonymous	p.Asp116Tyr	7/245398=0
ABCA12	NM_173076.3	c.485C>T	exonic	5	nonsynonymous	p.Ala162Val	384/276512=0.001
ABCA12	NM_173076.3	c.501G>C	exonic	5	nonsynonymous	p.Leu167Phe	
ABCA12	NM_173076.3	c.539T>C	exonic	6	nonsynonymous	p.Ile180Thr	3/245112=0
ABCA12	NM_173076.3	c.1141G>C	exonic	10	nonsynonymous	p.Val381Leu	239/277242=0.001
ABCA12	NM_173076.3	c.1222T>C	exonic	11	nonsynonymous	p.Ser408Pro	322/276876=0.001
ABCA12	NM_173076.3	c.1446A>C	exonic	12	nonsynonymous	p.Glu482Asp	29/276918=0
ABCA12	NM_173076.3	c.1475A>G	exonic	12	nonsynonymous	p.Asn492Ser	5/276916=0
ABCA12	NM_173076.3	c.1743C>G	exonic	14	nonsynonymous	p.Asp581Glu	246/277138=0.001
ABCA12	NM_173076.3	c.1816G>A	exonic	15	nonsynonymous	p.Asp606Asn	2/245978=0
ABCA12	NM_173076.3	c.2129A>G	exonic	17	nonsynonymous	p.Tyr710Cys	1/246028=0
ABCA12	NM_173076.3	c.2243G>A	exonic	17	nonsynonymous	p.Arg748Lys	6/245968=0
ABCA12	NM_173076.3	c.3098T>C	exonic	22	nonsynonymous	p.Ile1033Thr	2/245894=0
ABCA12	NM_173076.3	c.3481A>T	exonic	24	nonsynonymous	p.Met1161Leu	363/277160=0.001
ABCA12	NM_173076.3	c.4618G>T	exonic	31	nonsynonymous	p.Ala1540Ser	9/245910=0
ABCA12	NM_173076.3	c.5051T>C	exonic	33	nonsynonymous	p.Ile1684Thr	4/245586=0
ABCA12	NM_173076.3	c.5617G>A	exonic	37	nonsynonymous	p.Val1873Ile	670/276578=0.002
ABCA12	NM_173076.3	c.6208G>A	exonic	42	nonsynonymous	p.Val2070Ile	514/276952=0.002
ABCA12	NM_173076.3	c.6704A>C	exonic	45	nonsynonymous	p.Glu2235Ala	184/277156=0.001
ABCA12	NM_173076.3	c.6919A>G	exonic	46	nonsynonymous	p.Ile2307Val	486/277078=0.002
ABCA12	NM_173076.3	c.7631C>T	exonic	52	nonsynonymous	p.Thr2544Ile	379/276596=0.001
ABHD5	NM_001365649.1	c.22A>G	exonic	3	nonsynonymous	p.Thr8Ala	12/237020=0
ABHD5	NM_001365649.1	c.505C>G	exonic	4	nonsynonymous	p.Pro169Ala	21/277218=0
ABHD5	NM_001365649.1	c.883G>T	exonic	7	stopgain	p.Glu295*	1/121404=0
ALDH3A2	NM_001031806.2	c.119A>G	exonic	1	nonsynonymous	p.Asp40Gly	155/218008=0.001
ALDH3A2	NM_001031806.2	c.28C>G	exonic	1	nonsynonymous	p.Gln10Glu	806/218946=0.004
ALDH3A2	NM_001031806.2	c.17G>C	exonic	2	nonsynonymous	p.Arg6Pro	3/223156=0
ALDH3A2	NM_001031806.2	c.661G>A	exonic	4	nonsynonymous	p.Asp221Asn	1/244348=0
ALDH3A2	NM_001031806.2	c.1270C>T	exonic	9	nonsynonymous	p.Pro424Ser	1205/277210=0.004
ALOX12B	NM_001139.3	c.280G>A	exonic	2	nonsynonymous	p.Gly94Ser	2588/264690
ALOX12B	NM_001139.3	c.380C>T	exonic	3	nonsynonymous	p.Pro127Leu	66/276792=0
ALOX12B	NM_001139.3	c.526G>A	exonic	4	nonsynonymous	p.Glu176Lys	92/276916=0
ALOX12B	NM_001139.3	c.556A>T	exonic	5	nonsynonymous	p.Ile186Phe	0
ALOX12B	NM_001139.3	c.715A>G	exonic	6	nonsynonymous	p.Ile239Val	24/277224=0
ALOX12B	NM_001139.3	c.1156C>T	exonic	9	nonsynonymous	p.Arg386Cys	9/276770=0
ALOX12B	NM_001139.3	c.1431delC	exonic	11	frameshift	p.Asp477Glufs*37	1/245856=0
ALOX12B	NM_001139.3	c.1565C>T	exonic	12	nonsynonymous	p.Pro522Leu	277/277210=0.001
ALOXE3	NM_001165960.1	c.30G>T	exonic	1	nonsynonymous	p.Leu10Phe	

ALOXE3	NM_001165960.1	c.62C>T	exonic	1	nonsynonymous	p.Pro21Leu	16/168304=0
ALOXE3	NM_001165960.1	c.280C>G	exonic	2	nonsynonymous	p.Pro94Ala	143/182810=0.001
ALOXE3	NM_001165960.1	c.809G>A	exonic	4	nonsynonymous	p.Arg270Gln	7/246260=0
ALOXE3	NM_001165960.1	c.989T>C	exonic	6	nonsynonymous	p.Ile330Thr	4/246268=0
ALOXE3	NM_001165960.1	c.1076C>T	exonic	6	nonsynonymous	p.Ala359Val	51/246268=0
ALOXE3	NM_001165960.1	c.1454T>A	exonic	9	nonsynonymous	p.Leu485Gln	
ALOXE3	NM_001165960.1	c.1483C>T	exonic	9	nonsynonymous	p.Pro495Ser	
ALOXE3	NM_001165960.1	c.1843C>T	exonic	12	nonsynonymous	p.His615Tyr	
ALOXE3	NM_001165960.1	c.2102C>A	exonic	14	nonsynonymous	p.Thr701Asn	102/276992=0
ALOXE3	NM_001165960.1	c.2404C>T	exonic	16	nonsynonymous	p.Arg802Trp	239/277182=0.001
ALOXE3	NM_001165960.1	c.2510T>C	exonic	16	nonsynonymous	p.Ile837Thr	333/277202=0.001
CDSN	NM_001264.4	c.32G>A	exonic	1	nonsynonymous	p.Arg11His	790/239488=0.003
CDSN	NM_001264.4	c.1302C>A	exonic	2	nonsynonymous	p.Ser434Arg	916/276996=0.003
CDSN	NM_001264.4	c.475A>G	exonic	2	nonsynonymous	p.Ser159Gly	47/276174=0
CDSN	NM_001264.4	c.782G>T	exonic	2	nonsynonymous	p.Gly261Val	
CAST	NM_001750.7	c.200C>T	exonic	3	nonsynonymous	p.Ser67Leu	12/276074=0
CAST	NM_001750.7	c.620C>T	exonic	9	nonsynonymous	p.Pro207Leu	5/246206=0
CAST	NM_001750.7	c.775A>G	exonic	11	nonsynonymous	p.Thr259Ala	943/276512=0.003
CAST	NM_001750.7	c.925A>C	exonic	14	nonsynonymous	p.Ile309Leu	2994/276670=0.011
CAST	NM_001750.7	c.986C>G	exonic	14	nonsynonymous	p.Ala329Gly	74/276982=0
CAST	NM_001750.7	c.1177C>T	exonic	16	nonsynonymous	p.Arg393Cys	34/276754=0
CAST	NM_001750.7	c.1207G>C	exonic	17	nonsynonymous	p.Ala403Pro	1334/276854=0.005
CAST	NM_001750.7	c.1283C>T	exonic	17	nonsynonymous	p.Thr428Met	909/277064=0.003
CAST	NM_001750.7	c.1835A>G	exonic	25	nonsynonymous	p.Lys612Arg	585/265502=0.002
CLDN1	NM_021101.5	c.136A>T	exonic	1	nonsynonymous	p.Met46Leu	15/246242=0
CLDN1	NM_021101.5	c.278T>C	exonic	2	nonsynonymous	p.Ile93Thr	
CLDN1	NM_021101.5	c.631G>A	exonic	4	nonsynonymous	p.Val211Met	30/277152=0
CERS3	NM_001290341.2	c.233C>T	exonic	6	nonsynonymous	p.Ser78Leu	
CERS3	NM_001290341.2	c.914A>G	exonic	13	nonsynonymous	p.His305Arg	3292/264968=0.012
CERS3	NM_001290341.2	c.1151G>A	exonic	14	nonsynonymous	p.Arg384Lys	481/277162=0.002
CYP4F22	NM_173483.4	c.109C>T	exonic	3	nonsynonymous	p.Arg37Cys	32/121338=0
CYP4F22	NM_173483.4	c.68C>T	exonic	3	nonsynonymous	p.Ala23Val	45/277126=0
CYP4F22	NM_173483.4	c.463C>T	exonic	6	nonsynonymous	p.His155Tyr	121/277112=0
CYP4F22	NM_173483.4	c.485C>G	exonic	6	nonsynonymous	p.Ala162Gly	47/277122=0
CYP4F22	NM_173483.4	c.665G>T	exonic	7	nonsynonymous	p.Cys222Phe	
CYP4F22	NM_173483.4	c.712G>A	exonic	8	nonsynonymous	p.Ala238Thr	
CYP4F22	NM_173483.4	c.851G>A	exonic	8	nonsynonymous	p.Arg284Gln	9/276864=0
CYP4F22	NM_173483.4	c.1148C>T	exonic	11	nonsynonymous	p.Thr383Ile	
ELOVL4	NM_022726.4	c.243C>G	exonic	2	nonsynonymous	p.Ile81Met	
ELOVL4	NM_022726.4	c.800T>C	exonic	6	nonsynonymous	p.Ile267Thr	2004/276984=0.007
ELOVL4	NM_022726.4	c.814G>C	exonic	6	nonsynonymous	p.Glu272Gln	2735/277052=0.01
GJA1	NM_000165.5	c.1108C>T	exonic	2	nonsynonymous	p.Arg370Cys	3/245356=0

GJA1	NM_000165.5	c.1109G>A	exonic	2	nonsynonymous	p.Arg370His	1/245340=0
GJA1	NM_000165.5	c.157C>T	exonic	2	nonsynonymous	p.Arg53Cys	2/246262=0
GJA1	NM_000165.5	c.758C>T	exonic	2	nonsynonymous	p.Ala253Val	2227/277148=0.008
GJB2	NM_004004.6	c.101T>C	exonic	2	nonsynonymous	p.Met34Thr	2487/276420=0.009
GJB2	NM_004004.6	c.109G>A	exonic	2	nonsynonymous	p.Val37Ile	2011/276450=0.007
GJB2	NM_004004.6	c.23C>T	exonic	2	nonsynonymous	p.Thr8Met	21/275096=0
GJB2	NM_004004.6	c.269T>C	exonic	2	nonsynonymous	p.Leu90Pro	177/277032=0.001
GJB2	NM_004004.6	c.296G>A	exonic	2	nonsynonymous	p.Arg99Lys	
GJB2	NM_004004.6	c.358_360delGAG	exonic	2	inframe	p.Glu120del	20/275990=0
GJB2	NM_004004.6	c.35delG	exonic	2	frameshift	p.Gly12Valfs*2	1721/275002=0.006
GJB2	NM_004004.6	c.457G>A	exonic	2	nonsynonymous	p.Val153Ile	2433/276862=0.009
GJB2	NM_004004.6	c.467T>A	exonic	2	nonsynonymous	p.Val156Asp	
GJB2	NM_004004.6	c.88A>G	exonic	2	nonsynonymous	p.Ile30Val	
GJB3	NM_024009.3	c.196_198delGAC	exonic	2	inframe	p.Asp66del	38/277154=0
GJB3	NM_024009.3	c.293G>A	exonic	2	nonsynonymous	p.Arg98His	23/276870=0
GJB3	NM_024009.3	c.316C>T	exonic	2	nonsynonymous	p.Arg106Cys	40/276618=0
GJB3	NM_024009.3	c.422T>C	exonic	2	nonsynonymous	p.Ile141Thr	
GJB3	NM_024009.3	c.529T>G	exonic	2	nonsynonymous	p.Tyr177Asp	525/276788=0.002
GJB3	NM_024009.3	c.659A>T	exonic	2	nonsynonymous	p.Lys220Met	1/246042=0
GJB3	NM_024009.3	c.670C>T	exonic	2	stopgain	p.Arg224*	11/276538=0
GJB4	NM_153212.3	c.119C>T	exonic	2	nonsynonymous	p.Ala40Val	13/277082=0
GJB4	NM_153212.3	c.153delT	exonic	2	frameshift	p.Phe51Leufs*57	1464/276392=0.005
GJB4	NM_153212.3	c.254C>T	exonic	2	nonsynonymous	p.Thr85Met	8/245960=0
GJB4	NM_153212.3	c.314A>G	exonic	2	nonsynonymous	p.His105Arg	10/277130=0
GJB4	NM_153212.3	c.384G>A	exonic	2	stopgain	p.Trp128*	527/277132=0.002
GJB4	NM_153212.3	c.386G>A	exonic	2	stopgain	p.Trp129*	59/277112=0
GJB4	NM_153212.3	c.389C>T	exonic	2	nonsynonymous	p.Thr130Met	30/277058=0
GJB4	NM_153212.3	c.478C>T	exonic	2	nonsynonymous	p.Arg160Cys	20/276732=0
GJB4	NM_153212.3	c.770C>T	exonic	2	nonsynonymous	p.Ser257Leu	20/275366=0
GJB6	NM_001370092.1	c.212T>C	exonic	5	nonsynonymous	p.Val71Ala	110/277050=0
GJB6	NM_001370092.1	c.607A>G	exonic	5	nonsynonymous	p.Met203Val	247/277132=0.001
GJB6	NM_001370092.1	c.688A>T	exonic	5	nonsynonymous	p.Asn230Tyr	
KRT1	NM_006121.4	c.860T>C	exonic	3	nonsynonymous	p.Ile287Thr	1/246176=0
KRT1	NM_006121.4	c.982A>T	exonic	5	nonsynonymous	p.Thr328Ser	332/277126=0.001
KRT1	NM_006121.4	c.1294C>T	exonic	7	nonsynonymous	p.Arg432Cys	143/277212=0.001
KRT1	NM_006121.4	c.1390G>A	exonic	7	nonsynonymous	p.Asp464Asn	1/246258=0
KRT1	NM_006121.4	c.1693A>G	exonic	9	nonsynonymous	p.Ser565Gly	
KRT1	NM_006121.4	c.1894G>A	exonic	9	nonsynonymous	p.Val632Met	2/245184=0
KRT1	NM_006121.4	c.1912A>G	exonic	9	nonsynonymous	p.Thr638Ala	35/276622=0
KRT10	NM_001379366.1	c.158G>A	exonic	1	nonsynonymous	p.Ser53Asn	65/276636=0
KRT10	NM_001379366.1	c.257G>A	exonic	1	nonsynonymous	p.Arg86His	397/264258=0.002

KRT10	NM_001379366.1	c.71G>A	exonic	1	nonsynonymous	p.Gly24Glu	34/179406=0
KRT10	NM_001379366.1	c.98C>T	exonic	1	nonsynonymous	p.Ser33Phe	72/274952=0
KRT10	NM_001379366.1	c.710+6T>C	intronic	2			5/277170=0
KRT10	NM_001379366.1	c.1443_1457delAAGCTCCGGCGGCGG	exonic	7	inframe	p.Ser482_Gly486del	1/99466=0
KRT10	NM_001379366.1	c.1471_1479delCACGGCGGC	exonic	7	inframe	p.His491_Gly493del	
KRT10	NM_001379366.1	c.1495T>C	exonic	7	nonsynonymous	p.Tyr499His	79/97856=0.001
KRT10	NM_001379366.1	c.1524C>G	exonic	7	nonsynonymous	p.Ser508Arg	153/101426=0.002
KRT10	NM_001379366.1	c.1650_1667delCAGCAGCTCCGGCGGCGG	exonic	7	inframe	p.Ser551_Gly556del	34/219570=0
KRT2	NM_000423.3	c.146G>A	exonic	1	nonsynonymous	p.Gly49Asp	3/242214=0
KRT2	NM_000423.3	c.317G>A	exonic	1	nonsynonymous	p.Ser106Asn	641/254108=0.003
KRT2	NM_000423.3	c.767A>G	exonic	2	nonsynonymous	p.Asn256Ser	110/277226=0
KRT2	NM_000423.3	c.1550C>G	exonic	9	nonsynonymous	p.Ala517Gly	993/277108=0.004
KRT2	NM_000423.3	c.1750A>G	exonic	9	nonsynonymous	p.Ile584Val	
KRT9	NM_000226.4	c.245G>A	exonic	1	nonsynonymous	p.Ser82Asn	628/274158=0.002
KRT9	NM_000226.4	c.49G>A	exonic	1	nonsynonymous	p.Gly17Ser	
KRT9	NM_000226.4	c.1049C>G	exonic	5	nonsynonymous	p.Thr350Ser	
KRT9	NM_000226.4	c.1630G>A	exonic	7	nonsynonymous	p.Gly544Arg	10/179160=0
LIPN	NM_001102469.1	c.302delG	exonic	3	frameshift	p.Gly101Glufs*7	50/276298=0
LIPN	NM_001102469.1	c.326A>C	exonic	3	nonsynonymous	p.Asp109Ala	2/245360=0
LIPN	NM_001102469.1	c.633T>G	exonic	5	nonsynonymous	p.Ile211Met	12/275930=0
LIPN	NM_001102469.1	c.754C>T	exonic	6	nonsynonymous	p.Leu252Phe	872/201394=0.004
LIPN	NM_001102469.1	c.772G>A	exonic	6	nonsynonymous	p.Glu258Lys	316/215004=0.001
LIPN	NM_001102469.1	c.934G>T	exonic	8	nonsynonymous	p.Asp312Tyr	1/152140=0
NIPAL4	NM_001099287.1	c.176C>A	exonic	1	nonsynonymous	p.Ala59Asp	
NIPAL4	NM_001099287.1	c.86C>A	exonic	1	stopgain	p.Ser29*	3/121822=0
NIPAL4	NM_001099287.1	c.238C>A	exonic	2	nonsynonymous	p.Leu80Ile	
NIPAL4	NM_001099287.1	c.296T>C	exonic	2	nonsynonymous	p.Val99Ala	783/277214=0.003
NIPAL4	NM_001099287.1	c.397G>A	exonic	2	nonsynonymous	p.Gly133Ser	9/277110=0
NIPAL4	NM_001099287.1	c.446C>T	exonic	2	nonsynonymous	p.Thr149Met	294/266230=0.001
NIPAL4	NM_001099287.1	c.581C>T	exonic	4	nonsynonymous	p.Thr194Met	1/243716=0
NIPAL4	NM_001099287.1	c.730G>T	exonic	5	nonsynonymous	p.Val244Phe	170/254010=0.001
NIPAL4	NM_001099287.1	c.1105G>A	exonic	6	nonsynonymous	p.Val369Ile	127/276938=0
NIPAL4	NM_001099287.1	c.839G>A	exonic	6	nonsynonymous	p.Arg280His	60/276956=0
NIPAL4	NM_001099287.1	c.965G>A	exonic	6	nonsynonymous	p.Arg322Gln	10/246210=0
PEX7	NM_000288.4	c.377A>C	exonic	4	nonsynonymous	p.Gln126Pr	1104/277164=0.004
PEX7	NM_000288.4	c.961A>T	exonic	10	nonsynonymous	p.Ile321Phe	
PHYH	NM_001323080.2	c.56C>T	exonic	4	nonsynonymous	p.Thr19Met	367/277256=0.001
PHYH	NM_001323080.2	c.301C>G	exonic	6	nonsynonymous	p.Arg101Gly	351/277160=0.001
PHYH	NM_001323080.2	c.403G>A	exonic	7	nonsynonymous	p.Gly135Arg	2/246256=0
PNPLA1	NM_001374623.1	c.116C>G	exonic	1	nonsynonymous	p.Ala39Gly	
PNPLA1	NM_001374623.1	c.383C>T	exonic	2	nonsynonymous	p.Thr128Met	397/276794=0.001

PNPLA1	NM_001374623.1	c.472T>A	exonic	3	nonsynonymous	p.Cys158Ser	
PNPLA1	NM_001374623.1	c.592G>T	exonic	4	nonsynonymous	p.Asp198Tyr	
PNPLA1	NM_001374623.1	c.714+7G>A	intronic	4			2/244890=0
PNPLA1	NM_001374623.1	c.745G>A	exonic	5	nonsynonymous	p.Glu249Lys	813/277218=0.003
PNPLA1	NM_001374623.1	c.922A>G	exonic	6	nonsynonymous	p.Thr308Ala	
PNPLA1	NM_001374623.1	c.985T>C	exonic	6	nonsynonymous	p.Ser329Pro	615/276868=0.002
PNPLA1	NM_001374623.1	c.1464T>A	exonic	7	stopgain	p.Tyr488*	3104/273832=0.011
SERPINB7	NM_003784.4	c.220T>C	exonic	4	nonsynonymous	p.Ser74Pro	9/247468=0
SERPINB7	NM_003784.4	c.715G>A	exonic	7	nonsynonymous	p.Val239Ile	20/276514=0
SERPINB7	NM_003784.4	c.833A>G	exonic	8	nonsynonymous	p.Gln278Arg	42/275758=0
SERPINB7	NM_003784.4	c.992A>C	exonic	8	nonsynonymous	p.Glu331Ala	113/276360=0
SERPINB8	NM_001366198.1	c.254T>G	exonic	3	nonsynonymous	p.Leu85Trp	568/277052=0.002
SERPINB8	NM_001366198.1	c.304C>T	exonic	3	nonsynonymous	p.Pro102Ser	352/276834=0.001
SERPINB8	NM_001366198.1	c.1121C>T	exonic	7	nonsynonymous	p.Pro374Leu	9/273242=0
SERPINB8	NM_001366198.1	c.866T>C	exonic	7	nonsynonymous	p.Ile289Thr	9/246270=0
SERPINB8	NM_001366198.1	c.872C>G	exonic	7	nonsynonymous	p.Ala291Gly	60/277244=0
SERPINB8	NM_001366198.1	c.988G>A	exonic	7	nonsynonymous	p.Ala330Thr	61/276742=0
SNAP29	NM_004782.4	c.113C>T	exonic	1	nonsynonymous	p.Pro38Leu	139/246926=0.001
SNAP29	NM_004782.4	c.130T>C	exonic	1	nonsynonymous	p.Tyr44His	649/251920=0.003
SLC27A4	NM_005094.4	c.250G>A	exonic	3	nonsynonymous	p.Val84Ile	2/245924=0
SLC27A4	NM_005094.4	c.742G>A	exonic	5	nonsynonymous	p.Gly248Ser	
SLC27A4	NM_005094.4	c.952C>T	exonic	7	nonsynonymous	p.Arg318Trp	28/277118=0
SLC27A4	NM_005094.4	c.1300G>A	exonic	9	nonsynonymous	p.Gly434Ser	9/245968=0
SLC27A4	NM_005094.4	c.1415_1417delAGA	exonic	10	inframe	p.Lys472del	8/245700=0
SLC27A4	NM_005094.4	c.1788C>G	exonic	13	nonsynonymous	p.Phe596Leu	3/246250=0
SPINK5	NM_001127698.2	c.677A>G	exonic	9	nonsynonymous	p.Lys226Arg	8/276568=0
SPINK5	NM_001127698.2	c.1362G>C	exonic	15	nonsynonymous	p.Glu454Asp	1/245736=0
SPINK5	NM_001127698.2	c.1451G>A	exonic	16	nonsynonymous	p.Arg484Lys	343/276350=0.001
SPINK5	NM_001127698.2	c.1552C>T	exonic	17	nonsynonymous	p.Arg518Cys	879/277134=0.003
SPINK5	NM_001127698.2	c.1964G>A	exonic	21	nonsynonymous	p.Gly655Asp	760/276990=0.003
SPINK5	NM_001127698.2	c.2243A>G	exonic	24	nonsynonymous	p.Glu748Gly	800/276940=0.003
SPINK5	NM_001127698.2	c.2852A>G	exonic	30	nonsynonymous	p.Asn951Ser	1264/277148=0.005
SPINK5	NM_001127698.2	c.2954T>C	exonic	30	nonsynonymous	p.Val985Ala	982/276828=0.004
SPINK5	NM_001127698.2	c.3167T>G	exonic	32	nonsynonymous	p.Met1056Arg	26/246178=0
SPINK5	NM_001127698.2	c.3256G>A	exonic	33	nonsynonymous	p.Ala1086Thr	9/277070=0
ST14	NM_021978.4	c.145A>T	exonic	2	stopgain	p.Lys49*	
ST14	NM_021978.4	c.454A>G	exonic	5	nonsynonymous	p.Ile152Val	876/276060=0.003
ST14	NM_021978.4	c.508G>A	exonic	5	nonsynonymous	p.Glu170Lys	521/276770=0.002
ST14	NM_021978.4	c.800C>A	exonic	7	nonsynonymous	p.Ser267Tyr	2/240850=0
ST14	NM_021978.4	c.830C>T	exonic	7	nonsynonymous	p.Thr277Met	168/254380=0.001
ST14	NM_021978.4	c.967C>T	exonic	8	nonsynonymous	p.Arg323Trp	2/246262=0
ST14	NM_021978.4	c.1034G>A	exonic	9	nonsynonymous	p.Arg345His	302/277184=0.001

ST14	NM_021978.4	c.1975A>G	exonic	16	nonsynonymous	p.Ile659Val	33/245026=0
ST14	NM_021978.4	c.2146G>C	exonic	17	nonsynonymous	p.Glu716Gln	
ST14	NM_021978.4	c.2406+4G>C	intronic	18			193/212654=0.001
ST14	NM_021978.4	c.2553G>C	exonic	19	nonsynonymous	p.Glu851Asp	6/276830=0
STS	NM_001320751.2	c.478C>T	exonic	7	nonsynonymous	p.His160Tyr	
STS	NM_001320751.2	c.1147A>G	exonic	10	nonsynonymous	p.Ile383Val	9/178456=0
STS	NM_001320751.2	c.1168C>T	exonic	10	nonsynonymous	p.Arg390Cys	4/178511=0
STS	NM_001320751.2	c.1274A>G	exonic	10	nonsynonymous	p.Asp425Gly	184/199731=0.001
SULT2B1	NM_004605.2	c.107T>C	exonic	1	nonsynonymous	p.Leu36Ser	1287/274634=0.005
SULT2B1	NM_004605.2	c.232C>T	exonic	2	nonsynonymous	p.Arg78Cys	252/276636=0.001
SULT2B1	NM_004605.2	c.600+3G>A	intronic	4			
SULT2B1	NM_004605.2	c.673G>A	exonic	5	nonsynonymous	p.Val225Ile	220/272762=0.001
SULT2B1	NM_004605.2	c.668G>A	exonic	5	nonsynonymous	p.Gly223Asp	3/272700=0
SULT2B1	NM_004605.2	c.1045C>T	exonic	6	nonsynonymous	p.Pro349Ser	100/144788=0.001
SULT2B1	NM_004605.2	c.867G>A	exonic	6	nonsynonymous	p.Met289Ile	270/262312=0.001
SUMF1	NM_182760.4	c.131G>A	exonic	1	nonsynonymous	p.Gly44Glu	
SUMF1	NM_182760.4	c.59T>G	exonic	1	nonsynonymous	p.Leu20Ar	988/267246=0.004
SUMF1	NM_182760.4	c.664G>C	exonic	5	nonsynonymous	p.Gly222Arg	450/277142=0.002
SUMF1	NM_182760.4	c.935T>C	exonic	7	nonsynonymous	p.Val312Ala	
TGM1	NM_000359.3	c.208G>T	exonic	2	nonsynonymous	p.Gly70Cys	
TGM1	NM_000359.3	c.61A>G	exonic	2	nonsynonymous	p.Thr21Ala	372/276110=0.001
TGM1	NM_000359.3	c.90_95dupGCCAGA	exonic	2	inframe	p.Glu30_Pro31dup	132/276714=0
TGM1	NM_000359.3	c.359C>T	exonic	3	nonsynonymous	p.Ser120Leu	7/245944=0
TGM1	NM_000359.3	c.746C>T	exonic	4	nonsynonymous	p.Pro249Leu	
TGM1	NM_000359.3	c.550C>T	exonic	4	nonsynonymous	p.Pro184Ser	183/277094=0.001
TGM1	NM_000359.3	c.680A>G	exonic	4	nonsynonymous	p.Gln227Arg	
TGM1	NM_000359.3	c.920G>A	exonic	6	nonsynonymous	p.Arg307Gln	124/275156=0
TGM1	NM_000359.3	c.1492-19T>A	intronic	11			7/245872=0
TGM1	NM_000359.3	c.1717C>T	exonic	12	nonsynonymous	p.Arg573Trp	7/277016=0
TGM1	NM_000359.3	c.2338G>A	exonic	15	nonsynonymous	p.Gly780SeR	1/246154=0
TGM1	NM_000359.3	c.2405A>T	exonic	15	nonsynonymous	p.Asp802Val	262/277042=0.001
VPS33B	NM_018668.4	c.97-3C>T	intronic	2			
ZMPSTE24	NM_005857.3	c.1106G>A	exonic	9	nonsynonymous	p.Arg369Gln	199/277132=0.001
ZMPSTE24	NM_005857.3	c.1235G>A	exonic	10	nonsynonymous	p.Arg412His	11/246164=0
VPS33B	NM_018668.4	c.1166G>A	exonic	15	nonsynonymous	p.Arg389Gln	590/277118=0.002
VPS33B	NM_018668.4	c.1274G>A	exonic	18	nonsynonymous	p.Ser425Asn	350/277058=0.001
VPS33B	NM_018668.4	c.1307A>G	exonic	18	nonsynonymous	p.Asn436Ser	15/277084=0
VPS33B	NM_018668.4	c.1837A>T	exonic	23	nonsynonymous	p.Ser613Cys	

Table S3. Subjects with at least one rare variant in ichthyosis-related genes among 300 unaffected individuals

Subject ID	GENE	GenBank #	Nucleotide variant	gene position	Exon	Presumed effect	Presumed protein variant	gnomAD frequency
1	NIPAL4	NM_001099287.1	c.1105G>A	exonic	6	nonsynonymous	p.Val369Ile	127/276938=0
2	NIPAL4	NM_001099287.1	c.86C>A	exonic	1	stopgain	p.Ser29*	3/121822=0
3	CAST	NM_001750.7	c.200C>T	exonic	3	nonsynonymous	p.Ser67Leu	12/276074=0
3	GJB4	NM_153212.3	c.119C>T	exonic	2	nonsynonymous	p.Ala40Val	13/277082=0
4	ALOXE3	NM_001165960.1	c.62C>T	exonic	1	nonsynonymous	p.Pro21Leu	16/168304=0
4	CERS3	NM_001290341.2	c.233C>T	exonic	6	nonsynonymous	p.Ser78Leu	
4	ST14	NM_021978.4	c.508G>A	exonic	5	nonsynonymous	p.Glu170Lys	521/276770=0.002
5	ABCA12	NM_173076.3	c.3098T>C	exonic	22	nonsynonymous	p.Ile1033Thr	2/245894=0
5	TGM1	NM_000359.3	c.359C>T	exonic	3	nonsynonymous	p.Ser120Leu	7/245944=0
6	GJB2	NM_004004.6	c.35delG	exonic	2	frameshift	p.Gly12Valfs*2	1721/275002=0.006
6	PNPLA1	NM_001374623.1	c.1464T>A	exonic	7	stopgain	p.Tyr488*	3104/273832=0.011
7	ABCA12	NM_173076.3	c.2129A>G	exonic	17	nonsynonymous	p.Tyr710Cys	1/246028=0
7	GJB3	NM_024009.3	c.422T>C	exonic	2	nonsynonymous	p.Ile141Thr	
8	GJB2	NM_004004.6	c.88A>G	exonic	2	nonsynonymous	p.Ile30Val	
9	CAST	NM_001750.7	c.1207G>C	exonic	17	nonsynonymous	p.Ala403Pro	1334/276854=0.005
9	GJB3	NM_024009.3	c.529T>G	exonic	2	nonsynonymous	p.Tyr177Asp	525/276788=0.002
9	LIPN	NM_001102469.1	c.754C>T	exonic	6	nonsynonymous	p.Leu252Phe	872/201394=0.004
9	PNPLA1	NM_001374623.1	c.383C>T	exonic	2	nonsynonymous	p.Thr128Met	397/276794=0.001
9	SPINK5	NM_001127698.2	c.1552C>T	exonic	17	nonsynonymous	p.Arg518Cys	879/277134=0.003
10	KRT1	NM_006121.4	c.1294C>T	exonic	7	nonsynonymous	p.Arg432Cys	143/277212=0.001
10	SERPINB8	NM_001366198.1	c.304C>T	exonic	3	nonsynonymous	p.Pro102Ser	352/276834=0.001
10	ST14	NM_021978.4	c.830C>T	exonic	7	nonsynonymous	p.Thr277Met	168/254380=0.001
11	KRT1	NM_006121.4	c.860T>C	exonic	3	nonsynonymous	p.Ile287Thr	1/246176=0
11	SPINK5	NM_001127698.2	c.3256G>A	exonic	33	nonsynonymous	p.Ala1086Thr	9/277070=0
12	CAST	NM_001750.7	c.1835A>G	exonic	25	nonsynonymous	p.Lys612Arg	585/265502=0.002
12	GJB2	NM_004004.6	c.101T>C	exonic	2	nonsynonymous	p.Met34Thr	2487/276420=0.009
12	KRT10	NM_001379366.1	c.1495T>C	exonic	7	nonsynonymous	p.Tyr499His	79/97856=0.001
13	ALOXE3	NM_001165960.1	c.30G>T	exonic	1	nonsynonymous	p.Leu10Phe	
14	ABCA12	NM_173076.3	c.2243G>A	exonic	17	nonsynonymous	p.Arg748Lys	6/245968=0
15	ABCA12	NM_173076.3	c.1816G>A	exonic	15	nonsynonymous	p.Asp606Asn	2/245978=0
16	CDSN	NM_001264.4	c.32G>A	exonic	1	nonsynonymous	p.Arg11His	790/239488=0.003
16	SLC27A4	NM_005094.4	c.1415_1417delAGA	exonic	10	inframe	p.Lys472del	8/245700=0
17	GJB2	NM_004004.6	c.101T>C	exonic	2	nonsynonymous	p.Met34Thr	2487/276420=0.009
18	PNPLA1	NM_001374623.1	c.1464T>A	exonic	7	stopgain	p.Tyr488*	3104/273832=0.011
19	GJB4	NM_153212.3	c.254C>T	exonic	2	nonsynonymous	p.Thr85Met	8/245960=0
19	PNPLA1	NM_001374623.1	c.745G>A	exonic	5	nonsynonymous	p.Glu249Lys	813/277218=0.003
20	GJB2	NM_004004.6	c.457G>A	exonic	2	nonsynonymous	p.Val153Ile	2433/276862=0.009
21	KRT10	NM_001379366.1	c.257G>A	exonic	1	nonsynonymous	p.Arg86His	397/264258=0.002
22	GJB2	NM_004004.6	c.109G>A	exonic	2	nonsynonymous	p.Val37Ile	2011/276450=0.007

22	KRT2	NM_000423.3	c.146G>A	exonic	1	nonsynonymous	p.Gly49Asp	3/242214=0
23	CERS3	NM_001290341.2	c.1151G>A	exonic	14	nonsynonymous	p.Arg384Lys	481/277162=0.002
23	KRT10	NM_001379366.1	c.1524C>G	exonic	7	nonsynonymous	p.Ser508Arg	153/101426=0.002
24	GJB3	NM_024009.3	c.316C>T	exonic	2	nonsynonymous	p.Arg106Cys	40/276618=0
24	SULT2B1	NM_004605.2	c.668G>A	exonic	5	nonsynonymous	p.Gly223Asp	3/272700=0
25	GJA1	NM_000165.5	c.1108C>T	exonic	2	nonsynonymous	p.Arg370Cys	3/245356=0
25	GJB6	NM_001370092.1	c.688A>T	exonic	5	nonsynonymous	p.Asn230Tyr	
25	SULT2B1	NM_004605.2	c.867G>A	exonic	6	nonsynonymous	p.Met289Ile	270/262312=0.001
26	ALOX12B	NM_001139.3	c.380C>T	exonic	3	nonsynonymous	p.Pro127Leu	66/276792=0
27	KRT2	NM_000423.3	c.1550C>G	exonic	9	nonsynonymous	p.Ala517Gly	993/277108=0.004
28	PNPLA1	NM_001374623.1	c.745G>A	exonic	5	nonsynonymous	p.Glu249Lys	813/277218=0.003
29	ABCA12	NM_173076.3	c.6208G>A	exonic	42	nonsynonymous	p.Val2070Ile	514/276952=0.002
30	PNPLA1	NM_001374623.1	c.1464T>A	exonic	7	stopgain	p.Tyr488*	3104/273832=0.011
31	CLDN1	NM_021101.5	c.631G>A	exonic	4	nonsynonymous	p.Val211Met	30/277152=0
32	GJA1	NM_000165.5	c.1109G>A	exonic	2	nonsynonymous	p.Arg370His	1/245340=0
33	KRT2	NM_000423.3	c.1550C>G	exonic	9	nonsynonymous	p.Ala517Gly	993/277108=0.004
34	GJB4	NM_153212.3	c.770C>T	exonic	2	nonsynonymous	p.Ser257Leu	20/275366=0
34	KRT1	NM_006121.4	c.982A>T	exonic	5	nonsynonymous	p.Thr328Ser	332/277126=0.001
35	GJB2	NM_004004.6	c.35delG	exonic	2	frameshift	p.Gly12Valfs*2	1721/275002=0.006
35	TGM1	NM_000359.3	c.1717C>T	exonic	12	nonsynonymous	p.Arg573Trp	7/277016=0
36	ABCA12	NM_173076.3	c.5617G>A	exonic	37	nonsynonymous	p.Val1873Ile	670/276578=0.002
36	GJB2	NM_004004.6	c.269T>C	exonic	2	nonsynonymous	p.Leu90Pro	177/277032=0.001
37	CDSN	NM_001264.4	c.475A>G	exonic	2	nonsynonymous	p.Ser159Gly	47/276174=0
38	KRT9	NM_000226.4	c.245G>A	exonic	1	nonsynonymous	p.Ser82Asn	628/274158=0.002
39	KRT9	NM_000226.4	c.245G>A	exonic	1	nonsynonymous	p.Ser82Asn	628/274158=0.002
40	ALDH3A2	NM_001031806.2	c.1270C>T	exonic	9	nonsynonymous	p.Pro424Ser	1205/277210=0.004
41	ABCA12	NM_173076.3	c.5051T>C	exonic	33	nonsynonymous	p.Ile1684Thr	4/245586=0
42	GJB2	NM_004004.6	c.457G>A	exonic	2	nonsynonymous	p.Val153Ile	2433/276862=0.009
42	TGM1	NM_000359.3	c.920G>A	exonic	6	nonsynonymous	p.Arg307Gln	124/275156=0
43	ST14	NM_021978.4	c.454A>G	exonic	5	nonsynonymous	p.Ile152Val	876/276060=0.003
44	PNPLA1	NM_001374623.1	c.745G>A	exonic	5	nonsynonymous	p.Glu249Lys	813/277218=0.003
44	PNPLA1	NM_001374623.1	c.1464T>A	exonic	7	stopgain	p.Tyr488*	3104/273832=0.011
45	ALOXE3	NM_001165960.1	c.280C>G	exonic	2	nonsynonymous	p.Pro94Ala	143/182810=0.001
45	CAST	NM_001750.7	c.1835A>G	exonic	25	nonsynonymous	p.Lys612Arg	585/265502=0.002
45	SNAP29	NM_004782.4	c.130T>C	exonic	1	nonsynonymous	p.Tyr44His	649/251920=0.003
45	SPINK5	NM_001127698.2	c.2852A>G	exonic	30	nonsynonymous	p.Asn951Ser	1264/277148=0.005
46	GJB4	NM_153212.3	c.478C>T	exonic	2	nonsynonymous	p.Arg160Cys	20/276732=0
46	PNPLA1	NM_001374623.1	c.1464T>A	exonic	7	stopgain	p.Tyr488*	3104/273832=0.011
47	ALDH3A2	NM_001031806.2	c.17G>C	exonic	1	nonsynonymous	p.Arg6Pro	3/223156=0
48	SPINK5	NM_001127698.2	c.1964G>A	exonic	21	nonsynonymous	p.Gly655Asp	760/276990=0.003
49	ABCA12	NM_173076.3	c.4618G>T	exonic	31	nonsynonymous	p.Ala1540Ser	9/245910=0
50	LIPN	NM_001102469.1	c.326A>C	exonic	3	nonsynonymous	p.Asp109Ala	2/245360=0

50	SERPINB8	NM_001366198.1	c.872C>G	exonic	7	nonsynonymous	p.Ala291Gly	60/277244=0
51	PEX7	NM_000288.4	c.377A>C	exonic	4	nonsynonymous	p.Gln126Pr	1104/277164=0.004
52	CYP4F22	NM_173483.4	c.485C>G	exonic	6	nonsynonymous	p.Ala162Gly	47/277122=0
52	SPINK5	NM_001127698.2	c.3167T>G	exonic	32	nonsynonymous	p.Met1056Arg	26/246178=0
53	KRT10	NM_001379366.1	c.98C>T	exonic	1	nonsynonymous	p.Ser33Phe	72/274952=0
54	ABCA12	NM_173076.3	c.3481A>T	exonic	24	nonsynonymous	p.Met1161Leu	363/277160=0.001
54	GJB3	NM_024009.3	c.196_198delGAC	exonic	2	inframe	p.Asp66del	38/277154=0
55	ABCA12	NM_173076.3	c.3481A>T	exonic	24	nonsynonymous	p.Met1161Leu	363/277160=0.001
55	ALDH3A2	NM_001031806.2	c.17G>C	exonic	2	nonsynonymous	p.Arg6Pro	3/223156=0
56	NIPAL4	NM_001099287.1	c.965G>A	exonic	6	nonsynonymous	p.Arg322Gln	10/246210=0
57	PNPLA1	NM_001374623.1	c.1464T>A	exonic	7	stopgain	p.Tyr488*	3104/273832=0.011
58	NIPAL4	NM_001099287.1	c.238C>A	exonic	2	nonsynonymous	p.Leu80Ile	
58	PNPLA1	NM_001374623.1	c.592G>T	exonic	4	nonsynonymous	p.Asp198Tyr	
59	CYP4F22	NM_173483.4	c.1148C>T	exonic	11	nonsynonymous	p.Thr383Ile	
59	TGM1	NM_000359.3	c.61A>G	exonic	2	nonsynonymous	p.Thr21Ala	372/276110=0.001
60	CDSN	NM_001264.4	c.475A>G	exonic	2	nonsynonymous	p.Ser159Gly	47/276174=0
61	ELOVL4	NM_022726.4	c.800T>C	exonic	6	nonsynonymous	p.Ile267Thr	2004/276984=0.007
61	TGM1	NM_000359.3	c.680A>G	exonic	4	nonsynonymous	p.Gln227Arg	
62	ABCA12	NM_173076.3	c.3481A>T	exonic	24	nonsynonymous	p.Met1161Leu	363/277160=0.001
62	PNPLA1	NM_001374623.1	c.922A>G	exonic	6	nonsynonymous	p.Thr308Ala	
63	GJA1	NM_000165.5	c.758C>T	exonic	2	nonsynonymous	p.Ala253Val	2227/277148=0.008
64	CYP4F22	NM_173483.4	c.712G>A	exonic	8	nonsynonymous	p.Ala238Thr	
64	KRT9	NM_000226.4	c.1049C>G	exonic	5	nonsynonymous	p.Thr350Ser	
64	NIPAL4	NM_001099287.1	c.446C>T	exonic	2	nonsynonymous	p.Thr149Met	294/266230=0.001
64	SULT2B1	NM_004605.2	c.1045C>T	exonic	6	nonsynonymous	p.Pro349Ser	100/144788=0.001
65	ABCA12	NM_173076.3	c.7631C>T	exonic	52	nonsynonymous	p.Thr2544Ile	379/276596=0.001
65	SLC27A4	NM_005094.4	c.250G>A	exonic	3	nonsynonymous	p.Val84Ile	2/245924=0
65	SPINK5	NM_001127698.2	c.677A>G	exonic	9	nonsynonymous	p.Lys226Arg	8/276568=0
66	ABHD5	NM_001365649.1	c.22A>G	exonic	3	nonsynonymous	p.Thr8Ala	12/237020=0
67	ELOVL4	NM_022726.4	c.243C>G	exonic	2	nonsynonymous	p.Ile81Met	
68	ALOX12B	NM_001139.3	c.556A>T	exonic	5	nonsynonymous	p.Ile186Phe	0
69	SERPINB8	NM_001366198.1	c.866T>C	exonic	7	nonsynonymous	p.Ile289Thr	9/246270=0
70	GJB2	NM_004004.6	c.101T>C	exonic	2	nonsynonymous	p.Met34Thr	2487/276420=0.009
71	GJB2	NM_004004.6	c.35delG	exonic	2	frameshift	p.Gly12Valfs*2	1721/275002=0.006
72	CAST	NM_001750.7	c.986C>G	exonic	14	nonsynonymous	p.Ala329Gly	74/276982=0
72	CERS3	NM_001290341.2	c.914A>G	exonic	13	nonsynonymous	p.His305Arg	3292/264968=0.012
73	KRT9	NM_000226.4	c.245G>A	exonic	1	nonsynonymous	p.Ser82Asn	628/274158=0.002
74	ABCA12	NM_173076.3	c.346G>T	exonic	4	nonsynonymous	p.Asp116Tyr	7/245398=0
74	ABHD5	NM_001365649.1	c.22A>G	exonic	3	nonsynonymous	p.Thr8Ala	12/237020=0
75	SULT2B1	NM_004605.2	c.673G>A	exonic	5	nonsynonymous	p.Val225Ile	220/272762=0.001
76	ALDH3A2	NM_001031806.2	c.661G>A	exonic	4	nonsynonymous	p.Asp221Asn	1/244348=0
76	TGM1	NM_000359.3	c.746C>T	exonic	4	nonsynonymous	p.Pro249Leu	NUOVA

77	ABCA12	NM_173076.3	c.1222T>C	exonic	11	nonsynonymous	p.Ser408Pro	322/276876=0.001
77	CDSN	NM_001264.4	c.1302C>A	exonic	2	nonsynonymous	p.Ser434Arg	916/276996=0.003
77	GJB2	NM_004004.6	c.457G>A	exonic	2	nonsynonymous	p.Val153Ile	2433/276862=0.009
77	KRT1	NM_006121.4	c.1894G>A	exonic	9	nonsynonymous	p.Val632Met	2/245184=0
77	TGM1	NM_000359.3	c.1492-19T>A	intronic	11			7/245872=0
78	PNPLA1	NM_001374623.1	c.1464T>A	exonic	7	stopgain	p.Tyr488*	3104/273832=0.011
79	GJB2	NM_004004.6	c.457G>A	exonic	2	nonsynonymous	p.Val153Ile	2433/276862=0.009
80	ALOX12B	NM_001139.3	c.1156C>T	exonic	9	nonsynonymous	p.Arg386Cys	9/276770=0
81	CYP4F22	NM_173483.4	c.109C>T	exonic	3	nonsynonymous	p.Arg37Cys	32/121338=0
82	SPINK5	NM_001127698.2	c.1451G>A	exonic	16	nonsynonymous	p.Arg484Lys	343/276350=0.001
83	LIPN	NM_001102469.1	c.772G>A	exonic	6	nonsynonymous	p.Glu258Lys	316/215004=0.001
83	PNPLA1	NM_001374623.1	c.745G>A	exonic	5	nonsynonymous	p.Glu249Lys	813/277218=0.003
84	GJB2	NM_004004.6	c.35delG	exonic	2	frameshift	p.Gly12Valfs*2	1721/275002=0.006
84	PNPLA1	NM_001374623.1	c.1464T>A	exonic	7	stopgain	p.Tyr488*	3104/273832=0.011
85	PNPLA1	NM_001374623.1	c.745G>A	exonic	5	nonsynonymous	p.Glu249Lys	803/138609=0.006
86	CERS3	NM_001290341.2	c.914A>G	exonic	13	nonsynonymous	p.His305Arg	3292/264968=0.012
86	GJB4	NM_153212.3	c.254C>T	exonic	2	nonsynonymous	p.Thr85Met	8/245960=0
87	LIPN	NM_001102469.1	c.934G>T	exonic	8	nonsynonymous	p.Asp312Tyr	1/152140=0
88	GJB2	NM_004004.6	c.101T>C	exonic	2	nonsynonymous	p.Met34Thr	2487/276420=0.009
88	KRT10	NM_001379366.1	c.1471_1479delCACGGCGGC	exonic	7	inframe	p.His491_Gly493del	
89	ABCA12	NM_173076.3	c.1222T>C	exonic	11	nonsynonymous	p.Ser408Pro	322/276876=0.001
89	ABCA12	NM_173076.3	c.1475A>G	exonic	12	nonsynonymous	p.Asn492Ser	5/276916=0
89	ALDH3A2	NM_001031806.2	c.119A>G	exonic	1	nonsynonymous	p.Asp40Gly	155/218008=0.001
90	SPINK5	NM_001127698.2	c.2243A>G	exonic	24	nonsynonymous	p.Glu748Gly	800/276940=0.003
91	SPINK5	NM_001127698.2	c.1964G>A	exonic	21	nonsynonymous	p.Gly655Asp	760/276990=0.003
92	TGM1	NM_000359.3	c.2338G>A	exonic	15	nonsynonymous	p.Gly780SeR	1/246154=0
93	ALOX12B	NM_001139.3	c.1565C>T	exonic	12	nonsynonymous	p.Pro522Leu	277/277210=0.001
94	CYP4F22	NM_173483.4	c.68C>T	exonic	3	nonsynonymous	p.Ala23Val	45/277126=0
95	ALOXE3	NM_001165960.1	c.809G>A	exonic	4	nonsynonymous	p.Arg270Gln	7/246260=0
95	PNPLA1	NM_001374623.1	c.1464T>A	exonic	7	stopgain	p.Tyr488*	3104/273832=0.011
96	ABHD5	NM_001365649.1	c.883G>T	exonic	7	stopgain	p.Glu295*	1/121404=0
97	SERPINB7	NM_003784.4	c.833A>G	exonic	8	nonsynonymous	p.Gln278Arg	42/275758=0
97	SPINK5	NM_001127698.2	c.2243A>G	exonic	24	nonsynonymous	p.Glu748Gly	800/276940=0.003
98	CAST	NM_001750.7	c.775A>G	exonic	11	nonsynonymous	p.Thr259Ala	943/276512=0.003
98	CAST	NM_001750.7	c.925A>C	exonic	14	nonsynonymous	p.Ile309Leu	2994/276670=0.011
98	CDSN	NM_001264.4	c.32G>A	exonic	1	nonsynonymous	p.Arg11His	790/239488=0.003
98	VPS33B	NM_018668.4	c.97-3C>T	intronic	2			
99	PNPLA1	NM_001374623.1	c.745G>A	exonic	5	nonsynonymous	p.Glu249Lys	813/277218=0.003
100	SPINK5	NM_001127698.2	c.2954T>C	exonic	30	nonsynonymous	p.Val985Ala	982/276828=0.004
100	ST14	NM_021978.4	c.800C>A	exonic	7	nonsynonymous	p.Ser267Tyr	2/240850=0
101	GJB4	NM_153212.3	c.153delT	exonic	2	frameshift	p.Phe51Leufs*57	1464/276392=0.005
101	KRT9	NM_000226.4	c.1630G>A	exonic	7	nonsynonymous	p.Gly544Arg	10/179160=0

102	ST14	NM_021978.4	c.1034G>A	exonic	9	nonsynonymous	p.Arg345His	302/277184=0.001
103	GJB2	NM_004004.6	c.35delG	exonic	2	frameshift	p.Gly12Valfs*2	1721/275002=0.006
103	GJB4	NM_153212.3	c.384G>A	exonic	2	stopgain	p.Trp128*	527/277132=0.002
103	PNPLA1	NM_001374623.1	c.745G>A	exonic	5	nonsynonymous	p.Glu249Lys	813/277218=0.003
103	ST14	NM_021978.4	c.1975A>G	exonic	16	nonsynonymous	p.Ile659Val	33/245026=0
104	NIPAL4	NM_001099287.1	c.86C>A	exonic	1	stopgain	p.Ser29*	3/121822=0
105	PNPLA1	NM_001374623.1	c.1464T>A	exonic	7	stopgain	p.Tyr488*	3104/273832=0.011
106	ABCA12	NM_173076.3	c.300T>G	exonic	3	nonsynonymous	p.Asp100Glu	6/276566=0
107	PNPLA1	NM_001374623.1	c.714+7G>A	intronic	4			2/244890=0
107	SUMF1	NM_182760.4	c.935T>C	exonic	7	nonsynonymous	p.Val312Ala	
108	ABCA12	NM_173076.3	c.485C>T	exonic	5	nonsynonymous	p.Ala162Val	384/276512=0.001
108	SERPINB7	NM_003784.4	c.833A>G	exonic	8	nonsynonymous	p.Gln278Arg	42/275758=0
108	SPINK5	NM_001127698.2	c.3167T>G	exonic	32	nonsynonymous	p.Met1056Arg	26/246178=0
109	SERPINB8	NM_001366198.1	c.988G>A	exonic	7	nonsynonymous	p.Ala330Thr	61/276742=0
110	KRT10	NM_001379366.1	c.71G>A	exonic	1	nonsynonymous	p.Gly24Glu	34/179406=0
111	PNPLA1	NM_001374623.1	c.116C>G	exonic	1	nonsynonymous	p.Ala39Gly	
111	ZMPSTE24	NM_005857.3	c.1106G>A	exonic	9	nonsynonymous	p.Arg369Gln	199/277132=0.001
112	PNPLA1	NM_001374623.1	c.1464T>A	exonic	7	stopgain	p.Tyr488*	3104/273832=0.011
113	SERPINB8	NM_001366198.1	c.1121C>T	exonic	7	nonsynonymous	p.Pro374Leu	9/273242=0
114	ABCA12	NM_173076.3	c.1743C>G	exonic	14	nonsynonymous	p.Asp581Glu	246/277138=0.001
115	KRT10	NM_001379366.1	c.158G>A	exonic	1	nonsynonymous	p.Ser53Asn	65/276636=0
116	ALDH3A2	NM_001031806.2	c.17G>C	exonic	1	nonsynonymous	p.Arg6Pro	3/223156=0
117	GJB2	NM_004004.6	c.35delG	exonic	2	frameshift	p.Gly12Valfs*2	1721/275002=0.006
118	ALDH3A2	NM_001031806.2	c.1270C>T	exonic	9	nonsynonymous	p.Pro424Ser	1205/277210=0.004
118	ZMPSTE24	NM_005857.3	c.1235G>A	exonic	10	nonsynonymous	p.Arg412His	11/246164=0
119	GJB3	NM_024009.3	c.196_198delGAC	exonic	2	inframe	p.Asp66del	38/277154=0
120	NIPAL4	NM_001099287.1	c.446C>T	exonic	2	nonsynonymous	p.Thr149Met	294/266230=0.001
121	NIPAL4	NM_001099287.1	c.581C>T	exonic	4	nonsynonymous	p.Thr194Met	1/243716=0
121	SERPINB7	NM_003784.4	c.220T>C	exonic	4	nonsynonymous	p.Ser74Pro	9/247468=0
122	KRT9	NM_000226.4	c.245G>A	exonic	1	nonsynonymous	p.Ser82Asn	628/274158=0.002
123	PNPLA1	NM_001374623.1	c.1464T>A	exonic	7	stopgain	p.Tyr488*	3104/273832=0.011
124	ALOX12B	NM_001139.3	c.280G>A	exonic	2	nonsynonymous	p.Gly94Ser	2588/264690
124	KRT10	NM_001379366.1	c.1443_1457delAAGCTCCGGCGGCGG	exonic	7	inframe	p.Ser482_Gly486del	1/99466=0
124	SPINK5	NM_001127698.2	c.2954T>C	exonic	30	nonsynonymous	p.Val985Ala	982/276828=0.004
124	ST14	NM_021978.4	c.454A>G	exonic	5	nonsynonymous	p.Ile152Val	876/276060=0.003
124	TGM1	NM_000359.3	c.2405A>T	exonic	15	nonsynonymous	p.Asp802Val	262/277042=0.001
125	PNPLA1	NM_001374623.1	c.472T>A	exonic	3	nonsynonymous	p.Cys158Ser	
125	STS	NM_001320751.2	c.1147A>G	exonic	10	nonsynonymous	p.Ile383Val	9/178456=0
126	SPINK5	NM_001127698.2	c.1451G>A	exonic	16	nonsynonymous	p.Arg484Lys	343/276350=0.001
127	CDSN	NM_001264.4	c.782G>T	exonic	2	nonsynonymous	p.Gly261Val	
127	ELOVL4	NM_022726.4	c.800T>C	exonic	6	nonsynonymous	p.Ile267Thr	2004/276984=0.007
127	NIPAL4	NM_001099287.1	c.176C>A	exonic	1	nonsynonymous	p.Ala59Asp	0/242706=0

127	SERPINB7	NM_003784.4	c.992A>C	exonic	8	nonsynonymous	p.Glu331Ala	113/276360=0
128	SERPINB8	NM_001366198.1	c.254T>G	exonic	3	nonsynonymous	p.Leu85Trp	568/277052=0.002
129	GJB2	NM_004004.6	c.23C>T	exonic	2	nonsynonymous	p.Thr8Met	21/275096=0
129	SUMF1	NM_182760.4	c.664G>C	exonic	5	nonsynonymous	p.Gly222Arg	450/277142=0.002
130	GJB6	NM_001370092.1	c.212T>C	exonic	5	nonsynonymous	p.Val71Ala	110/277050=0
131	KRT1	NM_006121.4	c.1912A>G	exonic	9	nonsynonymous	p.Thr638Ala	35/276622=0
132	SULT2B1	NM_004605.2	c.600+3G>A	intronic	4			
133	SERPINB7	NM_003784.4	c.220T>C	exonic	4	nonsynonymous	p.Ser74Pro	9/247468=0
134	SULT2B1	NM_004605.2	c.107T>C	exonic	1	nonsynonymous	p.Leu36Ser	1287/274634=0.005
135	ABHD5	NM_001365649.1	c.505C>G	exonic	4	nonsynonymous	p.Pro169Ala	21/277218=0
136	GJB4	NM_153212.3	c.386G>A	exonic	2	stopgain	p.Trp129*	59/277112=0
137	ALOXE3	NM_001165960.1	c.1076C>T	exonic	6	nonsynonymous	p.Ala359Val	51/246268=0
137	GJB4	NM_153212.3	c.153delT	exonic	2	frameshift	p.Phe51Leufs*57	1464/276392=0.005
137	KRT10	NM_001379366.1	c.710+6T>C	intronic	2			5/277170=0
137	PEX7	NM_000288.4	c.961A>T	exonic	10	nonsynonymous	p.Ile321Phe	
137	SERPINB8	NM_001366198.1	c.254T>G	exonic	3	nonsynonymous	p.Leu85Trp	568/277052=0.002
137	SNAP29	NM_004782.4	c.113C>T	exonic	1	nonsynonymous	p.Pro38Leu	139/246926=0.001
137	SUMF1	NM_182760.4	c.59T>G	exonic	1	nonsynonymous	p.Leu20Ar	988/267246=0.004
138	ALOXE3	NM_001165960.1	c.989T>C	exonic	6	nonsynonymous	p.Ile330Thr	4/246268=0
139	KRT2	NM_000423.3	c.317G>A	exonic	1	nonsynonymous	p.Ser106Asn	641/254108=0.003
139	TGM1	NM_000359.3	c.90_95dupGCCAGA	exonic	2	inframe	p.Glu30_Pro31dup	132/276714=0
140	GJB2	NM_004004.6	c.457G>A	exonic	2	nonsynonymous	p.Val153Ile	2433/276862=0.009
140	STS	NM_001320751.2	c.478C>T	exonic	7	nonsynonymous	p.His160Tyr	
140	SULT2B1	NM_004605.2	c.232C>T	exonic	2	nonsynonymous	p.Arg78Cys	252/276636=0.001
141	ABCA12	NM_173076.3	c.1446A>C	exonic	12	nonsynonymous	p.Glu482Asp	29/276918=0
141	GJA1	NM_000165.5	c.758C>T	exonic	2	nonsynonymous	p.Ala253Val	2227/277148=0.008
141	NIPAL4	NM_001099287.1	c.839G>A	exonic	6	nonsynonymous	p.Arg280His	60/276956=0
142	PNPLA1	NM_001374623.1	c.1464T>A	exonic	7	stopgain	p.Tyr488*	3104/273832=0.011
143	ALDH3A2	NM_001031806.2	c.1270C>T	exonic	9	nonsynonymous	p.Pro424Ser	1205/277210=0.004
144	CLDN1	NM_021101.5	c.278T>C	exonic	2	nonsynonymous	p.Ile93Thr	
144	KRT10	NM_001379366.1	c.1650_1667delCAGCAGCTCCGGCGGCGG	exonic	7	inframe	p.Ser551_Gly556del	34/219570=0
145	GJB2	NM_004004.6	c.358_360delGAG	exonic	2	inframe	p.Glu120del	20/275990=0
145	PNPLA1	NM_001374623.1	c.1464T>A	exonic	7	stopgain	p.Tyr488*	3104/273832=0.011
146	ABCA12	NM_173076.3	c.485C>T	exonic	5	nonsynonymous	p.Ala162Val	384/276512=0.001
146	ST14	NM_021978.4	c.508G>A	exonic	5	nonsynonymous	p.Glu170Lys	521/276770=0.002
147	SLC27A4	NM_005094.4	c.1300G>A	exonic	9	nonsynonymous	p.Gly434Ser	9/245968=0
147	VPS33B	NM_018668.4	c.1166G>A	exonic	15	nonsynonymous	p.Arg389Gln	590/277118=0.002
148	ALDH3A2	NM_001031806.2	c.28C>G	exonic	1	nonsynonymous	p.Gln10Glu	806/218946=0.004
148	CYP4F22	NM_173483.4	c.665G>T	exonic	7	nonsynonymous	p.Cys222Phe	
149	CAST	NM_001750.7	c.620C>T	exonic	9	nonsynonymous	p.Pro207Leu	5/246206=0
150	CAST	NM_001750.7	c.1177C>T	exonic	16	nonsynonymous	p.Arg393Cys	34/276754=0
150	GJB3	NM_024009.3	c.293G>A	exonic	2	nonsynonymous	p.Arg98His	23/276870=0

151	GJB2	NM_004004.6	c.35delG	exonic	2	frameshift	p.Gly12Valfs*2	1721/275002=0.006
151	GJB4	NM_153212.3	c.384G>A	exonic	2	stopgain	p.Trp128*	527/277132=0.002
151	LIPN	NM_001102469.1	c.302delG	exonic	3	frameshift	p.Gly101Glufs*7	50/276298=0
152	KRT2	NM_000423.3	c.1750A>G	exonic	9	nonsynonymous	p.Ile584Val	
152	TGM1	NM_000359.3	c.208G>T	exonic	2	nonsynonymous	p.Gly70Cys	
153	GJB2	NM_004004.6	c.467T>A	exonic	2	nonsynonymous	p.Val156Asp	
154	GJB2	NM_004004.6	c.296G>A	exonic	2	nonsynonymous	p.Arg99Lys	
155	PNPLA1	NM_001374623.1	c.1464T>A	exonic	7	stopgain	p.Tyr488*	3104/273832=0.011
156	GJA1	NM_000165.5	c.758C>T	exonic	2	nonsynonymous	p.Ala253Val	2227/277148=0.008
156	GJA1	NM_000165.5	c.157C>T	exonic	2	nonsynonymous	p.Arg53Cys	2/246262=0
156	PHYH	NM_001323080.2	c.301C>G	exonic	6	nonsynonymous	p.Arg101Gly	351/277160=0.001
157	KRT1	NM_006121.4	c.1390G>A	exonic	7	nonsynonymous	p.Asp464Asn	1/246258=0
157	ST14	NM_021978.4	c.2146G>C	exonic	17	nonsynonymous	p.Glu716Gln	
158	ABCA12	NM_173076.3	c.6919A>G	exonic	46	nonsynonymous	p.Ile2307Val	486/277078=0.002
158	ALOX12B	NM_001139.3	c.1431delC	exonic	11	frameshift	p.Asp477Glufs*37	1/245856=0
159	GJB2	NM_004004.6	c.101T>C	exonic	2	nonsynonymous	p.Met34Thr	2487/276420=0.009
159	ST14	NM_021978.4	c.967C>T	exonic	8	nonsynonymous	p.Arg323Trp	2/246262=0
159	ST14	NM_021978.4	c.1975A>G	exonic	16	nonsynonymous	p.Ile659Val	33/245026=0
160	ABCA12	NM_173076.3	c.6919A>G	exonic	46	nonsynonymous	p.Ile2307Val	486/277078=0.002
160	GJB4	NM_153212.3	c.153delT	exonic	2	frameshift	p.Phe51Leufs*57	1464/276392=0.005
160	KRT2	NM_000423.3	c.767A>G	exonic	2	nonsynonymous	p.Asn256Ser	110/277226=0
160	PNPLA1	NM_001374623.1	c.985T>C	exonic	6	nonsynonymous	p.Ser329Pro	615/276868=0.002
161	ABCA12	NM_173076.3	c.6919A>G	exonic	46	nonsynonymous	p.Ile2307Val	486/277078=0.002
161	PNPLA1	NM_001374623.1	c.1464T>A	exonic	7	stopgain	p.Tyr488*	3104/273832=0.011
162	STS	NM_001320751.2	c.1168C>T	exonic	10	nonsynonymous	p.Arg390Cys	4/178511=0
163	ALOXE3	NM_001165960.1	c.1483C>T	exonic	9	nonsynonymous	p.Pro495Ser	
163	PNPLA1	NM_001374623.1	c.1464T>A	exonic	7	stopgain	p.Tyr488*	3104/273832=0.011
164	SPINK5	NM_001127698.2	c.1362G>C	exonic	15	nonsynonymous	p.Glu454Asp	1/245736=0
165	SPINK5	NM_001127698.2	c.1362G>C	exonic	15	nonsynonymous	p.Glu454Asp	1/245736=0
166	GJB2	NM_004004.6	c.358_360delGAG	exonic	2	inframe	p.Glu120del	20/275990=0
166	GJB3	NM_024009.3	c.293G>A	exonic	2	nonsynonymous	p.Arg98His	23/276870=0
167	GJB6	NM_001370092.1	c.607A>G	exonic	5	nonsynonymous	p.Met203Val	247/277132=0.001
167	PHYH	NM_001323080.2	c.56C>T	exonic	4	nonsynonymous	p.Thr19Met	367/277256=0.001
167	PHYH	NM_001323080.2	c.403G>A	exonic	7	nonsynonymous	p.Gly135Arg	2/246256=0
167	SULT2B1	NM_004605.2	c.232C>T	exonic	2	nonsynonymous	p.Arg78Cys	252/276636=0.001
168	ABCA12	NM_173076.3	c.6919A>G	exonic	46	nonsynonymous	p.Ile2307Val	486/277078=0.002
168	GJB3	NM_024009.3	c.670C>T	exonic	2	stopgain	p.Arg224*	11/276538=0
168	KRT9	NM_000226.4	c.49G>A	exonic	1	nonsynonymous	p.Gly17Ser	
169	ST14	NM_021978.4	c.2553G>C	exonic	19	nonsynonymous	p.Glu851Asp	6/276830=0
170	VPS33B	NM_018668.4	c.1307A>G	exonic	18	nonsynonymous	p.Asn436Ser	15/277084=0
171	GJA1	NM_000165.5	c.758C>T	exonic	2	nonsynonymous	p.Ala253Val	2227/277148=0.008
171	LIPN	NM_001102469.1	c.772G>A	exonic	6	nonsynonymous	p.Glu258Lys	316/215004=0.001

171	SPINK5	NM_001127698.2	c.2852A>G	exonic	30	nonsynonymous	p.Asn951Ser	1264/277148=0.005
172	ABCA12	NM_173076.3	c.6704A>C	exonic	45	nonsynonymous	p.Glu2235Ala	184/277156=0.001
172	ALOX12B	NM_001139.3	c.715A>G	exonic	6	nonsynonymous	p.Ile239Val	24/277224=0
172	ELOVL4	NM_022726.4	c.800T>C	exonic	6	nonsynonymous	p.Ile267Thr	2004/276984=0.007
172	SLC27A4	NM_005094.4	c.952C>T	exonic	7	nonsynonymous	p.Arg318Trp	28/277118=0
173	LIPN	NM_001102469.1	c.772G>A	exonic	6	nonsynonymous	p.Glu258Lys	316/215004=0.001
174	VPS33B	NM_018668.4	c.1274G>A	exonic	18	nonsynonymous	p.Ser425Asn	350/277058=0.001
175	ALOXE3	NM_001165960.1	c.1454T>A	exonic	9	nonsynonymous	p.Leu485Gln	
176	SERPINB7	NM_003784.4	c.220T>C	exonic	4	nonsynonymous	p.Ser74Pro	9/247468=0
177	STS	NM_001320751.2	c.1274A>G	exonic	10	nonsynonymous	p.Asp425Gly	184/199731=0.001
178	KRT1	NM_006121.4	c.1693A>G	exonic	9	nonsynonymous	p.Ser565Gly	
179	NIPAL4	NM_001099287.1	c.730G>T	exonic	5	nonsynonymous	p.Val244Phe	170/254010=0.001
179	SLC27A4	NM_005094.4	c.1788C>G	exonic	13	nonsynonymous	p.Phe596Leu	3/246250=0
179	TGM1	NM_000359.3	c.550C>T	exonic	4	nonsynonymous	p.Pro184Ser	183/277094=0.001
180	ALOX12B	NM_001139.3	c.526G>A	exonic	4	nonsynonymous	p.Glu176Lys	92/276916=0
180	ALOXE3	NM_001165960.1	c.1843C>T	exonic	12	nonsynonymous	p.His615Tyr	
180	GJB4	NM_153212.3	c.314A>G	exonic	2	nonsynonymous	p.His105Arg	10/277130=0
181	ALOX12B	NM_001139.3	c.526G>A	exonic	4	nonsynonymous	p.Glu176Lys	92/276916=0
181	CLDN1	NM_021101.5	c.136A>T	exonic	1	nonsynonymous	p.Met46Leu	15/246242=0
182	CLDN1	NM_021101.5	c.136A>T	exonic	1	nonsynonymous	p.Met46Leu	15/246242=0
183	LIPN	NM_001102469.1	c.633T>G	exonic	5	nonsynonymous	p.Ile211Met	12/275930=0
184	ABCA12	NM_173076.3	c.539T>C	exonic	6	nonsynonymous	p.Ile180Thr	3/245112=0
184	GJB2	NM_004004.6	c.101T>C	exonic	2	nonsynonymous	p.Met34Thr	2487/276420=0.009
184	SUMF1	NM_182760.4	c.131G>A	exonic	1	nonsynonymous	p.Gly44Glu	
185	GJB2	NM_004004.6	c.35delG	exonic	2	frameshift	p.Gly12Valfs*2	1721/275002=0.006
186	ST14	NM_021978.4	c.145A>T	exonic	2	stopgain	p.Lys49*	
187	TGM1	NM_000359.3	c.550C>T	exonic	4	nonsynonymous	p.Pro184Ser	183/277094=0.001
188	ABCA12	NM_173076.3	c.485C>T	exonic	5	nonsynonymous	p.Ala162Val	384/276512=0.001
189	TGM1	NM_000359.3	c.208G>T	exonic	2	nonsynonymous	p.Gly70Cys	
190	ALOXE3	NM_001165960.1	c.2102C>A	exonic	14	nonsynonymous	p.Thr701Asn	102/276992=0
190	ALOXE3	NM_001165960.1	c.2404C>T	exonic	16	nonsynonymous	p.Arg802Trp	239/277182=0.001
190	CYP4F22	NM_173483.4	c.463C>T	exonic	6	nonsynonymous	p.His155Tyr	121/277112=0
190	NIPAL4	NM_001099287.1	c.397G>A	exonic	2	nonsynonymous	p.Gly133Ser	9/277110=0
191	ABCA12	NM_173076.3	c.501G>C	exonic	5	nonsynonymous	p.Leu167Phe	
192	KRT2	NM_000423.3	c.767A>G	exonic	2	nonsynonymous	p.Asn256Ser	110/277226=0
192	NIPAL4	NM_001099287.1	c.730G>T	exonic	5	nonsynonymous	p.Val244Phe	170/254010=0.001
192	SERPINB7	NM_003784.4	c.715G>A	exonic	7	nonsynonymous	p.Val239Ile	20/276514=0
192	SPINK5	NM_001127698.2	c.1451G>A	exonic	16	nonsynonymous	p.Arg484Lys	343/276350=0.001
193	ALOXE3	NM_001165960.1	c.2510T>C	exonic	16	nonsynonymous	p.Ile837Thr	333/277202=0.001
193	CERS3	NM_001290341.2	c.914A>G	exonic	13	nonsynonymous	p.His305Arg	3292/264968=0.012
193	NIPAL4	NM_001099287.1	c.397G>A	exonic	2	nonsynonymous	p.Gly133Ser	9/277110=0
193	ST14	NM_021978.4	c.508G>A	exonic	5	nonsynonymous	p.Glu170Lys	521/276770=0.002

194	ALOXE3	NM_001165960.1	c.2510T>C	exonic	16	nonsynonymous	p.Ile837Thr	333/277202=0.001
194	CAST	NM_001750.7	c.1283C>T	exonic	17	nonsynonymous	p.Thr428Met	909/277064=0.003
194	CYP4F22	NM_173483.4	c.463C>T	exonic	6	nonsynonymous	p.His155Tyr	121/277112=0
194	ST14	NM_021978.4	c.2406+4G>C	intronic	18			193/212654=0.001
195	ABCA12	NM_173076.3	c.485C>T	exonic	5	nonsynonymous	p.Ala162Val	384/276512=0.001
195	SULT2B1	NM_004605.2	c.107T>C	exonic	1	nonsynonymous	p.Leu36Ser	1287/274634=0.005
196	GJB3	NM_024009.3	c.659A>T	exonic	2	nonsynonymous	p.Lys220Met	1/246042=0
197	NIPAL4	NM_001099287.1	c.176C>A	exonic	1	nonsynonymous	p.Ala59Asp	
198	GJB2	NM_004004.6	c.35delG	exonic	2	frameshift	p.Gly12Valfs*2	1721/275002=0.006
198	SLC27A4	NM_005094.4	c.742G>A	exonic	5	nonsynonymous	p.Gly248Ser	
199	ALOXE3	NM_001165960.1	c.2510T>C	exonic	16	nonsynonymous	p.Ile837Thr	333/277202=0.001
199	CYP4F22	NM_173483.4	c.851G>A	exonic	8	nonsynonymous	p.Arg284Gln	9/276864=0
200	ABCA12	NM_173076.3	c.1141G>C	exonic	10	nonsynonymous	p.Val381Leu	239/277242=0.001
201	ALOXE3	NM_001165960.1	c.2510T>C	exonic	16	nonsynonymous	p.Ile837Thr	333/277202=0.001
201	GJB4	NM_153212.3	c.389C>T	exonic	2	nonsynonymous	p.Thr130Met	30/277058=0
202	GJB2	NM_004004.6	c.35delG	exonic	2	frameshift	p.Gly12Valfs*2	1721/275002=0.006
202	SULT2B1	NM_004605.2	c.867G>A	exonic	6	nonsynonymous	p.Met289Ile	270/262312=0.001
203	VPS33B	NM_018668.4	c.1837A>T	exonic	23	nonsynonymous	p.Ser613Cys	
204	ALDH3A2	NM_001031806.1	c.1270C>T	exonic	8	nonsynonymous	p.Pro424Ser	1205/277210=0.004
204	ELOVL4	NM_022726.4	c.814G>C	exonic	6	nonsynonymous	p.Glu272Gln	2735/277052=0.01
204	GJB2	NM_004004.6	c.35delG	exonic	2	frameshift	p.Gly12Valfs*2	1721/275002=0.006
204	NIPAL4	NM_001099287.1	c.296T>C	exonic	2	nonsynonymous	p.Val99Ala	783/277214=0.003
204	SULT2B1	NM_004605.2	c.867G>A	exonic	6	nonsynonymous	p.Met289Ile	270/262312=0.001
205	GJB2	NM_004004.6	c.35delG	exonic	2	frameshift	p.Gly12Valfs*2	1721/275002=0.006

ABCA12	c.6593C>T	p.(Thr2198Ile)	VUS		
ABCA12	c.6704A>C	p.(Glu2235Ala)	Benign		
ALOX3	c.122C>A	p.(Ala41Asp)	VUS		
GJB2	c.663G>C	p.Lys221Asn	VUS		PP2
GJB4	c.500A>G	p.(Glu167Gly)	VUS		
KRT1	c.1724A>G	p.(His575Arg)	VUS		
KRT1	c.1669A>G	p.(Ser557Gly)	Benign		
PNPLA1	c.1464T>A	p.(Tyr488*)	Benign		
RIN2	c.364C>T	p.(His122Trp)	VUS		
RTEL1	c.2920G>A	p.(Gly974Ser)	VUS		
ST14	c.2146G>C	p.(Glu716Gln)	VUS		
SULT2B1	c.912G>A	p.(Met304Ile)	Likely benign		
SUMF1	c.376A>C	p.(Met126Leu)	VUS		
TGM5	c.850G>A	p.(Val284Ile)	VUS		
TGM1	c.1033G>A	p.(Asp345Asn)	VUS	Class 3	
TGM1	c.2405A>T	p.(Asp802Val)	Benign	Class 1	

	PM2
	PM2
	PM2
	PM2
	PM2
	PM2
	PM2
	PM2
	PM2
	PM2
PM1	PM2

23.8

26.6
