# Supplementary material for: Comprehensive Molecular Analysis of Disease-Related Genes as First-Tier Test for Early Diagnosis, Classification, and Management of Patients Affected by Nonsyndromic Ichthyosis
Source: Biomedicines. 2024 May 17;12(5):1112. doi: 10.3390/biomedicines12051112 (PMC11117922; doi:10.3390/biomedicines12051112)
Supplement: Supplementary file 1 [file biomedicines-12-01112-s001.zip › biomedicines-2972903-SM final/Supplementary files/SUPPLEMENTARY Table S3.pdf]

Table S3. Number of different rare variants identified in ichthyosis-related genes of 300 unaffected individuals

GENE	GenBank #	Nucleotide variant	gene position	Exon	Presumed effect	Presumed protein variant	gnomAD frequency
ABCA12	NM_173076.3	c.300T>G	exonic	3	nonsynonymous	p.Asp100Glu	6/276566=0
ABCA12	NM_173076.3	c.346G>T	exonic	4	nonsynonymous	p.Asp116Tyr	7/245398=0
ABCA12	NM_173076.3	c.485C>T	exonic	5	nonsynonymous	p.Ala162Val	384/276512=0.001
ABCA12	NM_173076.3	c.501G>C	exonic	5	nonsynonymous	p.Leu167Phe	
ABCA12	NM_173076.3	c.539T>C	exonic	6	nonsynonymous	p.Ile180Thr	3/245112=0
ABCA12	NM_173076.3	c.1141G>C	exonic	10	nonsynonymous	p.Val381Leu	239/277242=0.001
ABCA12	NM_173076.3	c.1222T>C	exonic	11	nonsynonymous	p.Ser408Pro	322/276876=0.001
ABCA12	NM_173076.3	c.1446A>C	exonic	12	nonsynonymous	p.Glu482Asp	29/276918=0
ABCA12	NM_173076.3	c.1475A>G	exonic	12	nonsynonymous	p.Asn492Ser	5/276916=0
ABCA12	NM_173076.3	c.1743C>G	exonic	14	nonsynonymous	p.Asp581Glu	246/277138=0.001
ABCA12	NM_173076.3	c.1816G>A	exonic	15	nonsynonymous	p.Asp606Asn	2/245978=0
ABCA12	NM_173076.3	c.2129A>G	exonic	17	nonsynonymous	p.Tyr710Cys	1/246028=0
ABCA12	NM_173076.3	c.2243G>A	exonic	17	nonsynonymous	p.Arg748Lys	6/245968=0
ABCA12	NM_173076.3	c.3098T>C	exonic	22	nonsynonymous	p.Ile1033Thr	2/245894=0
ABCA12	NM_173076.3	c.3481A>T	exonic	24	nonsynonymous	p.Met1161Leu	363/277160=0.001
ABCA12	NM_173076.3	c.4618G>T	exonic	31	nonsynonymous	p.Ala1540Ser	9/245910=0
ABCA12	NM_173076.3	c.5051T>C	exonic	33	nonsynonymous	p.Ile1684Thr	4/245586=0
ABCA12	NM_173076.3	c.5617G>A	exonic	37	nonsynonymous	p.Val1873Ile	670/276578=0.002
ABCA12	NM_173076.3	c.6208G>A	exonic	42	nonsynonymous	p.Val2070Ile	514/276952=0.002
ABCA12	NM_173076.3	c.6704A>C	exonic	45	nonsynonymous	p.Glu2235Ala	184/277156=0.001
ABCA12	NM_173076.3	c.6919A>G	exonic	46	nonsynonymous	p.Ile2307Val	486/277078=0.002
ABCA12	NM_173076.3	c.7631C>T	exonic	52	nonsynonymous	p.Thr2544Ile	379/276596=0.001
ABHD5	NM_001365649.1	c.22A>G	exonic	3	nonsynonymous	p.Thr8Ala	12/237020=0
ABHD5	NM_001365649.1	c.505C>G	exonic	4	nonsynonymous	p.Pro169Ala	21/277218=0
ABHD5	NM_001365649.1	c.883G>T	exonic	7	stopgain	p.Glu295*	1/121404=0
ALDH3A2	NM_001031806.2	c.119A>G	exonic	1	nonsynonymous	p.Asp40Gly	155/218008=0.001
ALDH3A2	NM_001031806.2	c.28C>G	exonic	1	nonsynonymous	p.Gln10Glu	806/218946=0.004
ALDH3A2	NM_001031806.2	c.17G>C	exonic	2	nonsynonymous	p.Arg6Pro	3/223156=0
ALDH3A2	NM_001031806.2	c.661G>A	exonic	4	nonsynonymous	p.Asp221Asn	1/244348=0
ALDH3A2	NM_001031806.2	c.1270C>T	exonic	9	nonsynonymous	p.Pro424Ser	1205/277210=0.004
ALOX12B	NM_001139.3	c.280G>A	exonic	2	nonsynonymous	p.Gly94Ser	2588/264690
ALOX12B	NM_001139.3	c.380C>T	exonic	3	nonsynonymous	p.Pro127Leu	66/276792=0
ALOX12B	NM_001139.3	c.526G>A	exonic	4	nonsynonymous	p.Glu176Lys	92/276916=0
ALOX12B	NM_001139.3	c.556A>T	exonic	5	nonsynonymous	p.Ile186Phe	0
ALOX12B	NM_001139.3	c.715A>G	exonic	6	nonsynonymous	p.Ile239Val	24/277224=0
ALOX12B	NM_001139.3	c.1156C>T	exonic	9	nonsynonymous	p.Arg386Cys	9/276770=0
ALOX12B	NM_001139.3	c.1431delC	exonic	11	frameshift	p.Asp477Glufs*37	1/245856=0
ALOX12B	NM_001139.3	c.1565C>T	exonic	12	nonsynonymous	p.Pro522Leu	277/277210=0.001
ALOXE3	NM_001165960.1	c.30G>T	exonic	1	nonsynonymous	p.Leu10Phe	
ALOXE3	NM_001165960.1	c.62C>T	exonic	1	nonsynonymous	p.Pro21Leu	16/168304=0
ALOXE3	NM_001165960.1	c.280C>G	exonic	2	nonsynonymous	p.Pro94Ala	143/182810=0.001
ALOXE3	NM_001165960.1	c.809G>A	exonic	4	nonsynonymous	p.Arg270Gln	7/246260=0
ALOXE3	NM_001165960.1	c.989T>C	exonic	6	nonsynonymous	p.Ile330Thr	4/246268=0

ALOXE3	NM_001165960.1	c.1076C>T	exonic	6	nonsynonymous	p.Ala359Val	51/246268=0
ALOXE3	NM_001165960.1	c.1454T>A	exonic	9	nonsynonymous	p.Leu485Gln	
ALOXE3	NM_001165960.1	c.1483C>T	exonic	9	nonsynonymous	p.Pro495Ser	
ALOXE3	NM_001165960.1	c.1843C>T	exonic	12	nonsynonymous	p.His615Tyr	
ALOXE3	NM_001165960.1	c.2102C>A	exonic	14	nonsynonymous	p.Thr701Asn	102/276992=0
ALOXE3	NM_001165960.1	c.2404C>T	exonic	16	nonsynonymous	p.Arg802Trp	239/277182=0.001
ALOXE3	NM_001165960.1	c.2510T>C	exonic	16	nonsynonymous	p.Ile837Thr	333/277202=0.001
CDSN	NM_001264.4	c.32G>A	exonic	1	nonsynonymous	p.Arg11His	790/239488=0.003
CDSN	NM_001264.4	c.1302C>A	exonic	2	nonsynonymous	p.Ser434Arg	916/276996=0.003
CDSN	NM_001264.4	c.475A>G	exonic	2	nonsynonymous	p.Ser159Gly	47/276174=0
CDSN	NM_001264.4	c.782G>T	exonic	2	nonsynonymous	p.Gly261Val	
CAST	NM_001750.7	c.200C>T	exonic	3	nonsynonymous	p.Ser67Leu	12/276074=0
CAST	NM_001750.7	c.620C>T	exonic	9	nonsynonymous	p.Pro207Leu	5/246206=0
CAST	NM_001750.7	c.775A>G	exonic	11	nonsynonymous	p.Thr259Ala	943/276512=0.003
CAST	NM_001750.7	c.925A>C	exonic	14	nonsynonymous	p.Ile309Leu	2994/276670=0.011
CAST	NM_001750.7	c.986C>G	exonic	14	nonsynonymous	p.Ala329Gly	74/276982=0
CAST	NM_001750.7	c.1177C>T	exonic	16	nonsynonymous	p.Arg393Cys	34/276754=0
CAST	NM_001750.7	c.1207G>C	exonic	17	nonsynonymous	p.Ala403Pro	1334/276854=0.005
CAST	NM_001750.7	c.1283C>T	exonic	17	nonsynonymous	p.Thr428Met	909/277064=0.003
CAST	NM_001750.7	c.1835A>G	exonic	25	nonsynonymous	p.Lys612Arg	585/265502=0.002
CLDN1	NM_021101.5	c.136A>T	exonic	1	nonsynonymous	p.Met46Leu	15/246242=0
CLDN1	NM_021101.5	c.278T>C	exonic	2	nonsynonymous	p.Ile93Thr	
CLDN1	NM_021101.5	c.631G>A	exonic	4	nonsynonymous	p.Val211Met	30/277152=0
CERS3	NM_001290341.2	c.233C>T	exonic	6	nonsynonymous	p.Ser78Leu	
CERS3	NM_001290341.2	c.914A>G	exonic	13	nonsynonymous	p.His305Arg	3292/264968=0.012
CERS3	NM_001290341.2	c.1151G>A	exonic	14	nonsynonymous	p.Arg384Lys	481/277162=0.002
CYP4F22	NM_173483.4	c.109C>T	exonic	3	nonsynonymous	p.Arg37Cys	32/121338=0
CYP4F22	NM_173483.4	c.68C>T	exonic	3	nonsynonymous	p.Ala23Val	45/277126=0
CYP4F22	NM_173483.4	c.463C>T	exonic	6	nonsynonymous	p.His155Tyr	121/277112=0
CYP4F22	NM_173483.4	c.485C>G	exonic	6	nonsynonymous	p.Ala162Gly	47/277122=0
CYP4F22	NM_173483.4	c.665G>T	exonic	7	nonsynonymous	p.Cys222Phe	
CYP4F22	NM_173483.4	c.712G>A	exonic	8	nonsynonymous	p.Ala238Thr	
CYP4F22	NM_173483.4	c.851G>A	exonic	8	nonsynonymous	p.Arg284Gln	9/276864=0
CYP4F22	NM_173483.4	c.1148C>T	exonic	11	nonsynonymous	p.Thr383Ile	
ELOVL4	NM_022726.4	c.243C>G	exonic	2	nonsynonymous	p.Ile81Met	
ELOVL4	NM_022726.4	c.800T>C	exonic	6	nonsynonymous	p.Ile267Thr	2004/276984=0.007
ELOVL4	NM_022726.4	c.814G>C	exonic	6	nonsynonymous	p.Glu272Gln	2735/277052=0.01
GJA1	NM_000165.5	c.1108C>T	exonic	2	nonsynonymous	p.Arg370Cys	3/245356=0
GJA1	NM_000165.5	c.1109G>A	exonic	2	nonsynonymous	p.Arg370His	1/245340=0
GJA1	NM_000165.5	c.157C>T	exonic	2	nonsynonymous	p.Arg53Cys	2/246262=0
GJA1	NM_000165.5	c.758C>T	exonic	2	nonsynonymous	p.Ala253Val	2227/277148=0.008
GJB2	NM_004004.6	c.101T>C	exonic	2	nonsynonymous	p.Met34Thr	2487/276420=0.009
GJB2	NM_004004.6	c.109G>A	exonic	2	nonsynonymous	p.Val37Ile	2011/276450=0.007
GJB2	NM_004004.6	c.23C>T	exonic	2	nonsynonymous	p.Thr8Met	21/275096=0
GJB2	NM_004004.6	c.269T>C	exonic	2	nonsynonymous	p.Leu90Pro	177/277032=0.001

GJB2	NM_004004.6	c.296G>A	exonic	2	nonsynonymous	p.Arg99Lys	
GJB2	NM_004004.6	c.358_360delGAG	exonic	2	inframe	p.Glu120del	20/275990=0
GJB2	NM_004004.6	c.35delG	exonic	2	frameshift	p.Gly12Valfs*2	1721/275002=0.006
GJB2	NM_004004.6	c.457G>A	exonic	2	nonsynonymous	p.Val153Ile	2433/276862=0.009
GJB2	NM_004004.6	c.467T>A	exonic	2	nonsynonymous	p.Val156Asp	
GJB2	NM_004004.6	c.88A>G	exonic	2	nonsynonymous	p.Ile30Val	
GJB3	NM_024009.3	c.196_198delGAC	exonic	2	inframe	p.Asp66del	38/277154=0
GJB3	NM_024009.3	c.293G>A	exonic	2	nonsynonymous	p.Arg98His	23/276870=0
GJB3	NM_024009.3	c.316C>T	exonic	2	nonsynonymous	p.Arg106Cys	40/276618=0
GJB3	NM_024009.3	c.422T>C	exonic	2	nonsynonymous	p.Ile141Thr	
GJB3	NM_024009.3	c.529T>G	exonic	2	nonsynonymous	p.Tyr177Asp	525/276788=0.002
GJB3	NM_024009.3	c.659A>T	exonic	2	nonsynonymous	p.Lys220Met	1/246042=0
GJB3	NM_024009.3	c.670C>T	exonic	2	stopgain	p.Arg224*	11/276538=0
GJB4	NM_153212.3	c.119C>T	exonic	2	nonsynonymous	p.Ala40Val	13/277082=0
GJB4	NM_153212.3	c.153delT	exonic	2	frameshift	p.Phe51Leufs*57	1464/276392=0.005
GJB4	NM_153212.3	c.254C>T	exonic	2	nonsynonymous	p.Thr85Met	8/245960=0
GJB4	NM_153212.3	c.314A>G	exonic	2	nonsynonymous	p.His105Arg	10/277130=0
GJB4	NM_153212.3	c.384G>A	exonic	2	stopgain	p.Trp128*	527/277132=0.002
GJB4	NM_153212.3	c.386G>A	exonic	2	stopgain	p.Trp129*	59/277112=0
GJB4	NM_153212.3	c.389C>T	exonic	2	nonsynonymous	p.Thr130Met	30/277058=0
GJB4	NM_153212.3	c.478C>T	exonic	2	nonsynonymous	p.Arg160Cys	20/276732=0
GJB4	NM_153212.3	c.770C>T	exonic	2	nonsynonymous	p.Ser257Leu	20/275366=0
GJB6	NM_001370092.1	c.212T>C	exonic	5	nonsynonymous	p.Val71Ala	110/277050=0
GJB6	NM_001370092.1	c.607A>G	exonic	5	nonsynonymous	p.Met203Val	247/277132=0.001
GJB6	NM_001370092.1	c.688A>T	exonic	5	nonsynonymous	p.Asn230Tyr	
KRT1	NM_006121.4	c.860T>C	exonic	3	nonsynonymous	p.Ile287Thr	1/246176=0
KRT1	NM_006121.4	c.982A>T	exonic	5	nonsynonymous	p.Thr328Ser	332/277126=0.001
KRT1	NM_006121.4	c.1294C>T	exonic	7	nonsynonymous	p.Arg432Cys	143/277212=0.001
KRT1	NM_006121.4	c.1390G>A	exonic	7	nonsynonymous	p.Asp464Asn	1/246258=0
KRT1	NM_006121.4	c.1693A>G	exonic	9	nonsynonymous	p.Ser565Gly	
KRT1	NM_006121.4	c.1894G>A	exonic	9	nonsynonymous	p.Val632Met	2/245184=0
KRT1	NM_006121.4	c.1912A>G	exonic	9	nonsynonymous	p.Thr638Ala	35/276622=0
KRT10	NM_001379366.1	c.158G>A	exonic	1	nonsynonymous	p.Ser53Asn	65/276636=0
KRT10	NM_001379366.1	c.257G>A	exonic	1	nonsynonymous	p.Arg86His	397/264258=0.002
KRT10	NM_001379366.1	c.71G>A	exonic	1	nonsynonymous	p.Gly24Glu	34/179406=0
KRT10	NM_001379366.1	c.98C>T	exonic	1	nonsynonymous	p.Ser33Phe	72/274952=0
KRT10	NM_001379366.1	c.710+6T>C	intronic	2			5/277170=0
KRT10	NM_001379366.1	c.1443_1457delAAGCTCCGGCGGCGG	exonic	7	inframe	p.Ser482_Gly486del	1/99466=0
KRT10	NM_001379366.1	c.1471_1479delCACGGCGGC	exonic	7	inframe	p.His491_Gly493del	
KRT10	NM_001379366.1	c.1495T>C	exonic	7	nonsynonymous	p.Tyr499His	79/97856=0.001
KRT10	NM_001379366.1	c.1524C>G	exonic	7	nonsynonymous	p.Ser508Arg	153/101426=0.002
KRT10	NM_001379366.1	c.1650_1667delCAGCAGCTCCGGCGGCGG	exonic	7	inframe	p.Ser551_Gly556del	34/219570=0
KRT2	NM_000423.3	c.146G>A	exonic	1	nonsynonymous	p.Gly49Asp	3/242214=0
KRT2	NM_000423.3	c.317G>A	exonic	1	nonsynonymous	p.Ser106Asn	641/254108=0.003
KRT2	NM_000423.3	c.767A>G	exonic	2	nonsynonymous	p.Asn256Ser	110/277226=0

KRT2	NM_000423.3	c.1550C>G	exonic	9	nonsynonymous	p.Ala517Gly	993/277108=0.004
KRT2	NM_000423.3	c.1750A>G	exonic	9	nonsynonymous	p.Ile584Val	
KRT9	NM_000226.4	c.245G>A	exonic	1	nonsynonymous	p.Ser82Asn	628/274158=0.002
KRT9	NM_000226.4	c.49G>A	exonic	1	nonsynonymous	p.Gly17Ser	
KRT9	NM_000226.4	c.1049C>G	exonic	5	nonsynonymous	p.Thr350Ser	
KRT9	NM_000226.4	c.1630G>A	exonic	7	nonsynonymous	p.Gly544Arg	10/179160=0
LIPN	NM_001102469.1	c.302delG	exonic	3	frameshift	p.Gly101Glufs*7	50/276298=0
LIPN	NM_001102469.1	c.326A>C	exonic	3	nonsynonymous	p.Asp109Ala	2/245360=0
LIPN	NM_001102469.1	c.633T>G	exonic	5	nonsynonymous	p.Ile211Met	12/275930=0
LIPN	NM_001102469.1	c.754C>T	exonic	6	nonsynonymous	p.Leu252Phe	872/201394=0.004
LIPN	NM_001102469.1	c.772G>A	exonic	6	nonsynonymous	p.Glu258Lys	316/215004=0.001
LIPN	NM_001102469.1	c.934G>T	exonic	8	nonsynonymous	p.Asp312Tyr	1/152140=0
NIPAL4	NM_001099287.1	c.176C>A	exonic	1	nonsynonymous	p.Ala59Asp	
NIPAL4	NM_001099287.1	c.86C>A	exonic	1	stopgain	p.Ser29*	3/121822=0
NIPAL4	NM_001099287.1	c.238C>A	exonic	2	nonsynonymous	p.Leu80Ile	
NIPAL4	NM_001099287.1	c.296T>C	exonic	2	nonsynonymous	p.Val99Ala	783/277214=0.003
NIPAL4	NM_001099287.1	c.397G>A	exonic	2	nonsynonymous	p.Gly133Ser	9/277110=0
NIPAL4	NM_001099287.1	c.446C>T	exonic	2	nonsynonymous	p.Thr149Met	294/266230=0.001
NIPAL4	NM_001099287.1	c.581C>T	exonic	4	nonsynonymous	p.Thr194Met	1/243716=0
NIPAL4	NM_001099287.1	c.730G>T	exonic	5	nonsynonymous	p.Val244Phe	170/254010=0.001
NIPAL4	NM_001099287.1	c.1105G>A	exonic	6	nonsynonymous	p.Val369Ile	127/276938=0
NIPAL4	NM_001099287.1	c.839G>A	exonic	6	nonsynonymous	p.Arg280His	60/276956=0
NIPAL4	NM_001099287.1	c.965G>A	exonic	6	nonsynonymous	p.Arg322Gln	10/246210=0
PEX7	NM_000288.4	c.377A>C	exonic	4	nonsynonymous	p.Gln126Pr	1104/277164=0.004
PEX7	NM_000288.4	c.961A>T	exonic	10	nonsynonymous	p.Ile321Phe	
PHYH	NM_001323080.2	c.56C>T	exonic	4	nonsynonymous	p.Thr19Met	367/277256=0.001
PHYH	NM_001323080.2	c.301C>G	exonic	6	nonsynonymous	p.Arg101Gly	351/277160=0.001
PHYH	NM_001323080.2	c.403G>A	exonic	7	nonsynonymous	p.Gly135Arg	2/246256=0
PNPLA1	NM_001374623.1	c.116C>G	exonic	1	nonsynonymous	p.Ala39Gly	
PNPLA1	NM_001374623.1	c.383C>T	exonic	2	nonsynonymous	p.Thr128Met	397/276794=0.001
PNPLA1	NM_001374623.1	c.472T>A	exonic	3	nonsynonymous	p.Cys158Ser	
PNPLA1	NM_001374623.1	c.592G>T	exonic	4	nonsynonymous	p.Asp198Tyr	
PNPLA1	NM_001374623.1	c.714+7G>A	intronic	4			2/244890=0
PNPLA1	NM_001374623.1	c.745G>A	exonic	5	nonsynonymous	p.Glu249Lys	813/277218=0.003
PNPLA1	NM_001374623.1	c.922A>G	exonic	6	nonsynonymous	p.Thr308Ala	
PNPLA1	NM_001374623.1	c.985T>C	exonic	6	nonsynonymous	p.Ser329Pro	615/276868=0.002
PNPLA1	NM_001374623.1	c.1464T>A	exonic	7	stopgain	p.Tyr488*	3104/273832=0.011
SERPINB7	NM_003784.4	c.220T>C	exonic	4	nonsynonymous	p.Ser74Pro	9/247468=0
SERPINB7	NM_003784.4	c.715G>A	exonic	7	nonsynonymous	p.Val239Ile	20/276514=0
SERPINB7	NM_003784.4	c.833A>G	exonic	8	nonsynonymous	p.Gln278Arg	42/275758=0
SERPINB7	NM_003784.4	c.992A>C	exonic	8	nonsynonymous	p.Glu331Ala	113/276360=0
SERPINB8	NM_001366198.1	c.254T>G	exonic	3	nonsynonymous	p.Leu85Trp	568/277052=0.002
SERPINB8	NM_001366198.1	c.304C>T	exonic	3	nonsynonymous	p.Pro102Ser	352/276834=0.001
SERPINB8	NM_001366198.1	c.1121C>T	exonic	7	nonsynonymous	p.Pro374Leu	9/273242=0
SERPINB8	NM_001366198.1	c.866T>C	exonic	7	nonsynonymous	p.Ile289Thr	9/246270=0

SERPINB8	NM_001366198.1	c.872C>G	exonic	7	nonsynonymous	p.Ala291Gly	60/277244=0
SERPINB8	NM_001366198.1	c.988G>A	exonic	7	nonsynonymous	p.Ala330Thr	61/276742=0
SNAP29	NM_004782.4	c.113C>T	exonic	1	nonsynonymous	p.Pro38Leu	139/246926=0.001
SNAP29	NM_004782.4	c.130T>C	exonic	1	nonsynonymous	p.Tyr44His	649/251920=0.003
SLC27A4	NM_005094.4	c.250G>A	exonic	3	nonsynonymous	p.Val84Ile	2/245924=0
SLC27A4	NM_005094.4	c.742G>A	exonic	5	nonsynonymous	p.Gly248Ser	
SLC27A4	NM_005094.4	c.952C>T	exonic	7	nonsynonymous	p.Arg318Trp	28/277118=0
SLC27A4	NM_005094.4	c.1300G>A	exonic	9	nonsynonymous	p.Gly434Ser	9/245968=0
SLC27A4	NM_005094.4	c.1415_1417delAGA	exonic	10	inframe	p.Lys472del	8/245700=0
SLC27A4	NM_005094.4	c.1788C>G	exonic	13	nonsynonymous	p.Phe596Leu	3/246250=0
SPINK5	NM_001127698.2	c.677A>G	exonic	9	nonsynonymous	p.Lys226Arg	8/276568=0
SPINK5	NM_001127698.2	c.1362G>C	exonic	15	nonsynonymous	p.Glu454Asp	1/245736=0
SPINK5	NM_001127698.2	c.1451G>A	exonic	16	nonsynonymous	p.Arg484Lys	343/276350=0.001
SPINK5	NM_001127698.2	c.1552C>T	exonic	17	nonsynonymous	p.Arg518Cys	879/277134=0.003
SPINK5	NM_001127698.2	c.1964G>A	exonic	21	nonsynonymous	p.Gly655Asp	760/276990=0.003
SPINK5	NM_001127698.2	c.2243A>G	exonic	24	nonsynonymous	p.Glu748Gly	800/276940=0.003
SPINK5	NM_001127698.2	c.2852A>G	exonic	30	nonsynonymous	p.Asn951Ser	1264/277148=0.005
SPINK5	NM_001127698.2	c.2954T>C	exonic	30	nonsynonymous	p.Val985Ala	982/276828=0.004
SPINK5	NM_001127698.2	c.3167T>G	exonic	32	nonsynonymous	p.Met1056Arg	26/246178=0
SPINK5	NM_001127698.2	c.3256G>A	exonic	33	nonsynonymous	p.Ala1086Thr	9/277070=0
ST14	NM_021978.4	c.145A>T	exonic	2	stopgain	p.Lys49*	
ST14	NM_021978.4	c.454A>G	exonic	5	nonsynonymous	p.Ile152Val	876/276060=0.003
ST14	NM_021978.4	c.508G>A	exonic	5	nonsynonymous	p.Glu170Lys	521/276770=0.002
ST14	NM_021978.4	c.800C>A	exonic	7	nonsynonymous	p.Ser267Tyr	2/240850=0
ST14	NM_021978.4	c.830C>T	exonic	7	nonsynonymous	p.Thr277Met	168/254380=0.001
ST14	NM_021978.4	c.967C>T	exonic	8	nonsynonymous	p.Arg323Trp	2/246262=0
ST14	NM_021978.4	c.1034G>A	exonic	9	nonsynonymous	p.Arg345His	302/277184=0.001
ST14	NM_021978.4	c.1975A>G	exonic	16	nonsynonymous	p.Ile659Val	33/245026=0
ST14	NM_021978.4	c.2146G>C	exonic	17	nonsynonymous	p.Glu716Gln	
ST14	NM_021978.4	c.2406+4G>C	intronic	18			193/212654=0.001
ST14	NM_021978.4	c.2553G>C	exonic	19	nonsynonymous	p.Glu851Asp	6/276830=0
STS	NM_001320751.2	c.478C>T	exonic	7	nonsynonymous	p.His160Tyr	
STS	NM_001320751.2	c.1147A>G	exonic	10	nonsynonymous	p.Ile383Val	9/178456=0
STS	NM_001320751.2	c.1168C>T	exonic	10	nonsynonymous	p.Arg390Cys	4/178511=0
STS	NM_001320751.2	c.1274A>G	exonic	10	nonsynonymous	p.Asp425Gly	184/199731=0.001
SULT2B1	NM_004605.2	c.107T>C	exonic	1	nonsynonymous	p.Leu36Ser	1287/274634=0.005
SULT2B1	NM_004605.2	c.232C>T	exonic	2	nonsynonymous	p.Arg78Cys	252/276636=0.001
SULT2B1	NM_004605.2	c.600+3G>A	intronic	4			
SULT2B1	NM_004605.2	c.673G>A	exonic	5	nonsynonymous	p.Val225Ile	220/272762=0.001
SULT2B1	NM_004605.2	c.668G>A	exonic	5	nonsynonymous	p.Gly223Asp	3/272700=0
SULT2B1	NM_004605.2	c.1045C>T	exonic	6	nonsynonymous	p.Pro349Ser	100/144788=0.001
SULT2B1	NM_004605.2	c.867G>A	exonic	6	nonsynonymous	p.Met289Ile	270/262312=0.001
SUMF1	NM_182760.4	c.131G>A	exonic	1	nonsynonymous	p.Gly44Glu	
SUMF1	NM_182760.4	c.59T>G	exonic	1	nonsynonymous	p.Leu20Ar	988/267246=0.004
SUMF1	NM_182760.4	c.664G>C	exonic	5	nonsynonymous	p.Gly222Arg	450/277142=0.002

SUMF1	NM_182760.4	c.935T>C	exonic	7	nonsynonymous	p.Val312Ala	
TGM1	NM_000359.3	c.208G>T	exonic	2	nonsynonymous	p.Gly70Cys	
TGM1	NM_000359.3	c.61A>G	exonic	2	nonsynonymous	p.Thr21Ala	372/276110=0.001
TGM1	NM_000359.3	c.90_95dupGCCAGA	exonic	2	inframe	p.Glu30_Pro31dup	132/276714=0
TGM1	NM_000359.3	c.359C>T	exonic	3	nonsynonymous	p.Ser120Leu	7/245944=0
TGM1	NM_000359.3	c.746C>T	exonic	4	nonsynonymous	p.Pro249Leu	
TGM1	NM_000359.3	c.550C>T	exonic	4	nonsynonymous	p.Pro184Ser	183/277094=0.001
TGM1	NM_000359.3	c.680A>G	exonic	4	nonsynonymous	p.Gln227Arg	
TGM1	NM_000359.3	c.920G>A	exonic	6	nonsynonymous	p.Arg307Gln	124/275156=0
TGM1	NM_000359.3	c.1492-19T>A	intronic	11			7/245872=0
TGM1	NM_000359.3	c.1717C>T	exonic	12	nonsynonymous	p.Arg573Trp	7/277016=0
TGM1	NM_000359.3	c.2338G>A	exonic	15	nonsynonymous	p.Gly780SeR	1/246154=0
TGM1	NM_000359.3	c.2405A>T	exonic	15	nonsynonymous	p.Asp802Val	262/277042=0.001
VPS33B	NM_018668.4	c.97-3C>T	intronic	2			
ZMPSTE24	NM_005857.3	c.1106G>A	exonic	9	nonsynonymous	p.Arg369Gln	199/277132=0.001
ZMPSTE24	NM_005857.3	c.1235G>A	exonic	10	nonsynonymous	p.Arg412His	11/246164=0
VPS33B	NM_018668.4	c.1166G>A	exonic	15	nonsynonymous	p.Arg389Gln	590/277118=0.002
VPS33B	NM_018668.4	c.1274G>A	exonic	18	nonsynonymous	p.Ser425Asn	350/277058=0.001
VPS33B	NM_018668.4	c.1307A>G	exonic	18	nonsynonymous	p.Asn436Ser	15/277084=0
VPS33B	NM_018668.4	c.1837A>T	exonic	23	nonsynonymous	p.Ser613Cys	
